# Lewis Acidic Aluminosilicates: Synthesis, ^27^Al MQ/MAS NMR, and DFT-Calculated ^27^Al NMR Parameters

**DOI:** 10.1021/acs.inorgchem.3c04035

**Published:** 2024-01-25

**Authors:** Martin Kejik, Jiri Brus, Lukas Jeremias, Lucie Simonikova, Zdenek Moravec, Libor Kobera, Ales Styskalik, Craig E. Barnes, Jiri Pinkas

**Affiliations:** † Department of Chemistry, Faculty of Science, Masaryk University, Kotlarska 2, Brno CZ-61137, Czech Republic; ‡ Department of NMR Spectroscopy, Institute of Macromolecular Chemistry, Czech Academy of Sciences, Heyrovskeho nam. 2, Prague CZ-16206, Czech Republic; § Department of Chemistry and Biochemistry, Mendel University in Brno, Brno CZ-61300, Czech Republic; ∥ Department of Chemistry, University of Tennessee, Knoxville, Tennessee 37996-1600, United States

## Abstract

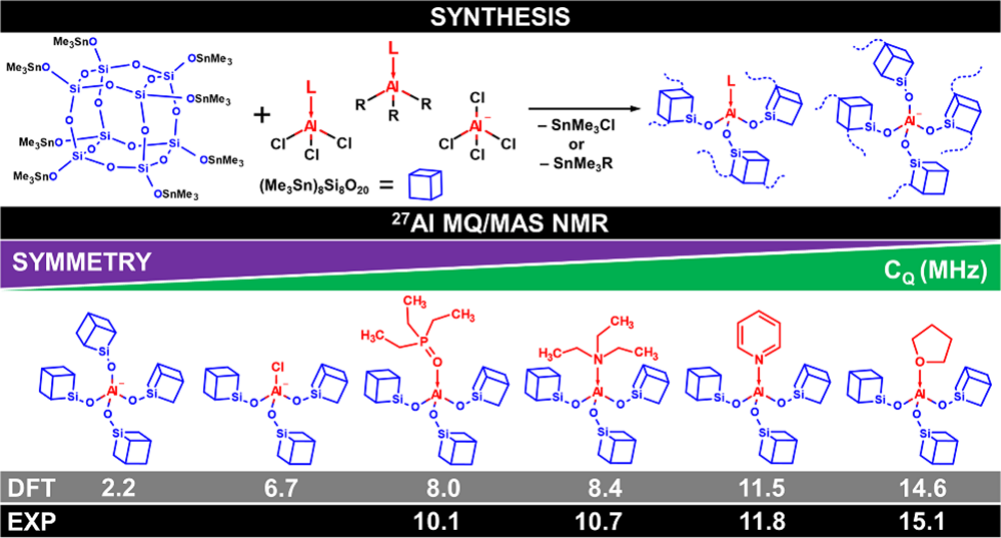

Porous aluminosilicates are functional materials of paramount importance as Lewis acid catalysts in the synthetic industry, yet the participating aluminum species remain poorly studied. Herein, a series of model aluminosilicate networks containing [L–AlO_3_] (L = THF, Et_3_N, pyridine, triethylphosphine oxide (TEPO)) and [AlO_4_]^−^ centers were prepared through nonhydrolytic sol–gel condensation reactions of the spherosilicate building block (Me_3_Sn)_8_Si_8_O_20_ with L–AlX_3_ (X = Cl, Me, Et) and [Me_4_N] [AlCl_4_] compounds in THF or toluene. The substoichiometric dosage of the Al precursors ensured complete condensation and uniform incorporation, with the bulky spherosilicate forcing a separation between neighboring aluminum centers. The materials were characterized by ^1^H, ^13^C, ^27^Al, ^29^Si, and ^31^P MAS NMR and FTIR spectroscopies, ICP-OES, gravimetry, and N_2_ adsorption porosimetry. The resulting aluminum centers were resolved by ^27^Al TQ/MAS NMR techniques and assigned based on their spectroscopic parameters obtained by peak fitting (δ_iso_, *C*_Q_, η) and their correspondence to the values calculated on model structures by DFT methods. A clear correlation between the decrease in the symmetry of the Al centers and the increase of the observed *C*_Q_ was established with values spanning from 4.4 MHz for distorted [AlO_4_]^−^ to 15.1 MHz for [THF–AlO_3_]. Products containing exclusively [TEPO–AlO_3_] or [AlO_4_]^−^ centers could be obtained (single-site materials). For L = THF, Et_3_N, and pyridine, the [AlO_4_]^−^ centers were formed together with the expected [L–AlO_3_] species, and a viable mechanism for the unexpected emergence of [AlO_4_]^−^ was proposed.

## Abstract 2

Model aluminosilicate networks containing [L−AlO_3_] (L = THF, Et_3_N, pyridine, triethylphosphine oxide) and [AlO_4_]^−^ sites were prepared through nonhydrolytic sol−gel condensation reactions of the spherosilicate building block (Me_3_Sn)_8_Si_8_O_20_ with L−AlX_3_ (X = Cl, Me, Et) and [Me_4_N] [AlCl_4_]. Solid-state ^27^Al MQ/MAS NMR analysis supported by DFT calculations allowed for the resolution of the individual aluminum species and established the crucial inverse correlation between the site symmetry and the observed quadrupolar coupling constant (*C*_Q_).

## Introduction

Aluminosilicates are industrially indispensable materials with far-reaching global commercial importance. Besides their more mundane use in construction materials and ceramics, their porous forms, most notably zeolites,^1^ represent the backbone of industrial heterogeneous acid catalysis. Zeolites are crystalline microporous aluminosilicate networks where some of the [SiO_4_] tetrahedra are replaced by [AlO_4_]^−^, necessitating charge compensation by a loosely bound cation in the pore space. Protonation gives rise to well-understood intraframework Bro̷nsted acid sites of the Si(μ^2^–OH)Al type,^2^ which were in the past believed to be responsible for all the catalytic properties of these materials. More recently, the presence and importance of Lewis acid sites have been uncovered and intensively studied.^3−5^ Such sites are not ordinarily present in freshly synthesized zeolites, but instead, they are formed as extra-framework or framework-associated defects under the harsh conditions of high-temperature steaming and calcination; procedures used to increase the catalytic activities and hydrothermal stabilities of zeolites prior to industrial use. Evidence of synergistic interaction between the Bro̷nsted and the Lewis acid sites has also been presented.^6^

Direct characterization of the Lewis acid sites is challenging due to their extremely low volumetric abundance (near-surface species) and the high spin of the ^27^Al nucleus (I = 5/2), giving rise to a considerable quadrupolar moment, which, in turn, interacts with the electric field gradient (EFG) to produce potentially very broad and complex line shapes.^7^ Although simple one-dimensional (1D) ^27^Al MAS NMR techniques are routinely used to observe highly symmetric species (e.g., [AlO_4_] or [AlO_6_]), which exhibit relatively narrow line shapes, other species of lower symmetry can result in resonances spanning several MHz (even tens of MHz), making them practically “invisible”.^6^ The development of multiple-quantum (MQ) NMR techniques has recently allowed for direct observation and resolution of some of the low-symmetry species in zeolites.^8−10^ While direct observation of the hypothesized planar [Al(OSi)_3_] remains elusive (predicted *C*_Q_ = 34.8 MHz)^11^ and requires application of strong magnetic fields and ultrawide-line NMR experimental approaches,^7^ its presence in activated zeolites has been inferred through chemisorption of probe ligands (L), most notably pyridine (py), yielding resonances assigned as the corresponding [L–AlO_3_] species.^8^ The small quantity of the species of interest (compared to the total Al content of the materials) hinders more direct investigation of their properties. Therefore, a method of selective preparation of the Lewis acidic Al species in silicate matrices (single-site materials) is sought.

On the synthetic front, Barnes et al. have developed a two-step nonhydrolytic sol–gel procedure for the preparation of microporous metallosilicates under mild conditions.^12−15^ The first step introduces metal centers in a nonhydrolytic condensation of the molecular spherosilicate building block (Me_3_Sn)_8_Si_8_O_20_ (CUBE) with metal precursors MX_n_ (X = halide, alkyl) with the elimination of a Me_3_SnX species. The oligomeric species are cross-linked in the second step with multifunctional chlorosilanes (SiCl_4_, HSiCl_3_, MeSiCl_3_, and Me_2_SiCl_2_). More recently, Styskalik et al.^16^ have used the method under milder conditions, starting from L–AlCl_3_ and [AlCl_4_]^−^, and utilized long, hybrid chlorosilane linkers ClMe_2_Si–(CH_2_)_*n*_–SiMe_2_Cl (*n* = 1–3) to produce single-site Lewis acidic materials with enhanced porosity. Their investigation focused on the catalytic application of the products and did not include any MQ/MAS analysis.

**Figure 1:**
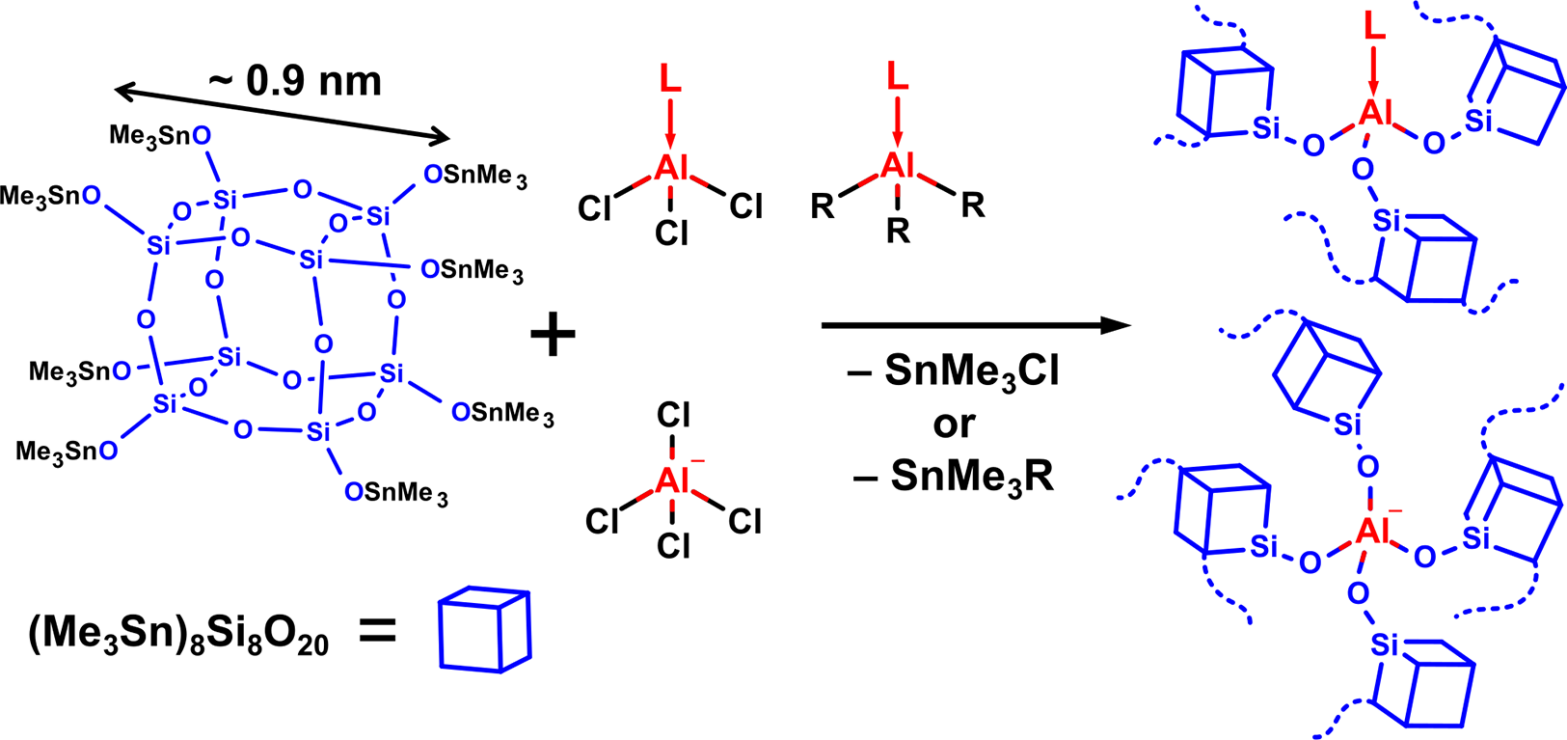
Synthetic strategy leading to statistically linked aluminosilicate oligomers.

In this work, we concentrated on the first step of the synthetic method described by Styskalik et al.^16^ to obtain a series of sparse, amorphous, statistically connected aluminosilicate networks (oligomers) containing [L–AlO_3_] and [AlO_4_]^−^ sites (L = tetrahydrofuran (THF), pyridine (py), triethylamine (Et_3_N), or triethylphosphine oxide (TEPO)). The CUBE building block was reacted with a limited amount of L–AlCl_3_, L–AlMe_3_, L–AlEt_3_, or [Me_4_N] [AlCl_4_] in toluene and THF (Figure 1). The major emphasis of this investigation was placed on the identification of well-defined structural units in these materials and their characterization by ^27^Al TQ/MAS NMR techniques in order to extract their spectroscopic parameters—the isotropic chemical shift (δ_iso_), the quadrupole coupling constant (*C*_Q_), and the asymmetry parameter (η). The chosen palette of ligands provides a range of site symmetries and coordination strengths to correlate their effect on the synthesis and spectroscopic parameters. The impact of the local disorder can be studied in comparisons of materials with two different Al loadings, leading to varying extents of cross-linking and, thereby, different levels of conformational freedom for the sites. The experimentally determined NMR parameters were compared to the values predicted by DFT calculations on the corresponding fully relaxed, truncated models to provide a sound assignment to the experimental spectra and establish a correlation of the *C*_Q_ parameter with the site symmetry. The insight gained from this investigation should provide more clarity to the assignment of ^27^Al solid-state NMR spectra of the more opaque, real-world systems, such as the aforementioned activated zeolites. Finally, a reaction mechanism is proposed for a side-reaction generating [AlO_4_]^−^ sites.

## Experimental Section

### Chemicals and Methods

All procedures were conducted with strict exclusion of oxygen and moisture, using Schlenk techniques with a vacuum-nitrogen manifold. Both precursors and products were stored and manipulated in a drybox (<1 ppm of H_2_O/<1 ppm of O_2_). All glass surfaces of reaction vessels and storage vials were silylated by an equimolar mixture of Me_3_SiCl and Et_3_N in CH_2_Cl_2_ prior to each use to eliminate surface −OH groups and thus prevent the production of HCl in contact with the Al–Cl functionality. The reaction vessels were sealed by PTFE sealing rings. All solvents were dried, deoxygenated, and stored under nitrogen over activated 4 Å molecular sieves. Starting compounds SnMe_3_Cl, AlMe_3_ (2 M in toluene), AlCl_3_, [Me_4_N]OH (25 wt % in MeOH), Si(OEt)_4_, Me_3_SiCl, pyridine, Et_3_N, and TEPO were purchased from Merck and used without further purification. The building block (Me_3_Sn)_8_Si_8_O_20_ was prepared according to a previously described procedure^17^ from SnMe_3_Cl, [Me_4_N]OH, and Si(OEt)_4_. The compounds py–AlMe_3_, Et_3_N–AlMe_3_, TEPO–AlMe_3_, and [Me_4_N] [AlCl_4_] were synthesized according to the procedures described in the Supporting Information (Section S1) and stored in the drybox. The compounds THF–AlCl_3_, py–AlCl_3_, and TEPO–AlCl_3_ were generated *in situ* and never isolated to avoid the formation of complex ionic solids with the potential loss of the ligands.^18,19^

**Caution!***SnMe*_*3*_*Cl, SnMe*_*4*_*, and SnMe*_*3*_*Et are classified as GHS Category 1 acute toxicity (H300, H330, H310) and long-term aquatic toxicity (H400, H410) hazards. The same hazards should be presumed for both (Me*_*3*_*Sn)*_*8*_*Si*_*8*_*O*_*20*_*and the final products (contain residual—SnMe*_*3*_*).*

**Caution!***AlMe*_*3*_*and AlEt*_*3*_*solutions are classified as GHS Category 1 pyrophoric liquids (H250), skin and eye damage (H314, H318), and aspiration (H304) hazards.*

### Synthesis of Aluminosilicate Oligomers

A Schlenk vessel equipped with a stir bar was sealed, evacuated, weighed, taken into the drybox, and loaded with (Me_3_Sn)_8_Si_8_O_20_ (∼2 g, CUBE). Then, the vessel was evacuated and weighed again to determine the exact masses of the vessel and the reagent. The vessel was reconnected to the manifold, the solvent (toluene or THF, 20 cm^3^) was added by a syringe, and the resulting solution was cooled to −80 °C. In the case of compounds L–AlMe_3_ (L = py, Et_3_N, TEPO) and [Me_4_N] [AlCl_4_], a weighed 20 cm^3^ screw-top vial with a PTFE septum was loaded with the corresponding Al complex (1.5 or 3.0 equiv of the reactive groups per CUBE) in the drybox, taken out, and weighed and the solvent (5 cm^3^) was introduced by a syringe to produce a solution. In the case of L–AlCl_3_ (L = THF, py, and TEPO), the ligand and AlCl_3_ were loaded into separate weighed vials and the solvent was first used to dissolve the ligand before it was transferred to the AlCl_3_ vial to generate the complex *in situ*. Care was taken to ensure the ratio of L/Al ≥1 to avoid uncoordinated AlCl_3_. The AlCl_3_ vial was initially cooled by liquid N_2_ to dampen the highly exothermic contact of the ligand with the strong Lewis acid. The room-temperature solution of an Al complex was then added dropwise to the cooled solution of the CUBE with vigorous stirring over 10 min. Another portion of the solvent (5 cm^3^) was used in several doses to ensure a quantitative transfer of L–AlCl_3_. The Schlenk vessel was sealed and allowed to slowly warm up in the cooling bath to room temperature over 12–18 h. Fast warm-ups generally lead to the formation of inhomogeneous precipitates. After 24 h, the solution was heated at 60/100 °C for another 48 h, at which point a clear to hazy solution of oligomers was obtained. All volatiles were removed under dynamic vacuum at 60/100 °C to obtain a glassy residue, which was broken to powder by a magnetic stirrer and outgassed further for 48 h at 60/100 °C. The evacuated Schlenk vessel was then weighed and taken into the drybox for product handling. The solids were analyzed by ^1^H, ^13^C, ^27^Al, ^29^Si, and ^31^P MAS NMR and FTIR spectroscopies, as well as ICP-OES and N_2_ adsorption porosimetry. The volatiles were collected in a Schlenk-type cold trap and analyzed by solution NMR (Supporting Information, Section S2).

The reactions with compounds L–AlCl_3_ and [Me_4_N] [AlCl_4_] could only be conducted in THF due to their low solubility in toluene. Moreover, Et_3_N is incompatible with THF as a solvent due to observed ligand scrambling; therefore, this ligand was used only as Et_3_N–AlMe_3_ and only in toluene.

The mass lost as the volatile byproducts was used to calculate the gravimetric degree of condensation (DC_G_/%) of the reacting functional groups DC_G_(SnMe_3_) and DC_G_(AlX), defining the average connectivity at the CUBE and Al, respectively. Ideally, only a single byproduct would be generated (SnMe_4_, SnMe_3_Et, or SnMe_3_Cl); however additional SnMe_4_ was observed in conjunction with each of the expected byproducts. The excess of SnMe_4_ will be further linked to an unexpected formation of [AlO_4_]^−^ species, and therefore, it is conveniently quantified in mol % with respect to the Al content as DC_G_(SnMe_4_). In the case of compounds L–AlCl_3_, L–AlEt_3_, and [Me_4_N] [AlCl_4_], the byproduct ratio is determined by ^1^H NMR integration while with L–AlMe_3_, a full condensation of Al–Me must be assumed in order to quantify the excess of SnMe_4_. A full description of the calculation procedure and the complete characterization data for all products are provided in the Supporting Information (Sections S2 and S3, respectively).

### Solid-State NMR (ssNMR)

The ^13^C, ^29^Si, and ^31^P MAS ssNMR spectra were acquired in 4 mm ZrO_2_ rotors on a Bruker Avance Neo 700 MHz spectrometer with a MASDVT700S4 BL4 N–P/H probe head at 298 K. ^13^C CP/TOSS^20^ (176.21 MHz) MAS NMR spectra were obtained with 5 kHz MAS frequency, 500 μs contact time, 4 s delays, and ^1^H CW decoupling. All ^13^C ssNMR chemical shifts were referenced externally to adamantane (δ = 38.5 ppm).^21^^29^Si (139.23 MHz) and ^31^P (283.69 MHz) MAS NMR spectra were obtained using the standard 90°-pulse experiment, with 12 kHz MAS frequency, and 60 and 4 s delays for ^29^Si and ^31^P, respectively. The ^29^Si and ^31^P ssNMR chemical shifts were referenced externally to DSS (δ = 1.5 ppm)^21^ and NH_4_H_2_PO_4_ (δ = 1.0 ppm),^22^ respectively. The ^29^Si ssNMR spectra were deconvoluted in SpinWorks 4.2.5 using two peaks (Lorentzian–Gaussian linear combination) centered around −101 (narrow) and −107 (broad) ppm, corresponding to [SnOSi] and [AlOSi] moieties of unreacted and reacted corners of the CUBE, respectively. The ratio of the two peaks was then used to independently calculate the spectroscopic degree of condensation DC_N_ of the tin groups DC_N_(SnMe_3_) for the purpose of validation of the combined gravimetry/^1^H NMR approach. The calculation procedure (Table S1), peak fitting data (Table S18), and the resulting DC values (Table S19) are provided in the Supporting Information.

The ^1^H and ^27^Al MAS ssNMR spectra were acquired in 3.2 mm ZrO_2_ rotors on a Bruker Avance Neo 700 MHz spectrometer with a PH MASDVT700S3 BL3.2 N–P/F-H probe head at 298 K. The samples were packed into the rotors in a drybox, sealed, and stored under the inert atmosphere of the drybox until measurement. The ^1^H spectra were obtained using standard 90°-pulse excitation at 20 kHz MAS frequency with 8 s delays and ^1^H chemical shifts referenced to crystalline alanine (δ = 1.2 ppm, CH_3_ signal). Conventional 1D ^27^Al (182.48 MHz) MAS NMR spectra were obtained using 30°-pulse excitation (1.0 μs) at 20 kHz MAS frequency with 5 s delays and ^1^H SPINAL64 decoupling. 2D ^27^Al TQ/MAS NMR experiments^23^ were set up using kyanite as a model system and carried out at 20 kHz MAS frequency with 2 s delays and ^1^H SPINAL64 decoupling.^24^ The standard *z*-filtered three-pulse sequence with excitation, reconversion, and selective pulse lengths of 4.2, 1.5, and 43.0 μs, respectively, was used. The *z*-filter pulse length was 20 μs. All ^27^Al ssNMR chemical shifts were referenced externally to aqueous [Al(H_2_O)_6_]^3+^ (δ = 0.0 ppm).

The ^27^Al TQ/MAS NMR spectra were processed using the TopSpin 4.1.4 software. Initially, iso-shearing transformation was applied by *xfshear* in order to recover isotropic line shapes in the indirect dimension (F1). Then, individual sites were identified using the maxima of the projections of the spectra into F1 domain and slices were performed along the direct dimension (F2) to obtain 1D spectra of the quadrupolar line shape for each Al site. Finally, with the effects of the chemical shift anisotropy removed by MAS and the pure line shape separated by TQ NMR, the quadrupolar NMR parameters (δ_iso_, *C*_Q_, η) were extracted by quadrupolar line shape simulation fitting using the central transition model. The line broadening parameter (LB) was not optimized but carefully enforced in several steps as it strongly interacts with the *C*_Q_ parameter (both broaden the line shape). Further refinements of the fits were performed based on the assignment of corresponding sites across multiple synthetic products. All TQ/MAS NMR spectra, extracted line shapes, and details of fitting are provided in the Supporting Information (Section S3).

The details of auxiliary characterization techniques (solution NMR, FTIR spectroscopy, ICP-OES, and N_2_ adsorption porosimetry) are available in the Supporting Information (Section S2).

### Computational Methods

The Al sites of interest were modeled by idealized truncated molecular species containing up to four monodentate (OCUBE = (Me_3_Sn)_7_Si_8_O_19_(O−)) or two bidentate (O_2_CUBE = (Me_3_Sn)_6_Si_8_O_18_(O−)_2_, bound through neighboring corners) CUBEs. The terminal −OSnMe_3_ groups of the CUBEs were replaced by −OMe for computational efficiency (fewer nuclei and electrons), and the effect of the substitution was benchmarked. The molecular geometries were optimized by the PBE0 functional^25,26^ and the def2-TZVPP basis set^27^ (the central part of the molecule, e.g., THF–Al(OSiO_3_)_3_, up to and including the first [SiO_4_] tetrahedron) or the def2-SVP basis set^28^ (the rest of the molecule) with the corresponding def2-ECP for Sn atoms^29,30^ as implemented in the Turbomole 7.5.0 program.^31^ The structures were optimized using the DFT-D3^32,33^ dispersion correction with Becke–Johnson damping and the Conductor-like Screening Model (COSMO) solvent model of THF. For the calculations of chemical shielding, the central part of the molecule was taken out (e.g., THF–Al(OSiO_3_)_3_), terminated by hydrogen atoms (e.g., 9 H to form THF–Al(OSi(OH)_3_)_3_) and reoptimized with frozen coordinates of the central atoms. All calculations using this protocol were performed with the *m5* integration grid and the following convergence criteria: 10^–6^ for the density matrix change and 10^–4^ Hartree × Bohr^–1^ for the geometry gradient.

The calculation of ^27^Al NMR parameters was performed using the zeroth-order regular approximation (ZORA)^34,35^ Hamiltonian at the scalar (spin-free) level, as implemented in the ADF program package (version 2019).^36^ The *C*_Q_ and η parameters (the whole molecule was used) were calculated by the PBE0 functional, the TZ2P basis set (the QZ4P basis set for the Al atom),^37,38^ and the COSMO solvent model of THF. The chemical shielding (the central part of the molecule was used) was calculated using the PBE0 functional, the QZ4P basis set, and the COSMO (THF). The calculated chemical shifts were referenced relative to the experimental chemical shift of solid AlCl_3_ as a secondary reference (δ_ref_ = −1.6 ppm),^7^ and they are reported relative to AlCl_3_ in D_2_O (primary reference). The Euler angles (α, β, γ) were extracted from ADF output files using the program *INFOR*.^39^

The full list of the calculated spectroscopic parameters is available in the Supporting Information (Section S4).

## Results and Discussion

The first step of the nonhydrolytic sol–gel procedure of Styskalik et al.,^16^ as described in the experimental details, was used with the aim to produce single-site materials containing exclusively L–Al(OCUBE)_3_ (L = THF, Et_3_N, py, TEPO) or [Me_4_N]^+^ [Al(OCUBE)_4_]^−^ sites. The molecular spherosilicate building block CUBE was cross-linked by limited amounts of L–AlCl_3_, L–AlMe_3_, L–AlEt_3_, or [Me_4_N] [AlCl_4_], affording sparse, statistically linked networks–oligomers (Table 1). Reactions **1**–**5** and **7**–**16** produced nonporous glassy solids that could be reversibly redissolved into colloidal viscous solutions, indicating the oligomeric character of the formed species. Reaction **6** produced a gel upon heating and subsequently an insoluble porous (126 m^2^ g^–1^) solid upon drying, indicating an even higher extent of cross-linking leading to the formation of an extended 3D network. In the following discussion, the same numbering system refers to both reactions and the resulting materials (products).

**Table 1:** Synthesis and Important Characterization Data for Prepared Oligomers

		**stoichiometry**					**average connectivity**	
**sample**	**Al precursor**	**Al/CUBE**	**AlX/CUBE**	**solvent/temperature (°C)**	**primary condensation** DC_G_**(Al–X)(%)**	**excess SnMe**_**4**_DC_G_**(SnMe**_**4**_**)** **(mol % of Al)**	**Al**	**CUBE**
**1**	THF–AlCl_3_	0.99	2.97	THF/60	105.1	27.6	3.28	3.39
**2**	0.5 py–AlCl_3_	0.48	1.45	THF/60	100.0	56.2	3.56	1.72
**3**	py–AlCl_3_	0.97	2.90	THF/60	103.1	7.1	3.07	3.06
**4**	0.5 py–AlMe_3_	0.49	1.47	TOL/60	100.0	31.8	3.32	1.63
**5**	py–AlMe_3_	1.01	3.03	TOL/60	100.0	6.9	3.07	3.10
**6**^a^	py–AlMe_3_	0.99	2.96	**TOL/100**	100.0	58.9	3.59	3.54
**7**	0.5 py–AlMe_3_	0.49	1.46	THF/60	100.0	62.2	3.62	1.76
**8**	py–AlMe_3_	0.99	2.97	THF/60	100.0	11.5	3.12	3.08
**9**	py–AlEt_3_	1.00	3.00	TOL/60	**96.7**	6.5	2.97	2.97
**10**	0.5 Et_3_N–AlMe_3_	0.50	1.51	TOL/60	100.0	16.6	3.17	1.60
**11**	Et_3_N–AlMe_3_	1.00	3.00	TOL/60	100.0	7.4	3.07	3.07
**12**	0.5 TEPO–AlCl_3_	0.50	1.49	THF/60	100.4	0.0	3.00	1.49
**13**	TEPO–AlCl_3_	0.99	2.97	THF/60	**96.1**	1.7	2.85	2.82
**14**	0.5 TEPO–AlMe_3_	0.50	1.50	TOL/60	100.0	0.5	3.01	1.50
**15**	TEPO–AlMe_3_	0.98	2.95	**TOL/60**^b^	**83.9**	0.0	2.47	2.47
**16**	0.375 [Me_4_N][AlCl_4_]	0.37	1.49	THF/60	**89.0**	41.9	3.98	1.48

a Porous: SA_BET_ = 126 m^2^ g^–1^.

b Heated in solution for 72 h and dried under vacuum for 120 h in attempts to achieve full condensation.

The condensation reaction should ideally produce only a single volatile byproduct SnMe_3_X (X = Cl, Me, or Et), corresponding to the type of reactive functional groups in the particular L–AlX_3_ precursor. The analysis of the volatiles removed from the reactions, however, revealed that additional SnMe_4_ was generated even in the case of precursors containing the Al–Et and Al–Cl functional groups, leading to SnMe_3_Et/SnMe_4_ and SnMe_3_Cl/SnMe_4_ mixtures, respectively. Conveniently, no evidence of mutual interaction or substituent exchange was observed and the two byproducts could be distinguished and their molar ratio determined by ^1^H NMR integration, allowing for a combined gravimetry/NMR quantification technique. The resulting values of the gravimetric degree of condensation of the −SnMe_3_ groups (DC_G_(SnMe_3_)) were compared to their spectroscopic counterparts calculated from ^29^Si MAS NMR integration (DC_N_(SnMe_3_)) and elemental analysis obtained from ICP-OES(Sn)/gravimetry (DC_E_(SnMe_3_)) (Table S19) to make sure the quantification procedure was accurate. The resulting correlation plot (Figure S33) shows generally good agreement among all three sources of information.

Of note is the observation that no free ligand (L) was detected in the volatile byproducts, with the exception of reactions **1**–**3** where the ligands and AlCl_3_ were supplied separately and deliberately with a slight excess of the ligand. This indicates that L/Al = 1 is maintained.

In the case of precursors L–AlX_3_ (X = Me, Et, Cl) (reactions **1**–**15**), the gravimetric analysis revealed that full condensation of the AlX groups DC_G_(AlX) is generally reached, with the exception of reactions **9**, **13**, and **15**. The small deviations of DC_G_ in reactions **1**, **3**, **9**, **12**, and **13** (within 5.1%) could easily be attributed to experimental error, while the deviation of **15** (−16.1%) is a strong proof of incomplete condensation.

The quantification of the excess of SnMe_4_ showed the generated amount to be significant and highly variable, yet always smaller than the quantity of Al. Since a full condensation was nearly always reached, a separate process involving the Al site and the consumption of another −SnMe_3_ group must be at play. With no reasonable redox options, the transformation of [L–AlO_3_] into [AlO_4_]^−^ in a metathesis reaction, liberating SnMe_4_ at the expense of the introduction of additional connectivity, was suspected. Therefore, the amount of SnMe_4_ was expressed as a fraction of the quantity of the Al sites DC_G_(SnMe_4_). The comparison of reactions **1**, **3**, **5**, **8**, **9**, **11**, **13**, and **15** indicates that the quantity of SnMe_4_ depends strongly on the ligand used in the series THF > Et_3_N ≥ py > TEPO ≈ 0 while it is independent of the substituent X. Further comparison of the reactions **3**, **5**, **8**, and **11** (AlX/CUBE = 3.0) to their less extensively cross-linked counterparts **2**, **4**, **7**, and **10** (AlX/CUBE = 1.5) clearly demonstrates the suppression of the reaction in samples with more cross-linking. This is attributed to the loss of conformational freedom. Moreover, reactions **4** and **5** (L = py) compared to **10** and **11** (L = Et_3_N) indicate that with the lower extent of cross-linking, the sterically more demanding Et_3_N also becomes a limiting factor. Similarly, evidence that more SnMe_4_ is generated in THF could be found in the contrast between reactions **2** and **7** (in THF) and reaction **4** (in toluene). Finally, reaction **6**, which was conducted at an increased temperature (100 °C), produced nearly ten times as much SnMe_4_ as the corresponding reaction at 60 °C (**5**), leading to the highest overall average connectivity (3.59 on Al/3.54 on CUBE) at the formation of a porous gel structure (BET 126 m^2^ g^–1^).

In the case of [Me_4_N] [AlCl_4_], only the AlX/CUBE = 1.5 stoichiometry was used to avoid too heavily cross-linked products. To our surprise, a full condensation, as monitored by the evolved SnMe_3_Cl, was not reached and SnMe_4_ was also generated. Further analysis revealed that the total average connectivity on Al reached 4 (3.98), pointing at the existence of a competing mechanism of condensation where the Cl atom remains embedded in the material and SnMe_4_ is evolved instead.

### Basic Characterization

**Figure 2:**
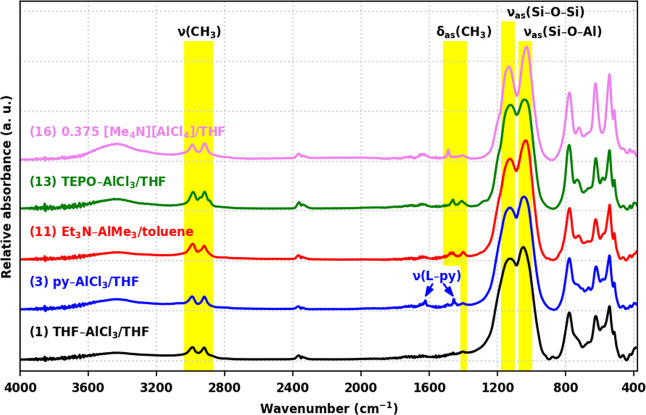
FTIR spectra (KBr pellets) of products **1**, **3**, **11**, **13**, and **16**, demonstrating the general similarity of the products, with differences in the presence of the vibration bands of the individual ligands.

The infrared spectra of dried products (Figure 2) are dominated by the strong vibration bands at 1139 and 1035 cm^–1^ assigned to the intracage ν_as_(Si–O–Si) and the extra-cage ν_as_(Si–O–Al) linkages of the CUBE, respectively.^40,41^ The vibration bands at 1402, 2919, and 2990 cm^–1^ are also common to all the prepared samples and primarily assigned to the bending and valence vibrations δ_as_(CH_3_), ν_s_(CH_3_), and ν_as_(CH_3_) of the residual −SnMe_3_ groups.^42^ Additional bands, corresponding to the valence/bending vibrations of the ethyl substituents of Et_3_N (1470 cm^–1^)^43^ and TEPO (1460, 2890, and 2942 cm^–1^), as well as the δ_as_(CH_3_) vibrations of [Me_4_N]^+^ (1488 cm^–1^),^44^ are observable in the spectra of the corresponding products. The spectra of products prepared from py–AlX_3_ (X = Me, Et, Cl) (Figure S34) are identical and all contain vibration bands at 1455, 1495, and 1622 cm^–1^ assigned to Lewis acid-bound pyridine (marked L-py in Figures),^45,46^ indicating a single, well-defined mode of ligand coordination and the convergent nature of the synthesis from different precursors. In general, the fingerprint area is nearly identical across all ligands/precursors.

**Figure 3:**
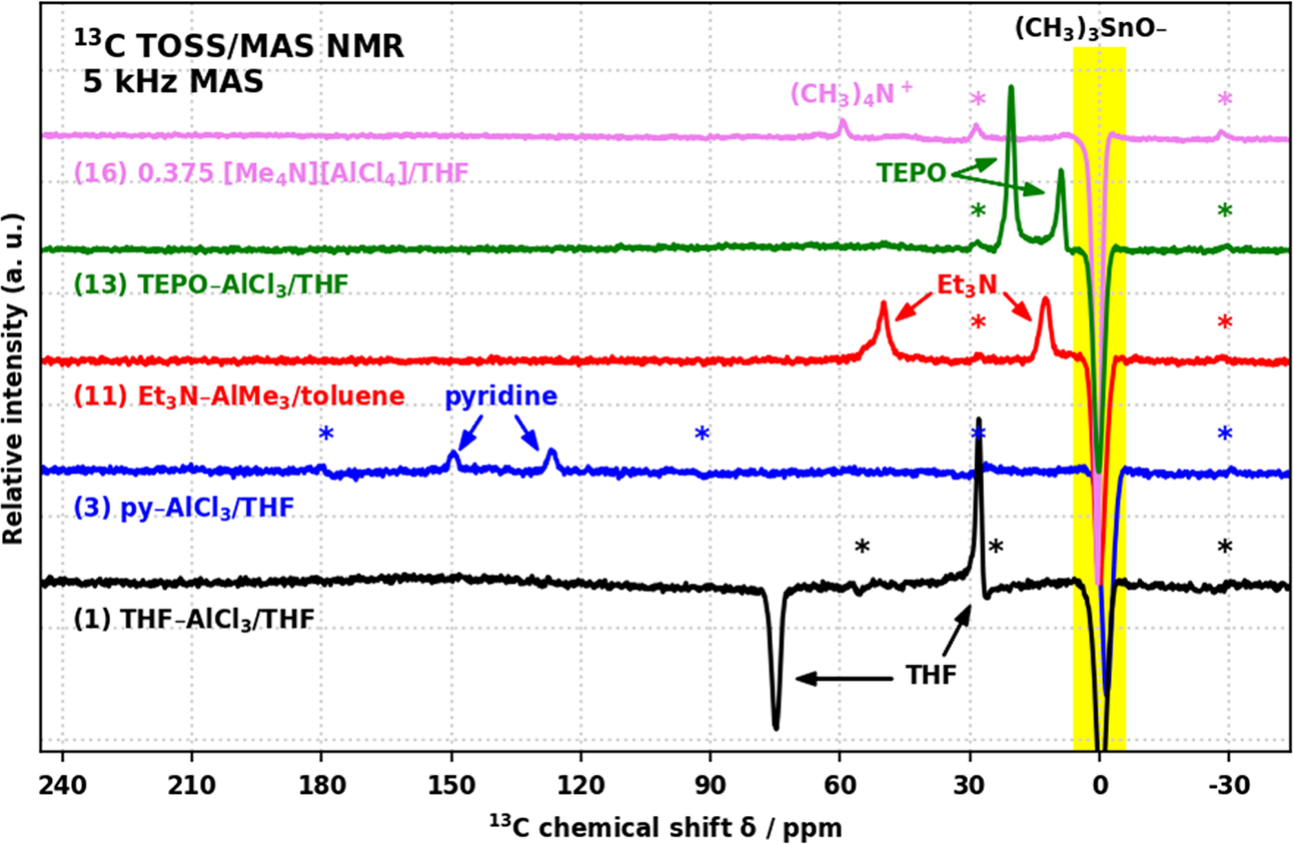
^13^C CP/TOSS MAS NMR spectra of products **1**, **3**, **11**, **13**, and **16**. Spinning side-bands are denoted by asterisks.

^13^C CP/TOSS MAS NMR spectra of all products (Figure 3) contain a prominent resonance from −1 to 1 ppm, assigned to the residual −SnMe_3_ groups, with additional resonances corresponding to the particular ligand/cation used: THF (28 and 75 ppm), Et_3_N (13 and 50 ppm), py (129 and 151 ppm), TEPO (10 and 21 ppm), and [(*C*H_3_)_4_N]^+^ (59.3 ppm).^47^ In parallel to the FTIR spectra, the ^13^C CP/TOSS MAS NMR spectra (Figure S35) of products prepared from py–AlX_3_ (X = Me, Et, Cl) are identical. The comparison of ^13^C ssNMR spectra of products **12**–**15** (Figure S36) reveals an additional resonance in the spectrum of **15** at −10 ppm, which could be assigned to residual Al–*C*H_3_ due to incomplete condensation as indicated by gravimetry. The negative amplitude of the −SnMe_3_ resonances is indicative of their large chemical shift anisotropy with respect to the MAS frequency.^48,49^

^1^H MAS NMR characterization (Figures S37 and S38) completely mirrors the ^13^C ssNMR spectra with a common resonance at ∼0 ppm, assigned to the residual −SnMe_3_, and individual resonances for the corresponding bound ligands and the cation: THF (1.3 and 3.6 ppm), Et_3_N (1.1 and 2.8 ppm), py (7.5 and 8.8 ppm), TEPO (1.1 and 1.9 ppm), and [(C*H*_3_)_4_N]^+^ (3.0 ppm).^50^ In the case of product **15**, the expected resonance of the residual Al–C*H*_3_ is observed at −1.3 ppm (Figure S39).

**Figure 4:**
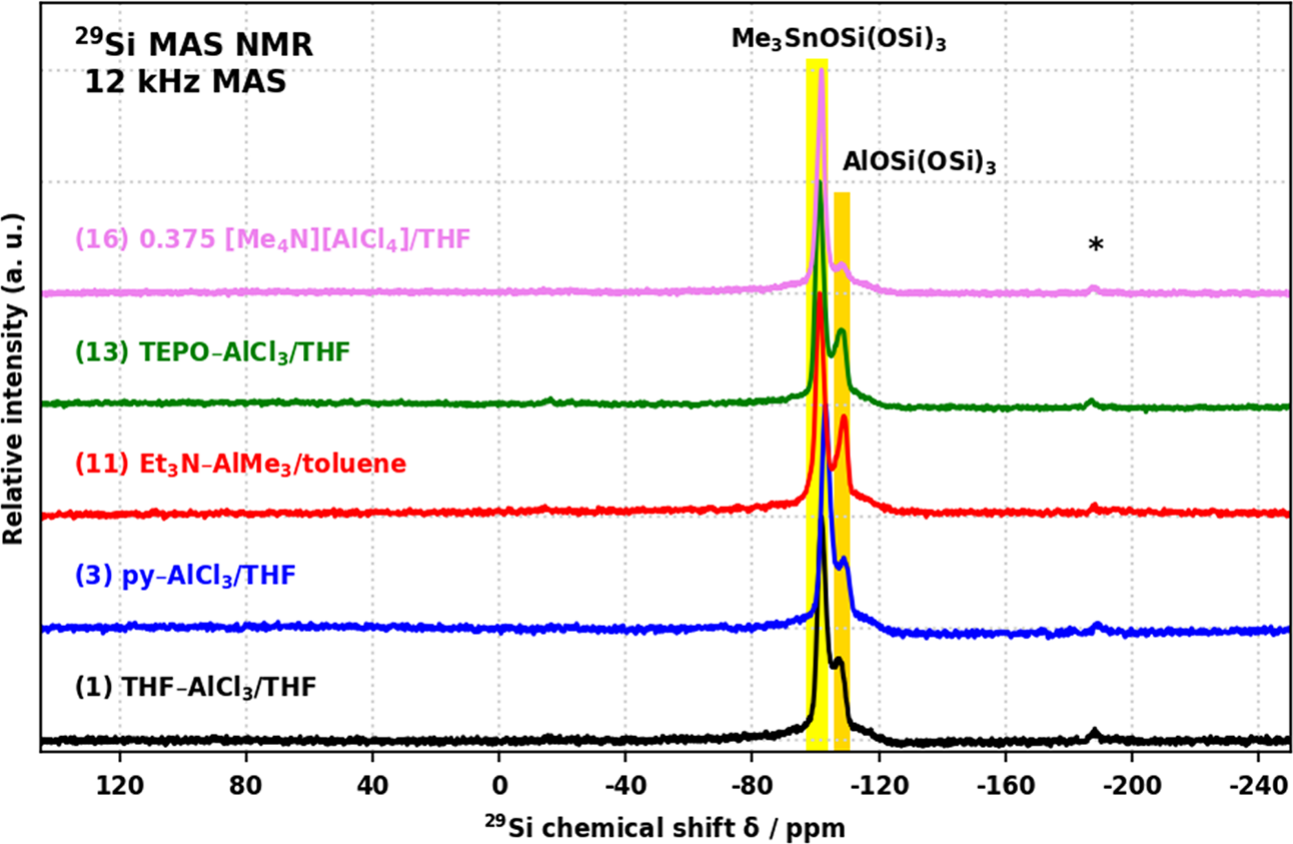
^29^Si MAS NMR spectra of products **1**, **3**, **11**, **13**, and **16**. Spinning side-bands are denoted by asterisks.

^29^Si MAS NMR spectra of all samples contain only two [SiO_4_] resonances at −101 ppm (narrow) and −107 ppm (broad), assigned to Me_3_SnO*Si*(O−)_3_ and AlO*Si*(O−)_3_, respectively (Figure 4).^14,51^ The intensity ratio of the two resonances reflects the reaction stoichiometry and the degree of condensation, DC (Figure S33).

**Figure 5:**
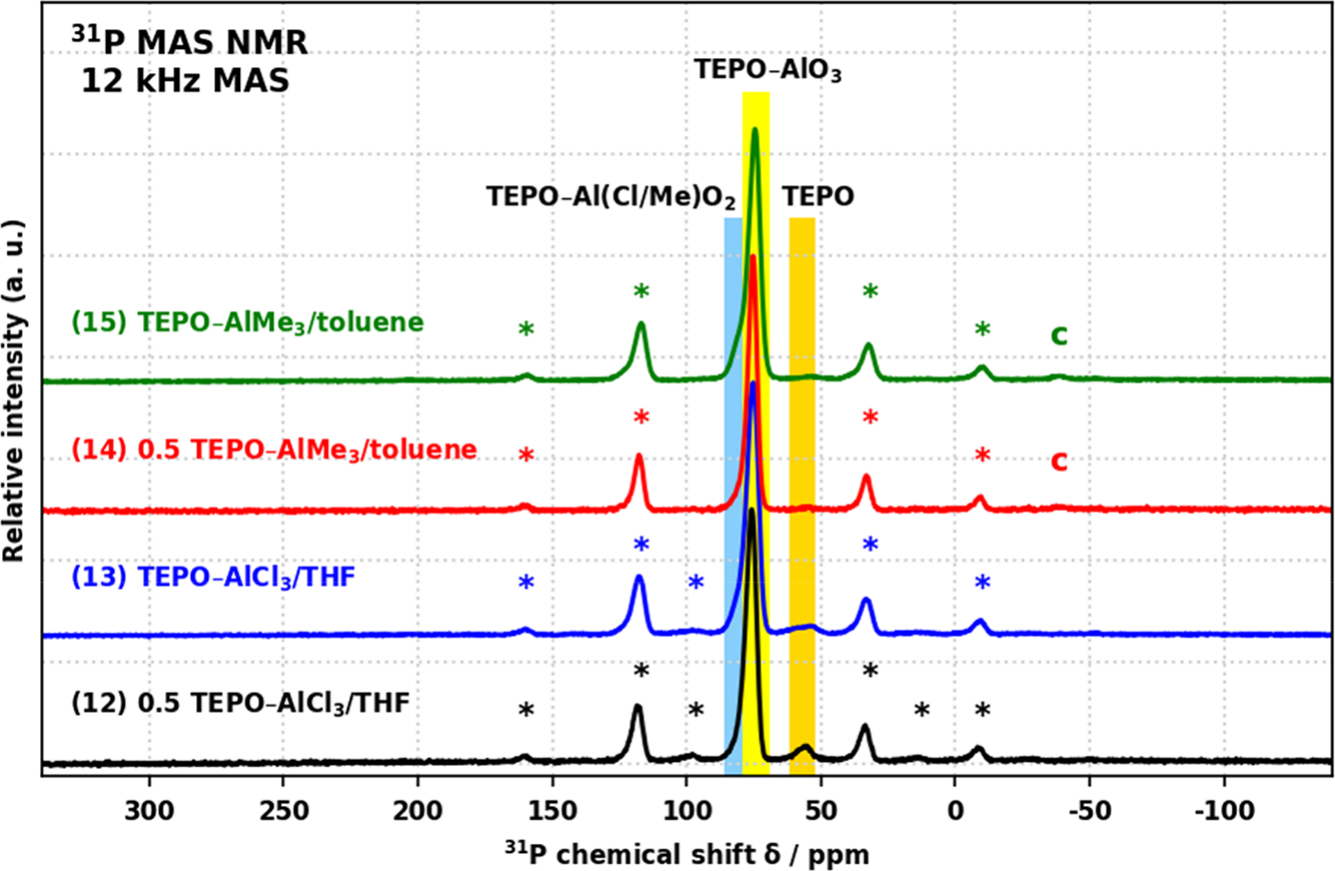
^31^P MAS NMR spectra of products **12**–**15**. Spinning side-bands are denoted by asterisks; a trace rotor contaminant is denoted by c.

^31^P MAS NMR spectra of products **12**–**15** (Figure 5) are dominated by a resonance at 75 ppm, assigned to the intended TEPO–Al(O−)_3_ site. In the spectra of products **12** and **13**, a minor resonance at 55 ppm was assigned to uncoordinated TEPO.^52^ Additionally, the spectra of **13** and **15** contain low-intensity shoulders at 81 ppm, for the latter the shoulder is more pronounced. Based on their similarity to the main site and the incomplete condensation indicated by gravimetry, these are assigned as TEPO–AlCl(O−)_2_ and TEPO–AlMe(O−)_2_, respectively.

### 1D ^27^Al MAS NMR Analysis

**Figure 6:**
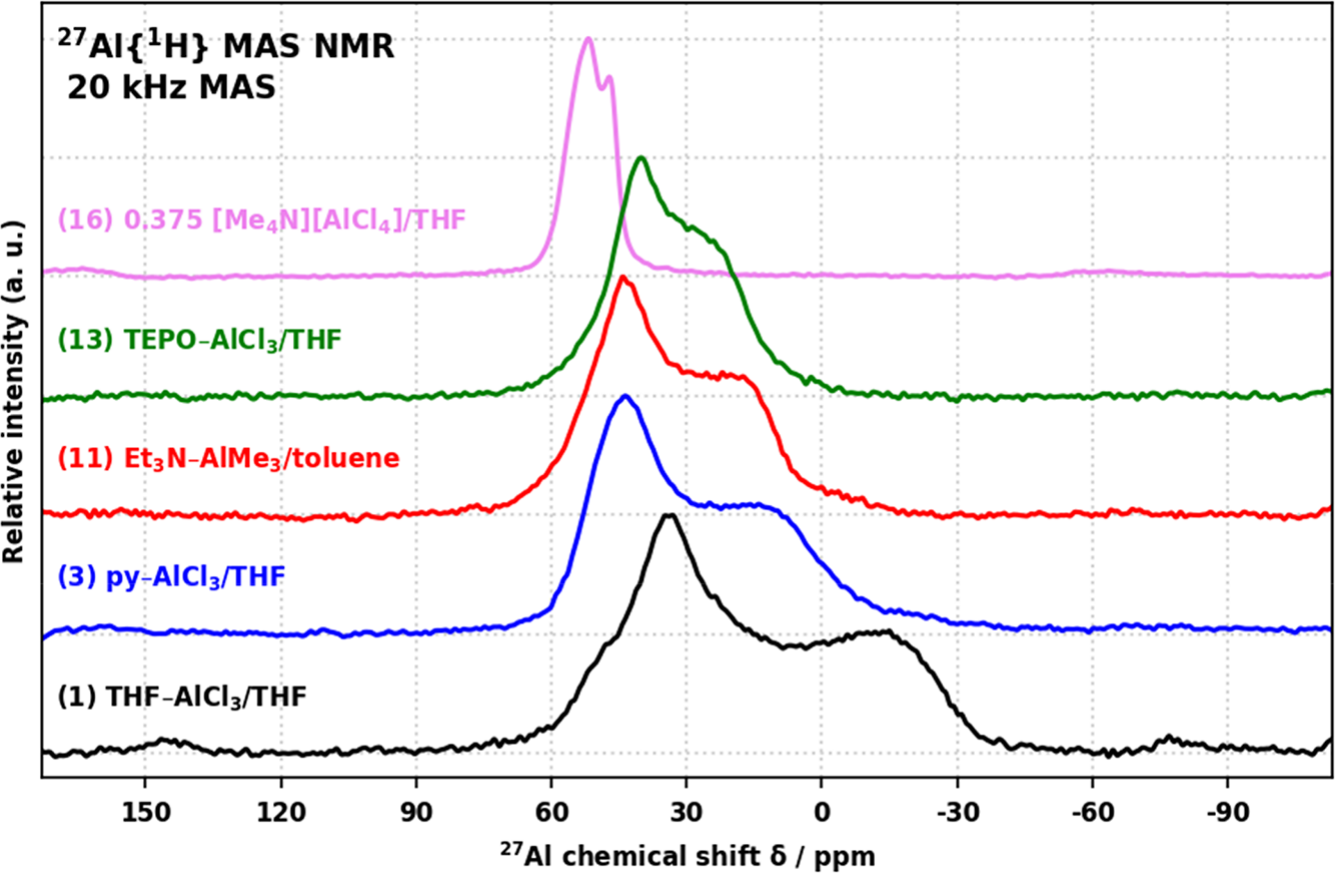
^27^Al {^1^H} MAS NMR spectra of products **1**, **3**, **11**, **13**, and **16**.

^27^Al{^1^H} MAS NMR characterizations (Figure 6) of products **1**–**15** showed broad asymmetric features with increasing line width in the order TEPO < Et_3_N < py < THF. Single quadrupolar nucleus line shapes did not yield good fits to the observed features, hinting at the presence of multiple overlapped resonances and preventing direct extraction of the spectroscopic parameters. Product **16** showed a much narrower feature with two maxima at 47 and 52 ppm, allowing for a confident assignment as four-coordinated Al species.^53,54^ Most notably, the observed line shapes are more complex (**16**) and much broader (**1**–**15**) than those previously reported^16^ for the corresponding products. This is attributed to the broader-band excitation used in this work and demonstrates the trickiness involved in the characterization of low-symmetry Al species by ^27^Al NMR techniques.

### Multidimensional ^27^Al MAS NMR Analysis

**Figure 7:**
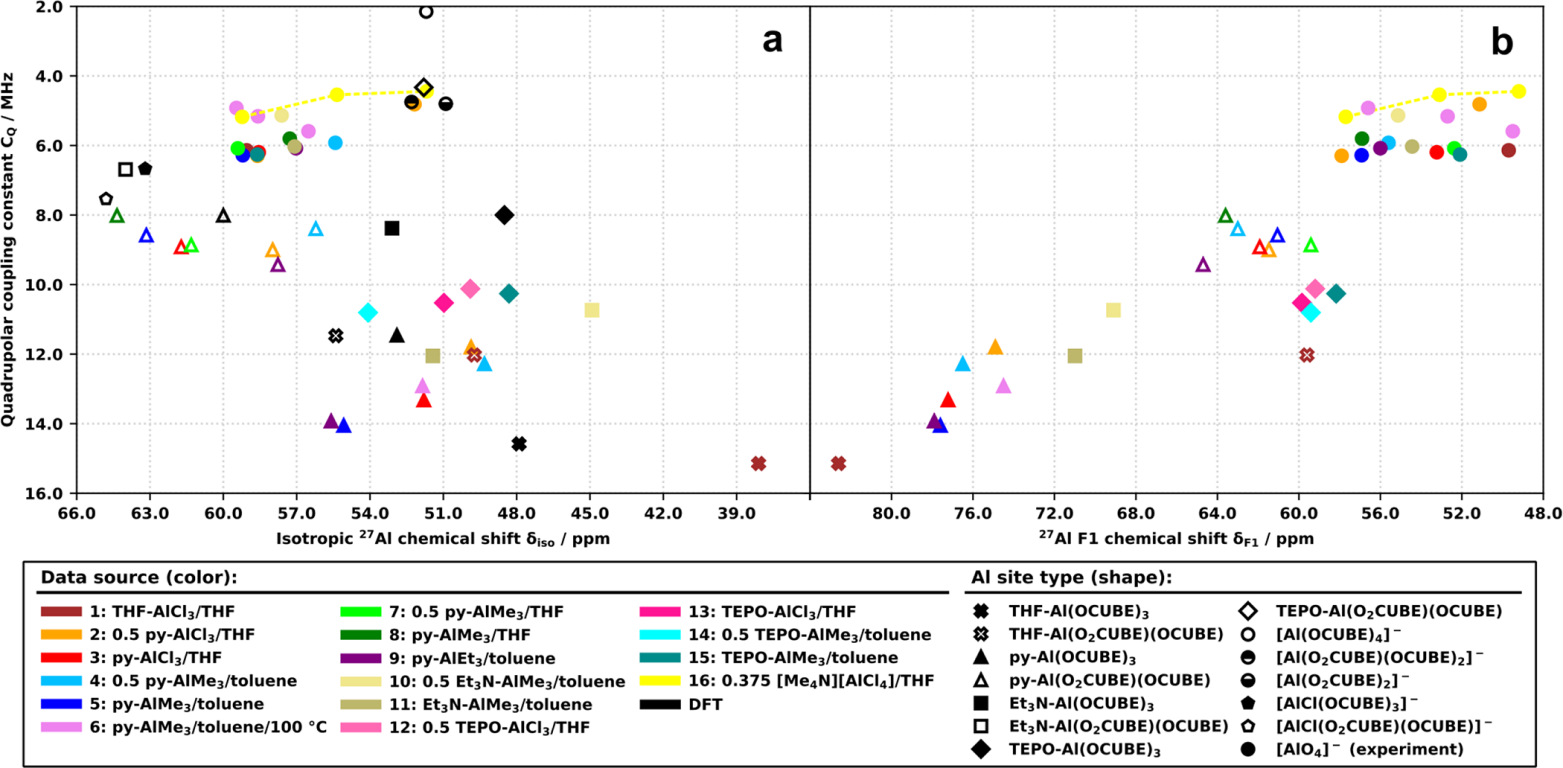
Plots of all observed ^27^Al TQ/MAS NMR resonances and relevant DFT-calculated models in parameter spaces (a) δ_iso_/*C*_Q_ and (b) δ_F1_/*C*_Q_.

In contrast, the 2D ^27^Al TQ/MAS NMR spectra revealed a more complex nature of the prepared materials. The resonance assignments presented below were made based on the clustering (grouping) of the fitted resonances in parameter space (δ_F1_, δ_iso_, *C*_Q_) across all the prepared products (Figure 7) and the proximity of the clusters to the values predicted by DFT calculations (CUBEs terminated by −OMe). Further refinements of the fits were made once the initial grouping was established. The full list of all the observed resonances (labeled by product number and a letter, e.g., 1/A) and their ^27^Al NMR parameters and assignments are available in Table S20. Tables S21 and S22 summarize all the model structures investigated by DFT calculations and the predicted parameters. *C*_Q_ is the most reliably fitted parameter as it depends primarily on the limits of the peak envelope, while η is the least reliable as it subtly changes the peak shape within the limits of the envelope. The fit of δ_iso_ is contaminated by the uncertainty of η. The less chemically relevant indirect chemical shift δ_F1_ can be measured directly with the greatest precision from the 1D projections of the ^27^Al TQ/MAS spectra. Therefore, the assignments were made using plots of δ_iso_/*C*_Q_ (Figure 7a) and δ_F1_/*C*_Q_ (Figure 7b), with the latter showing more distinct clustering and serving as the starting point for the assignment. Figure S40 shows the lack of significant clustering in the η/*C*_Q_ space, demonstrating the unreliability of the fitted η values.

**Figure 8:**
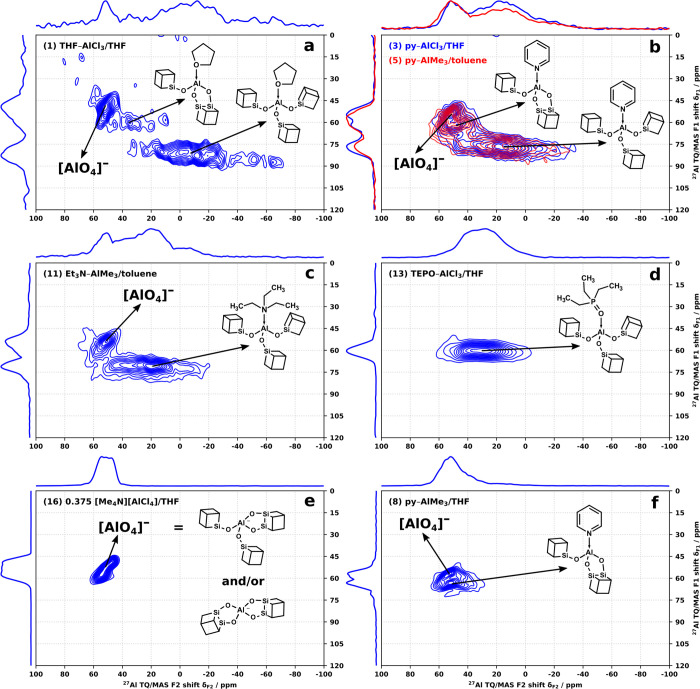
^27^Al TQ/MAS NMR spectra of (a) product **1**, (b) products **3** and **5**, (c) product **11**, (d) product **13**, (e) product **16**, and (f) product **8** (20 kHz MAS).

Figure 8 then represents a portfolio of all the observed ^27^Al TQ/MAS NMR resonances in fully condensed products. All products **1**–**15** displayed broad resonances (*C*_Q_ = THF: 15.1, py: 11.8–14.1, Et_3_N: 10.7–12.1, and TEPO: 10.1–10.8 MHz) corresponding to the sought L–Al(OCUBE)_3_ sites; however, only in the case of **12**–**14** (L = TEPO) were they the only sites present (true single-site materials).

In products **1**–**9** (L = THF, py), another series of narrow resonances (*C*_Q_ = THF: 12.0, py: 8.0–9.4 MHz), assigned as L–Al(O_2_CUBE)(OCUBE), was also identified, indicating that a bidentate CUBE binding mode is possible with the two sterically relatively undemanding ligands. The site is visibly more dominant in products **7** and **8** (py–AlMe_3_/THF) (Figure 8f), than in **2**, **3** (py–AlCl_3_/THF), **4**, and **5** (py–AlMe_3_/toluene), while it is significantly suppressed in **9** (py–AlEt_3_/toluene). This could suggest that py–AlMe_3_ is sensitive to solvent effects with THF promoting cyclization more than toluene and that the substitution of Al–Me for larger alkyls tends to disfavor the process.

As suspected based on gravimetry, the spectra of products **1**–**12** displayed additional narrow resonance(s) (*C*_Q_ = 4.8–6.3 MHz), assigned to [AlO_4_]^−^ species. The dominance of these signals in the spectra appears to correlate with the amount of excess SnMe_4_ generated. Similarly, product **16**, which was intended to produce solely [AlO_4_]^−^, displays only a single band of narrow resonances (*C*_Q_ = 4.4–5.2 MHz) with three discernible maxima of increasing δ_iso_ and *C*_Q_ (Figure 8e). The bands of [AlO_4_]^−^ resonances of **1**–**12** possess the same oblique characteristic and generally overlap with the one of **16**, allowing for fitting of 3 or fewer maxima.

The product **15** (TEPO–AlMe_3_/toluene) is a special case where full condensation could not be achieved despite increased reaction and drying times (72 and 120 h). The ^27^Al TQ/MAS spectrum (Figure S41) revealed the presence of a weak broad resonance (*C*_Q_ = 12.8 MHz) assigned to TEPO–AlMe(OCUBE)_2_, but also a significant resonance of [AlO_4_]^−^, indicating that the increased heating times did not overcome the barrier to condensation but rather promoted the formation of [AlO_4_]^−^.

In general, the resonances observed in the spectra of the products with decreased Al loading (**2**, **4**, **7**, **10**, **12**, and **14**) were systematically narrower (giving lower fitted values of *C*_Q_) than the corresponding ones in the higher-loading products (**3**, **5**, **8**, **11**, **13**, and **15**), at the expense of a decreased signal-to-noise ratio. This is interpreted primarily as the result of the increased structural disorder in the more heavily cross-linked materials. Notably, a trend can be found in the *C*_Q_ values of py–Al(OCUBE)_3_ with a systematic decrease in the series: **5** ≈ **9** (py–AlX_3_/toluene, X = Me, Et) > **3** (py–AlCl_3_/THF) > **6** (py–AlMe_3_/toluene/100 °C) > **4** (0.5 py–AlMe_3_/toluene) > **2** (0.5 py–AlCl_3_/THF). This may be interpreted as the substitution of Al–Cl precursors for Al–Me/Al–Et and thermal relaxation (**6**) also act to decrease the site disorder. Resonances **1/A** (THF–Al(OCUBE)_3_) and **15/C** (TEPO–AlMe(OCUBE)_2_) suffer from uneven excitation due to their extremely large *C*_Q_ (≈ 15 MHz), resulting in very poor fits.

Table 2 summarizes the average spectroscopic properties of all the observed types of sites, along with the corresponding values predicted by the DFT calculations (−OMe-terminated CUBEs). Since the predictions come from fully relaxed model structures of particular, single conformers, the values of δ_iso_^DFT^ and η^DFT^ are subject to slight variations and the predicted |*C*_Q_^DFT^| must be understood as the lower limits in comparisons to the experimental data; thus, it is best related to the minimum observed values *C*_Q_^min^. The average isotropic chemical shifts δ_iso_^avg^ of all the experimentally observed, well-excited resonances as well as all the corresponding DFT predictions δ_iso_^DFT^ fall within the range of 48–60 ppm, establishing a good agreement with the chemical shifts of 4-coordinate Al species reported previously in the literature.^53,54^ Most importantly, the observed *C*_Q_^min^ values for the sites L–Al(OCUBE)_3_ match remarkably well to the predicted |*C*_Q_^DFT^|, with both data sets following the key trend of a decreasing *C*_Q_ with L in the series THF > py > Et_3_N > TEPO, clearly reflecting the increasing symmetry of the coordinated L in the series THF (C_1_) < py (C_2_) < Et_3_N (close C_3_) ≤ TEPO (distant C_3_). The series may be expanded with the DFT predictions for the unobserved sites [AlCl(OCUBE)_3_]^−^ (L = Cl^–^, point-like) and [Al(OCUBE)_4_]^−^ (true *T*_d_ at Al) to span the range of *C*_Q_ = 2–15 MHz, pointing to the ligand as the dominant symmetry-breaking element and its connection to the largest component *V*_33_ of the EFG tensor. In the case of py–Al(OCUBE)_3_, the observed *C*_Q_^min^ = 11.8 MHz maps very well to the previously reported value of 9.4 ± 0.5 MHz for the analogous sites observed after the adsorption of pyridine onto dehydrated zeolites.^8^ Similarly, the observed *C*_Q_^min^ = 10.1 MHz for TEPO–Al(OCUBE)_3_ and 4.8 MHz for [AlO_4_]^−^ compare well to the reported values of 11.3 and 4.6 MHz, assigned to highly distorted sites of the corresponding type in synthetic amorphous aluminosilicate materials after the adsorption of TEPO.^55^ The larger positive deviations of *C*_Q_^min^ compared to |*C*_Q_^DFT^| for L = Et_3_N (2.3 MHz) and TEPO (2.1 MHz) could be explained as an additional disorder due to the conformational freedom of the pendant ethyl substituents of the two ligands. As illustrated above, the experimental fits of the asymmetry parameter η^avg^ for the L–Al(OCUBE)_3_ sites are highly unreliable with values generally larger or comparable to the corresponding predicted η^DFT^. In agreement with the predictions, the sites L–Al(O_2_CUBE)(OCUBE) (L = THF, py) exhibited systematically higher δ_iso_^avg^ and lower *C*_Q_^min^ than the corresponding L–Al(OCUBE)_3_ sites. Although the site TEPO–AlMe(OCUBE)_2_ yields poor experimental fits, the data supports the assignment with a predicted shift toward larger δ_iso_ and *C*_Q_, as compared to TEPO–Al(OCUBE)_3_.

**Table 2:** Overview of the Extracted Spectroscopic Parameters of Observed Sites and the Corresponding Values Predicted by DFT Calculations

**site type**	**source**	**δ**_**iso**_**(ppm)**	***C*_Q_^avg^**^a^**(MHz)**	***C*_Q_^min^(MHz)**	**η**
**TH**F–Al(**OCUBE)**_**3**_	exp	38.1	15.1	15.1	0.683
	DFT	47.9	14.6		0.244
**py–Al(OCUBE)**_**3**_	exp	52.3	13.0	11.8	0.682
	DFT	52.9	11.5		0.334
**Et**_**3**_N–Al(**OCUBE)**_**3**_	exp	48.2	11.4	10.7	0.848
	DFT	53.1	8.4		0.226
**TEP**O–Al(**OCUBE)**_**3**_	exp	50.8	10.4	10.1	0.431
	DFT	48.5	8.0		0.453
**TH**F–Al(**O**_**2**_**CUBE)(OCUBE)**	exp	49.7	12.0	12.0	0.100
	DFT	55.4	11.5		0.950
**py–Al(O**_**2**_**CUBE)(OCUBE)**	exp	60.4	8.7	8.0	0.510
	DFT	60.0	8.0		0.974
**TEP**O–Al**Me(OCUBE)**_**2**_	exp	66.9	12.8	12.8	0.439
	DFT	84.5	14.9		0.614
**[AlO**_**4**_**]**^**–**^	exp: 1–15	57.6	5.8	4.8	0.577
	exp: 16/A	51.7	4.4		0.477
	exp: 16/B	55.3	4.5		0.508
	exp: 16/C	59.2	5.2		0.504
**[Al(OCUBE)**_**4**_**]**^**–**^	DFT	51.7	2.2		0.692
**[Al(O**_**2**_**CUBE)(OCUBE)**_**2**_**]**^**–**^	DFT	52.3	4.8		0.394
**[Al(O**_**2**_**CUBE)**_**2**_^**–**^**]**^–^	DFT	50.9	4.8		0.018
**[Me**_**4**_**N]**^**+**^**[Al(OCUBE)**_**4**_**]**^–^	DFT	50.7	2.0		0.884
**[AlCl(OCUBE)**_**3**_**]**^**–**^	DFT	63.2	6.7		0.535
**[AlCl(O**_**2**_**CUBE)(OCUBE)]**^**–**^	DFT	64.8	7.5		0.902

a *C*_Q_ data obtained by DFT are taken as absolute values **|***C*_**Q**_^**DFT**^**|**.

**Figure 9:**
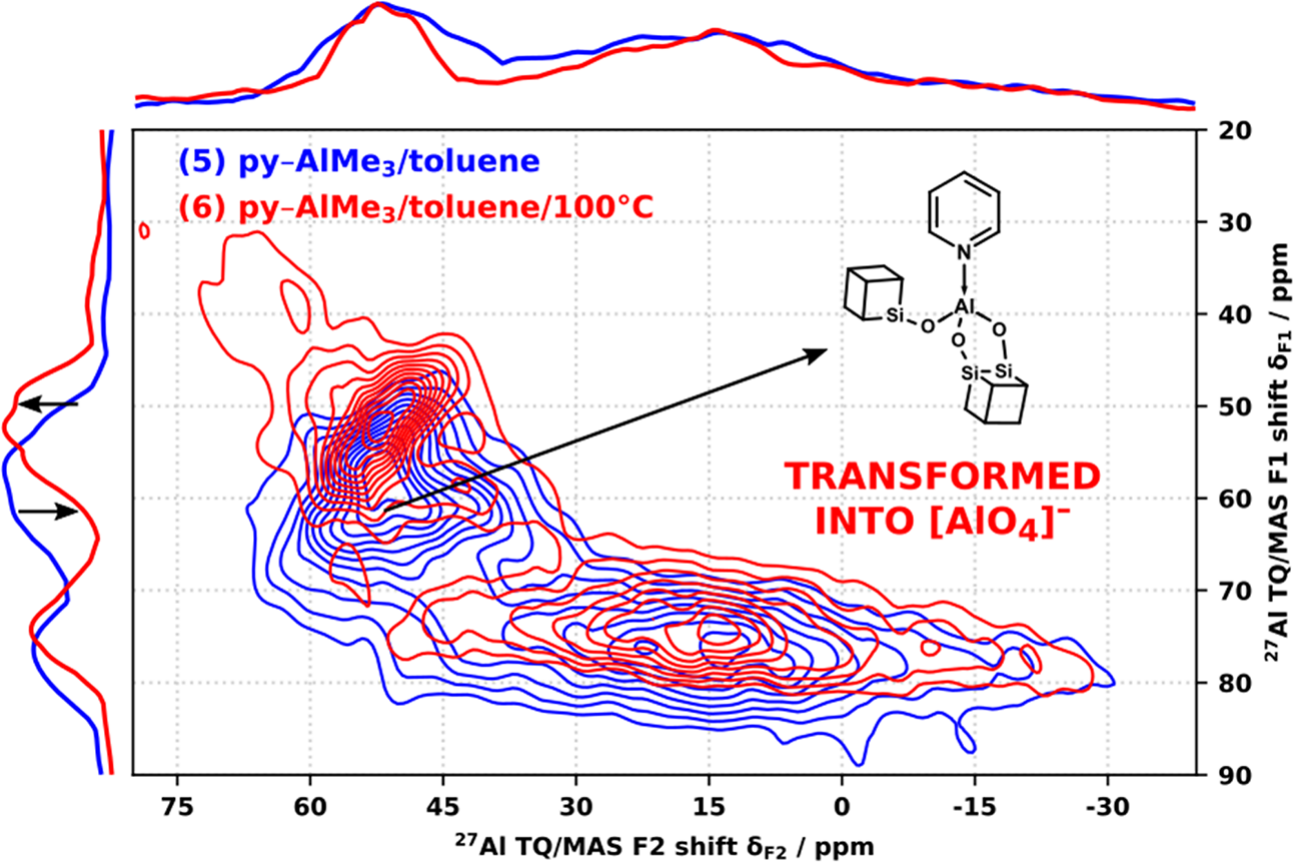
^27^Al TQ/MAS NMR spectra of products **5** (blue) and **6** (red) (20 kHz MAS).

The exact nature of the observed [AlO_4_]^−^ resonances is somewhat uncertain. Both the δ_F1_ and δ_iso_ NMR shifts of all the [AlO_4_]^−^ resonances in products **1**–**15** fall within the range defined by the resonances **16/A–C** (Figure 8), with the fitted *C*_Q_ values comparable or slightly larger to **16/A–C**. In many cases, a direct correspondence between the resonances in **1**–**15** and those in **16** could be established based on δ_F1_ but the fitted δ_iso_ values failed to match the correspondences in δ_F1_, with most [AlO_4_]^−^ resonances in **1**–**15** more closely matching to the resonance **16/C**. The comparison of products **5** and **6** (Figure 9) provides further insights. The higher reaction temperature in **6** resulted in the reduction of the py–Al(O_2_CUBE)(OCUBE) resonance and an increase in the resonance assigned to [AlO_4_]^−^, indicating that the former is transformed into the latter, preferentially over the more sterically crowded py–Al(OCUBE)_3_. Moreover, the [AlO_4_]^−^ resonances in **6** show signs of thermal relaxation into narrower (slightly lower *C*_Q_) features with three distinct maxima, establishing a direct match to resonances **16/A–C** in δ_F1_. This implies that the formed [AlO_4_]^−^ sites must possess at least one bidentate CUBE and that both the transformation of [L–AlO_3_] and the condensation of [AlCl_4_]^−^ converge to the same types of pseudotetrahedral sites. A comparison to the DFT predictions affirms the presence of bidentate CUBEs as the best match is found with [Al(O_2_CUBE)(OCUBE)_2_]^−^ and less so with [Al(O_2_CUBE)_2_]^−^ (Table 2), both being indistinguishable based on δ_iso_/*C*_Q_ and most closely matching to the resonance **16/A**. The better agreement of η in the case of the former (0.394) over the latter (0.018) is not insignificant as the experimental fits of all the resonances assigned as [AlO_4_]^−^ resulted in η ≈ 0.5 quite consistently. On the other hand, the assignment of the observed resonances as the originally sought [Al(OCUBE)_4_]^−^ is disfavored as the predicted |*C*_Q_| is smaller by at least 50%. While the inclusion of the weakly interacting countercation [Me_4_N]^+^ does increase η, the larger observed *C*_Q_ could not be explained this way. Additionally, the assignment of any of the observed sites as [AlCl(OCUBE)_3_]^−^ or [AlCl(O_2_CUBE)(OCUBE)]^−^ (incomplete condensation) is strongly disfavored based on the much larger predicted δ_iso_ and |*C*_Q_|. The correspondence between the two candidate types of sites and the three observed maxima **16/A–C** could not be further clarified; however, the thermal relaxation observed in **6** and its close match to **16/A–C** would suggest that **16/A–C** may simply represent a band of conformers of a single type of site, with three favored conformations of a decreasing symmetry (increasing *C*_Q_).

### Further Computational Predictions of ^27^Al MAS NMR Parameters

**Figure 10:**
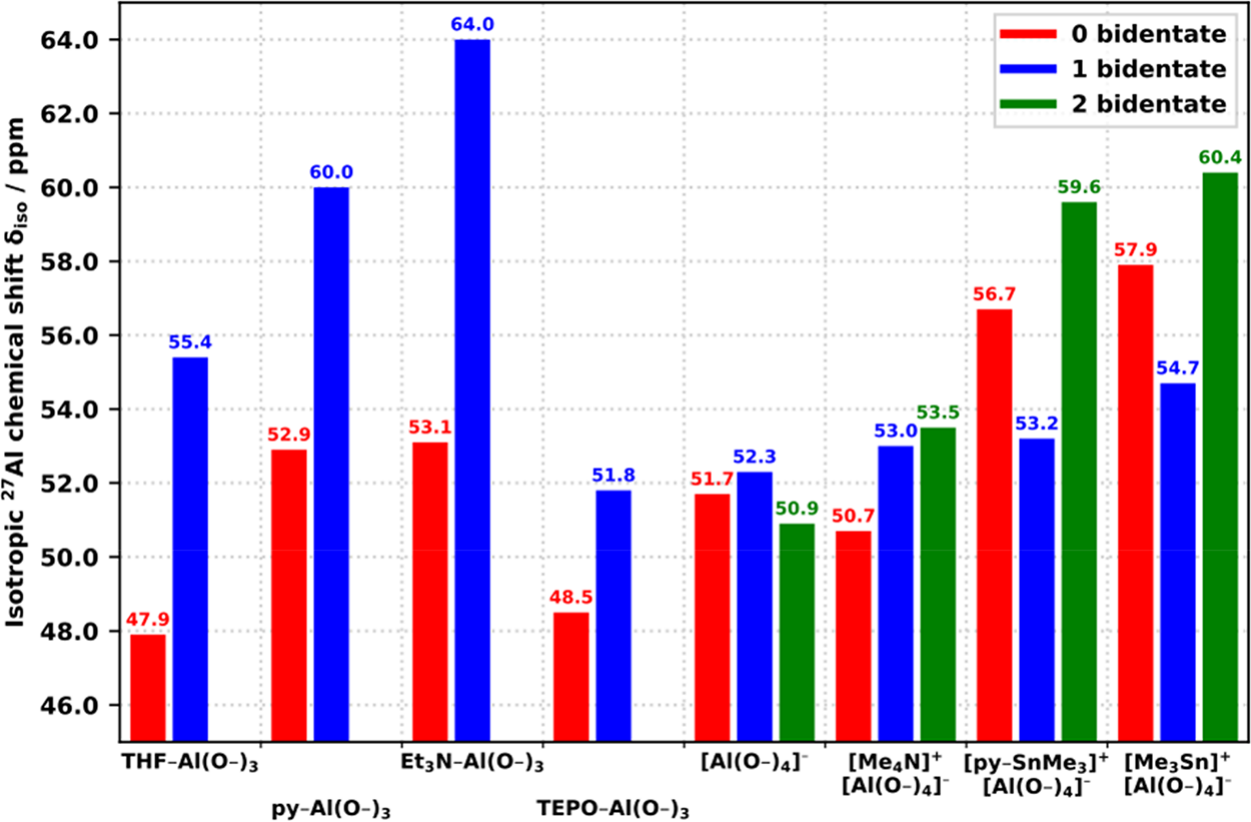
Comparison of predicted δ_iso_ for various sites based on the number of bidentate CUBEs (terminated by −OMe).

**Figure 11:**
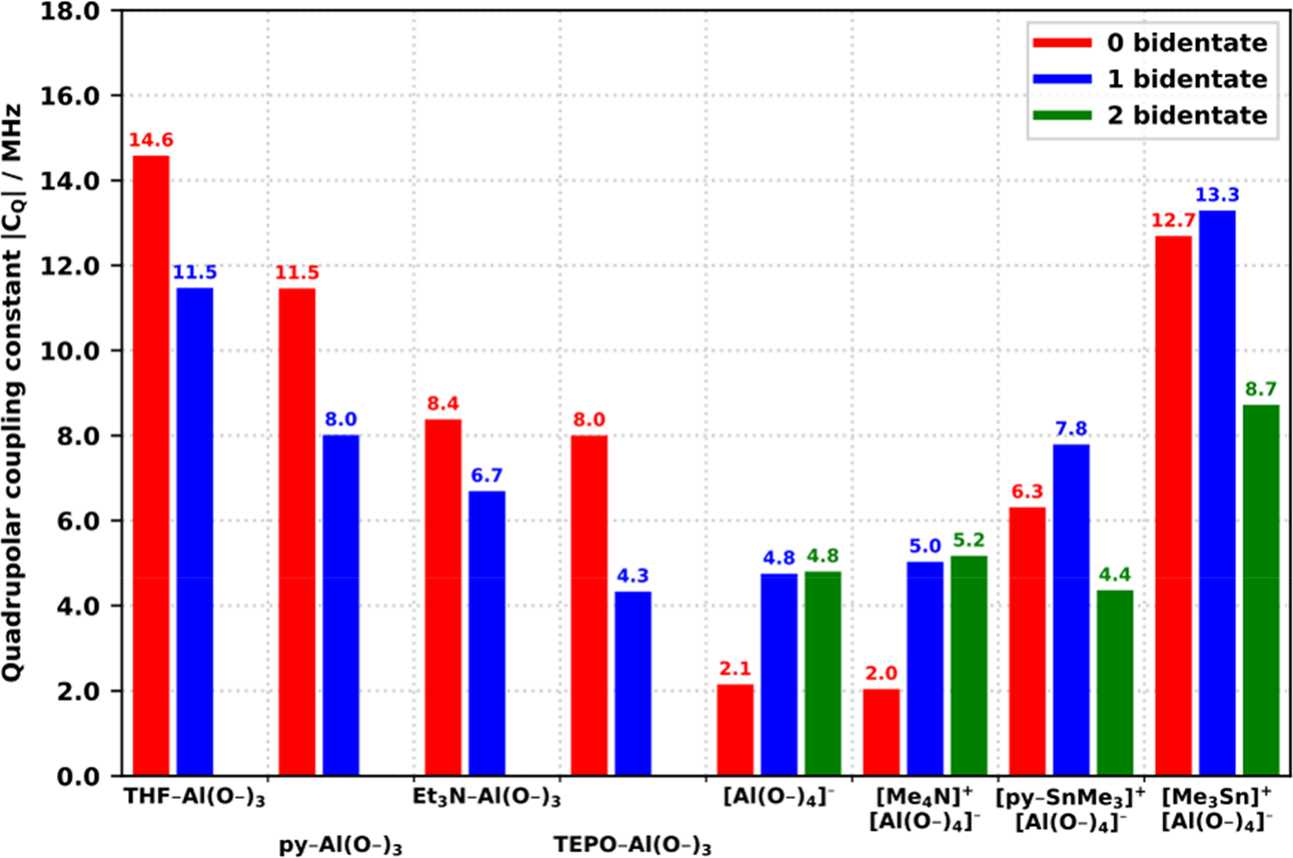
Comparison of predicted |*C*_Q_| for various sites based on the number of bidentate CUBEs (terminated by −OMe).

**Figure 12:**
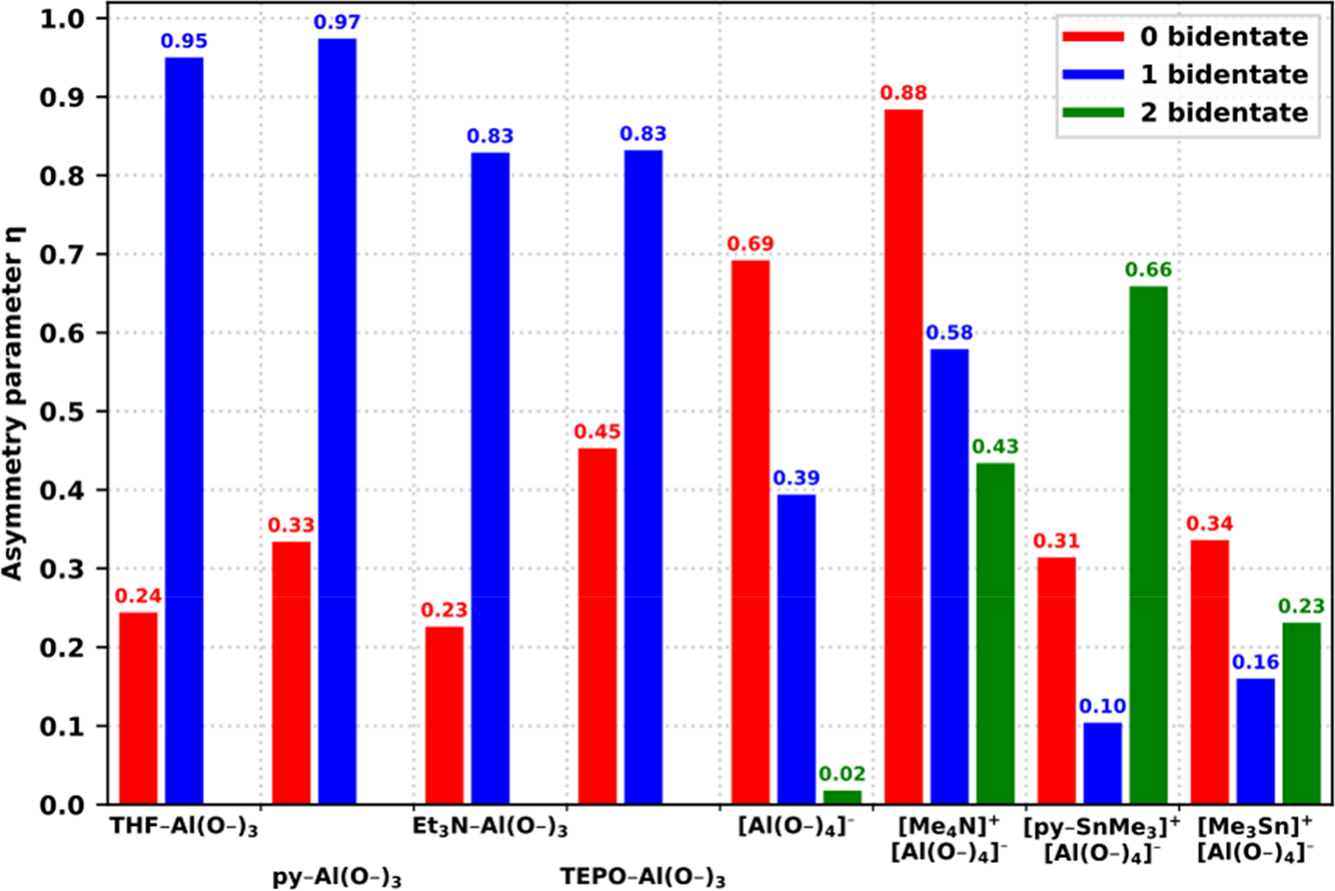
Comparison of predicted η for various sites based on the number of bidentate CUBEs (terminated by −OMe).

Since the scope of the NMR parameter calculations went beyond the portfolio of the experimentally observed sites, further supporting correlations can be established based on the computational results only. Figures 10, 11, and 12 illustrate several important trends.

First, the isotropic chemical shifts of structures with [AlO_3_N] coordination spheres are systematically higher than those of the structures with the [AlO_4_] environment (Figure 10). This trend has been well documented in the literature and it is generally attributed to the increased *p*-character of Al–N bonds over Al–O.^53^

Second, the introduction of a bidentate CUBE in the [L–AlO_3_] type of sites results in a significant increase of δ_iso_ and η, along with a decrease of |*C*_Q_|, indicating an overall symmetrization of the environment with a shift from a unidirectional (|*V*_33_| ≫ |*V*_22_| ≈ |*V*_11_|) to a bidirectional asymmetry (|*V*_33_| ≈ |*V*_22_| ≫ |*V*_11_|) of the EFG tensor. In the case of the symmetric [Al(OCUBE)_4_]^−^, the presence of bidentate CUBEs does not appreciably affect δ_iso_ while |*C*_Q_| is doubled by the introduction of the first bidentate CUBE and further unaffected by the introduction of the second one. This is consistent with the idea that the initial approximate *T*_d_ symmetry is first reduced to C_2v_ by the introduction of a dominant *C*_*2*_ symmetry axis through the first bidentate CUBE while the dominance of the axis is unchanged with the transition to D_2d_ by the introduction of the second bidentate CUBE. In contrast to |*C*_Q_|, η decreases monotonically to ∼0, pointing to the increasing dominance of a single direction in the EFG tensor and the significant off-main-axis symmetrization due to the appearance of a S_4_ axis in the transition from C_2v_ to D_2d_.

Finally, the effect of the presence of a countercation is illustrated. In the geometry optimizations, the quite inert [Me_4_N]^+^ ion was moved away from the Al center to an equilibrium N–Al distance d(N–Al) = 5.025, 4.986, and 4.920 Å for Al in [Al(OCUBE)_4_]^−^, [Al(O_2_CUBE)(OCUBE)_2_]^−^, and [Al(O_2_CUBE)_2_]^−^, respectively. Consequently, it bears little effect on δ_iso_ and |*C*_Q_| while η is systematically increased compared to the naked anions, reflecting the additional symmetry breaking due to the proximity of a point charge. The relevant cations [py–SnMe_3_]^+^ and [Me_3_Sn]^+^ (*vide infra*) exhibit progressively stronger interactions with the Al–O–Si bridges at d(Sn–O) = 2.496–2.551 and 2.171–2.201 Å, resulting in d(Sn–Al) = 3.579–3.707 and 3.352–3.443 Å for the two cations, respectively. In the case of [Me_3_Sn]^+^, the Sn–O distance approaches the average value (1.987 Å) reported for anhydrous CUBE from the single-crystal X-ray diffraction measurements.^13^ As a result, the NMR spectroscopic parameters are progressively more influenced in the general direction of increasing δ_iso_ and |*C*_Q_|, consistent with the increasing dominance of the cation as a symmetry-breaking element and its deshielding effect.

Additionally, Figures S42–S44 map the expected effect of the presence of residual reactive groups Al–Cl and Al–Me due to incomplete condensation. In general, δ_iso_ is increased slightly from 47.9–53.1 to 62.0–73.2 ppm by the presence of Al–Cl but more dramatically to 84.5–99.5 ppm by Al–Me (Figure S42) in consistence with the literature.^7,53^ For the [L–AlXO_2_] sites, |*C*_Q_| is slightly decreased (L = THF, py) or unaffected (L = Et_3_N, TEPO) by Al–Cl while it is increased dramatically by the presence of Al–Me (Figure S43).^7^ In both cases, η is increased comparably (Figure S44). Conversely, for the [AlClO_3_]^−^ sites, |*C*_Q_| is increased compared to the corresponding [AlO_4_]^−^, reflecting the lowered symmetry.

Finally, Figures S45–S47 benchmark the effect of the functional groups used to terminate the corners of the CUBEs on three selected structures: py–Al(OCUBE)_3_, Et_3_N–Al(OCUBE)_3_, and [Al(OCUBE)_4_]^−^. The termination by −OMe used in this work was chosen as a compromise between the computationally cheap but problematic −OH (strong electric dipoles, undesirable hydrogen bonding) and the authentic but computationally expensive −OSnMe_3_. The benchmark data indicate that δ_iso_ (Figure S45) and η (Figure S47) are reproduced well with −OMe termination compared to −OSnMe_3_, with the exception of the δ_iso_ of [Al(OCUBE)_4_]^−^, which is significantly overestimated with −OMe compared to −OSnMe_3_ as well as −OH and −OSiMe_3_. Most importantly, the |*C*_Q_| values predicted with −OMe termination are systematically underestimated by an average of 23% compared to −OSnMe_3_, providing yet another explanation for the generally observed positive deviation of the experimental *C*_Q_ from the DFT predictions (Figure S46). The significantly higher Sn^4+^ cation polarizability in comparison to Si^4+^ cation^56^ strongly affects the EFG tensor and the local geometry of aluminum atoms, which results in the increase of experimental *C*_Q_ values.

### Proposed Mechanism of the Formation of [AlO_4_]^−^

The experimental results established the presence of unexpected [AlO_4_]^−^ centers in the aluminosilicate products from the reactions of the L–AlX_3_ precursors with the CUBE building block. Furthermore, there appears to be a correlation between their formation and the observation of excess SnMe_4_. The process by which the excess SnMe_4_ is formed is certainly less favored than the primary condensation reactions of Al–Cl, Al–Me, and Al–Et since full condensation was reached for nearly all products (**1**–**11** and **13**–**15**), yet a full transformation of all the [L–AlO_3_] moieties to [AlO_4_]^−^ was never observed. Also, the process was observed to be strongly suppressed with more extensive cross-linking at higher AlX/CUBE ratios while it showed no sensitivity to the type of the primary condensation reaction (and the type of the condensation byproduct). From this, it stands to reason that the formation of [AlO_4_]^−^ sites takes place in the late stages of the synthetic procedure—during the warm-up and heating—after the precursors have lost their initial identity to invariably become [L–AlO_3_] centers and after an extensive, hindering oligomer network may or may not have formed. Extensive cross-linking is expected only at the late stages of a step-growth condensation process. The strong sensitivity to the cross-linking extent, the observed evidence of additional connectivity (even to the point of sustaining microporosity in product of reaction **6**), and the lack of evidence for the participation of the byproducts from the primary condensation reaction (SnMe_3_Cl, SnMe_4_, or SnMe_3_Et), constitute facts that all point to the unreacted Me_3_Sn–O–CUBE groups as the sole source of the additional tin removed in the form of excess SnMe_4_. Since a new negatively charged species is formed, the charge must be compensated by the formation of a countercation, which would represent the natural site for the liberated primary ligand (L = THF, py, Et_3_N, TEPO) to coordinate, as no free ligand was observed to leave the product.

**Figure 13:**
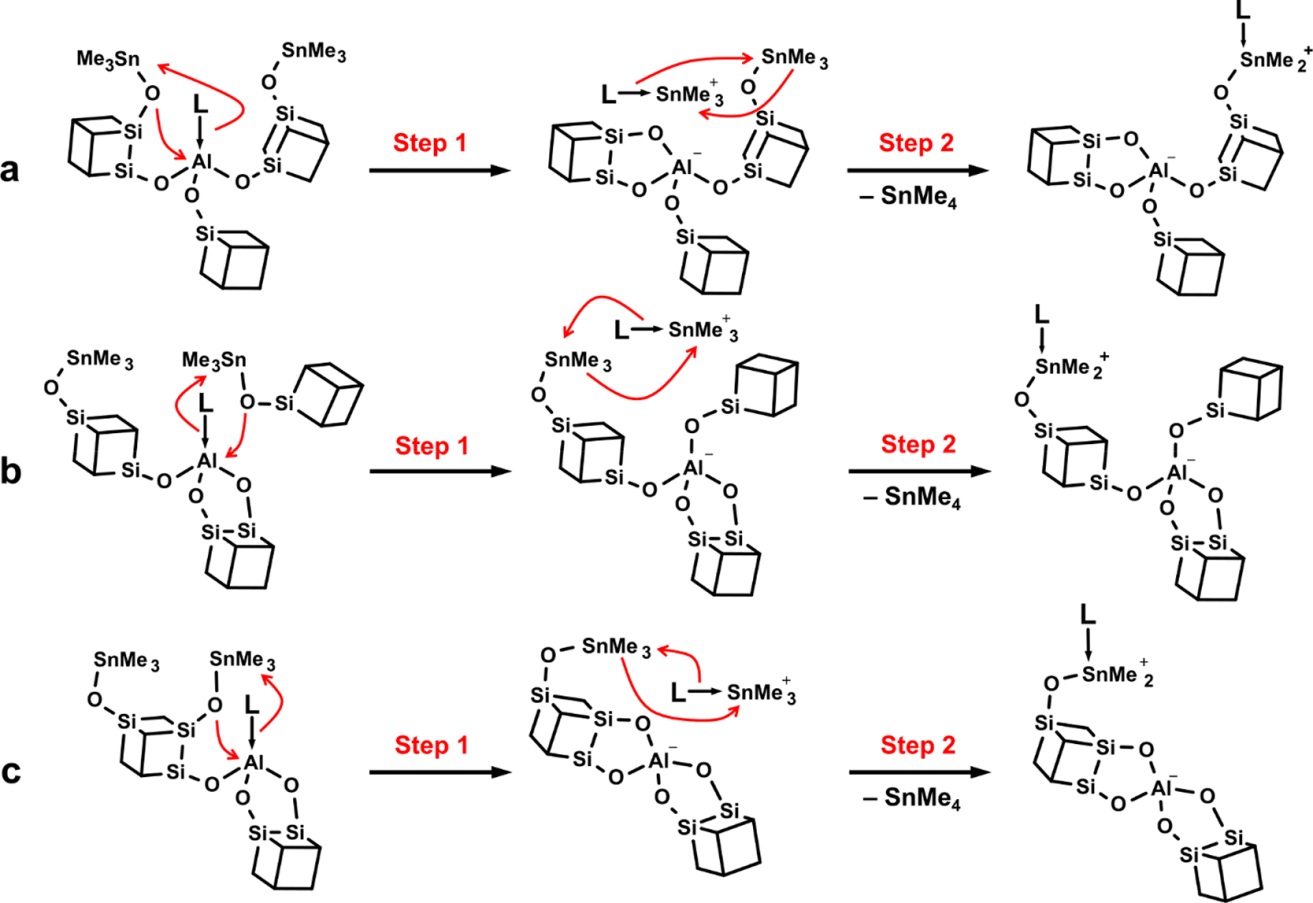
Proposed two-step pathways leading to the transformation of [L–AlO_3_] to [AlO_4_]^−^ with one bidentate CUBE.

Based on the aforementioned evidence, a two-step process is proposed (Figure 13). In the first step, a [L–AlO_3_] center is attacked by an oxygen atom of a proximal Me_3_Sn–O–CUBE group, acting as a nucleophile. A new Al–O–CUBE bond is established and the expelled primary ligand L is captured by the emerging cation [Me_3_Sn]^+^ in order to stabilize it.

In the second step, the relatively free-roaming [L–SnMe_3_]^+^ cation undergoes an exchange reaction with yet another Me_3_Sn–O–CUBE group in the vicinity, exchanging L for Me^–^ to become the observed additional SnMe_4_. As the Al centers do not participate, this step should only be affected by the strength of the ligand (and the solvent) and it should be independent of any structural properties of the oligomer network. The presence of the intermediate cation [L–SnMe_3_]^+^ and the final [L–SnMe_2_O–CUBE]^+^ group is inferred based on the formation of excess SnMe_4_ as their presence could not be confirmed spectroscopically. It is suspected that their ^1^H and ^13^C NMR resonances are too similar to those of the Al-bound ligands and the remaining Me_3_Sn–O–CUBE groups to allow for resolution in the MAS NMR spectra.

Since the ^27^Al TQ/MAS NMR analysis supports the presence of at least one bidentate CUBE at each [AlO_4_]^−^ center, only pathways leading to such arrangements are considered. In the simplest case, the initial L–Al(OCUBE)_3_ center is attacked by a neighboring Me_3_SnO– group of one of the directly connected CUBEs, making it bidentate (Figure 13a). Based on a series of structure optimizations using molecular mechanics, only a single mode of bidentate binding was found feasible—along the edge of the CUBE. While this pathway may be partially responsible for the transformation and it may somewhat increase the local rigidity, it cannot explain the observed dramatic increase in the long-range connectivity (even beyond the point of the sol–gel transition in reaction **6**) and the preferential disappearance of L–Al(O_2_CUBE)(OCUBE) over L–Al(OCUBE)_3_ as observed by ^27^Al TQ/MAS NMR (Figure 9). Therefore, a second, presumably more preferred pathway (Figure 13b) is proposed where the L–Al(O_2_CUBE)(OCUBE) center is attacked by a Me_3_SnO– group of a more distant CUBE, creating new long-range connectivity. Although the first pathway may benefit from the enforced proximity of the Me_3_SnO– group, the apparent preference for the second pathway can be supported by the more sterically favorable conditions at the aluminum center (only two CUBEs in the coordination sphere) and the fact that, due to its freedom, the incoming Me_3_SnO– group can approach from a more favorable direction. As the ^27^Al TQ/MAS NMR analysis could also support the presence of [Al(O_2_CUBE)_2_]^−^ sites, the participation of a third pathway starting from L–Al(O_2_CUBE)(OCUBE) cannot be ruled out (Figure 13c).

### The Proposed Source of SnMe_4_ in the Reactions of [AlCl_4_]^−^

**Figure 14:**
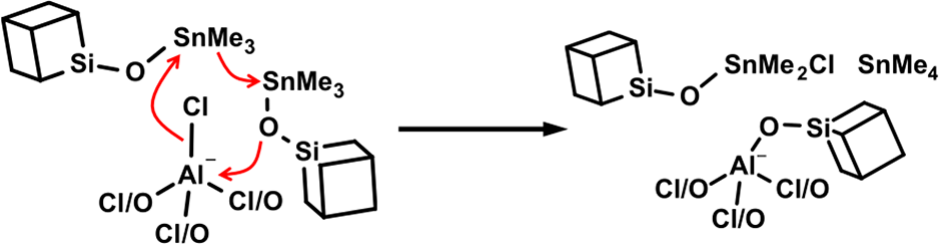
Proposed trimolecular mechanism explaining the evolution of SnMe_4_ in the condensation reaction of [AlCl_4_]^−^.

The previous part of the discussion provided an explanation for the combined appearance of the [AlO_4_]^−^ moiety together with the unexpected formation of SnMe_4_ in the reactions of L–AlX_3_ compounds. This, however, cannot explain the observation of significant amounts of SnMe_4_ in reactions with [Me_4_N] [AlCl_4_] (Table 1), where no such transformation is possible and no other types of Al sites were observed (Figure 8e). A closer analysis revealed a fundamental difference—the SnMe_4_ is generated instead of SnMe_3_Cl and not in addition to it, resulting in exactly 4 eq. of byproducts liberated per Al (average connectivity 3.98). Based on the experimental observation that the missing Cl does not leave the material in any form, a synchronous trimolecular mechanism is proposed as the simplest possible explanation (Figure 14). In an otherwise regular primary condensation reaction, the activated Cl^–^ nucleophile attacks a second Me_3_Sn–O–CUBE group, polarizing it and, in turn, causing it to transfer Me^–^ nucleophile to the first, activated Me_3_Sn–O–CUBE group. It is suspected that the generated Cl–Me_2_Sn–O–CUBE would be similarly difficult to distinguish from CUBE as the proposed cations [L–SnMe_3_]^+^ and [L–Me_2_Sn–O–CUBE]^+^.

## Conclusions

This work targeted the synthesis of four-coordinated aluminosilicate sites of general formulas [L–AlO_3_] and [AlO_4_]^−^, embedded in loose silicate matrices by utilizing the first step of a previously published two-step nonhydrolytic sol–gel procedure. The synthetic procedure proved to be flexible enough to accommodate a multitude of ligands, reactive groups, and solvents while it showed convergent behavior, leading only to several types of sites with well-defined ^27^Al NMR signatures. ^27^Al TQ/MAS NMR spectroscopy allowed for the observation and confident assignment of Lewis acid sites of general formulas L–Al(OCUBE)_3_ (L = THF, py, Et_3_N, TEPO) and L–Al(O_2_CUBE)(OCUBE) (L = THF, py). While the products always contained the targeted Lewis acid species, only TEPO–Al(OCUBE)_3_ could be obtained as a true single-site material. For weaker ligands, a considerable amount of unexpected [Al(O_2_CUBE)(OCUBE)_2_]^−^ and possibly [Al(O_2_CUBE)_2_]^−^ sites was also formed through a side reaction. This is contrary to the conclusions of Styskalik et al.^16^ who assigned the singular, observed symmetric ^27^Al NMR resonances at ∼49 ppm to the Lewis acid species. From experimental and computational results described here, it is now clear that these resonances correspond to the unexpected [AlO_4_]^−^ species while the species of interest remained invisible in the 1D ^27^Al NMR spectra due to insufficient excitation. Our investigations have successfully characterized these sites and established a good correlation between the Lewis acid site symmetry and its observed *C*_Q_ (4.4–15.1 MHz). Moreover, the experimentally obtained values of δ_iso_ and *C*_Q_ compare well to the predictions calculated by DFT methods. The experimental *C*_Q_ values are systematically increased with respect to the predictions, reflecting the additional disorder of the real-world amorphous systems compared to the fully relaxed model structures. The emergence of the synthetically undesirable [AlO_4_]^−^ species is attributed to the nucleophilic attack of the Me_3_SnO– groups of the spherosilicate building block at the aluminum site due to its Lewis acid properties.

## Supporting Information Available

(S1) Synthesis and characterization data of precursors, (S2) auxiliary methods and composition calculation procedures, (S3) product synthesis and characterization data, and (S4) ^27^Al NMR parameter calculation data (PDF)
