# Supplementary material for: Lewis Acidic Aluminosilicates: Synthesis, 27Al MQ/MAS NMR, and DFT-Calculated 27Al NMR Parameters
Source: Inorg Chem. 2024 Jan 25;63(5):2679–94. doi: 10.1021/acs.inorgchem.3c04035 (PMC10848260; doi:10.1021/acs.inorgchem.3c04035)
Supplement: Supplementary file 1 — ic3c04035_si_001.pdf [file ic3c04035_si_001.pdf]

Supporting information to the manuscript:

Lewis Acidic Aluminosilicates: Synthesis, ^{27}Al MQ/MAS NMR, and DFT-Calculated ^{27}Al NMR Parameters

Martin Kejik,[a] Jiri Brus,[b] Lukas Jeremias,[c] Lucie Simonikova,[a] Zdenek Moravec,[a] Libor Kobera,[b] Ales Styskalik,[a] Craig E. Barnes,[d] Jiri Pinkas.[a]*

[a] Department of Chemistry, Faculty of Science, Masaryk University, Kotlarka 2, CZ-61137 Brno, Czech Republic.

[b] Institute of Macromolecular Chemistry, Czech Academy of Sciences, Department of NMR Spectroscopy, Heyrovskeho nam. 2, CZ-16206 Prague, Czech Republic.

[c] Mendel University in Brno, Department of Chemistry and Biochemistry, CZ-61300 Brno, Czech Republic.

[d] University of Tennessee, Department of Chemistry, Knoxville, TN 37996-1600, United States.

* Corresponding author. E-mail address: jpinkas@chemi.muni.cz

Table of Contents

S1. Synthesis and characterization data of precursors 6

Py–$AlMe_3$ 6

Et_3N–$AlMe_3$ 6

TEPO–$AlMe_3$ 7

[Me_4N] [$AlCl_4$] 7

S2. Auxiliary methods and composition calculation procedures 8

Solution NMR 8

FTIR spectroscopy 8

ICP-OES 8

N_2 adsorption porosimetry 9

Table S1. The DC and composition calculation methodology. 10

S3. Product synthesis and characterization data 13

Product 1 (THF–$AlCl_3$/THF) 13

Figure S1. ^{27}Al{^{1}H} MAS NMR spectrum of product **1** 13

Figure S2. ^{27}Al TQ/MAS NMR spectrum of product **1** 14

Table S2. Slices through the ^{27}Al TQ/MAS NMR spectrum of product **1** and the corresponding line shape simulation fits. 14

Product 2 (0.5 py–$AlCl_3$/THF) 16

Figure S3. ^{27}Al{^{1}H} MAS NMR spectrum of product **2** 16

Figure S4. ^{27}Al TQ/MAS NMR spectrum of product **2** 17

Table S3. Slices through the ^{27}Al TQ/MAS NMR spectrum of product **2** and the corresponding line shape simulation fits 17

Product 3 (py–$AlCl_3$/THF) 19

Figure S5. ^{27}Al{^{1}H} MAS NMR spectrum of product **3** 19

Figure S6. ^{27}Al TQ/MAS NMR spectrum of product **3** 20

Table S4. Slices through the ^{27}Al TQ/MAS NMR spectrum of product **3** and the corresponding line shape simulation fits 20

Product 4 (0.5 py–$AlMe_3$/toluene) 22

Figure S7. ^{27}Al{^{1}H} MAS NMR spectrum of product **4** 22

Figure S8. ^{27}Al TQ/MAS NMR spectrum of product **4** 23

Table S5. Slices through the ^{27}Al TQ/MAS NMR spectrum of product **4** and the corresponding line shape simulation fits 23

Product 5 (py–$AlMe_3$/toluene) 25

Figure S9. ^{27}Al{^{1}H} MAS NMR spectrum of product **5** 25

Figure S10. ^{27}Al TQ/MAS NMR spectrum of product **5** ... 26

Table S6. Slices through the ^{27}Al TQ/MAS NMR spectrum of product **5** and the corresponding line shape simulation fits ... 26

Product 6 (py–AlMe$_3$/toluene/100 °C) ... 28

Figure S11. ^{27}Al{^{1}H} MAS NMR spectrum of product **6** ... 28

Figure S12. ^{27}Al TQ/MAS NMR spectrum of product **6** ... 29

Table S7. Slices through the ^{27}Al TQ/MAS NMR spectrum of product **6** and the corresponding line shape simulation fits ... 29

Product 7 (0.5 py–AlMe$_3$/THF) ... 31

Figure S13. ^{27}Al{^{1}H} MAS NMR spectrum of product **7** ... 31

Figure S14. ^{27}Al TQ/MAS NMR spectrum of product **7** ... 32

Table S8. Slices through the ^{27}Al TQ/MAS NMR spectrum of product **7** and the corresponding line shape simulation fits ... 32

Product 8 (py–AlMe$_3$/THF) ... 34

Figure S15. ^{27}Al{^{1}H} MAS NMR spectrum of product **8** ... 34

Figure S16. ^{27}Al TQ/MAS NMR spectrum of product **8** ... 35

Table S9. Slices through the ^{27}Al TQ/MAS NMR spectrum of product **8** and the corresponding line shape simulation fits ... 35

Product 9 (py–AlEt$_3$/toluene) ... 37

Figure S17. ^{27}Al{^{1}H} MAS NMR spectrum of product **9** ... 37

Figure S18. ^{27}Al TQ/MAS NMR spectrum of product **9** ... 38

Table S10. Slices through the ^{27}Al TQ/MAS NMR spectrum of product **9** and the corresponding line shape simulation fits ... 38

Product 10 (0.5 Et$_3$N–AlMe$_3$/toluene) ... 40

Figure S19. ^{27}Al{^{1}H} MAS NMR spectrum of product **10** ... 40

Figure S20. ^{27}Al TQ/MAS NMR spectrum of product **10** ... 41

Table S11. Slices through the ^{27}Al TQ/MAS NMR spectrum of product **10** and the corresponding line shape simulation fits ... 41

Product 11 (Et$_3$N–AlMe$_3$/toluene) ... 43

Figure S21. ^{27}Al{^{1}H} MAS NMR spectrum of product **11** ... 43

Figure S22. ^{27}Al TQ/MAS NMR spectrum of product **11** ... 44

Table S12. Slices through the ^{27}Al TQ/MAS NMR spectrum of product **11** and the corresponding line shape simulation fits ... 44

Product 12 (0.5 TEPO–AlCl$_3$/THF) ... 46

Figure S23. ^{27}Al{^{1}H} MAS NMR spectrum of product **12** ... 46

Figure S24. ^{27}Al TQ/MAS NMR spectrum of product **12** ... 47

Table S13. Slices through the ^{27}Al TQ/MAS NMR spectrum of product **12** and the corresponding line shape simulation fits 47

Product 13 (TEPO–AlCl$_3$/THF) 48

Figure S25. ^{27}Al{^{1}H} MAS NMR spectrum of product **13** 48

Figure S26. ^{27}Al TQ/MAS NMR spectrum of product **13** 49

Table S14. Slices through the ^{27}Al TQ/MAS NMR spectrum of product **13** and the corresponding line shape simulation fits 49

Product 14 (0.5 TEPO–AlMe$_3$/toluene) 50

Figure S27. ^{27}Al{^{1}H} MAS NMR spectrum of product **14** 50

Figure S28. ^{27}Al TQ/MAS NMR spectrum of product **14** 51

Table S15. Slices through the ^{27}Al TQ/MAS NMR spectrum of product **14** and the corresponding line shape simulation fits 51

Product 15 (TEPO–AlMe$_3$/toluene) 52

Figure S29. ^{27}Al{^{1}H} MAS NMR spectrum of product **15** 52

Figure S30. ^{27}Al TQ/MAS NMR spectrum of product **15** 53

Table S16. Slices through the ^{27}Al TQ/MAS NMR spectrum of product **15** and the corresponding line shape simulation fits 53

Product 16 (0.5 x 0.75 [Me$_4$N] [AlCl$_4$]/THF) 55

Figure S31. ^{27}Al{^{1}H} MAS NMR spectrum of product **16** 55

Figure S32. ^{27}Al TQ/MAS NMR spectrum of product **16** 56

Table S17. Slices through the ^{27}Al TQ/MAS NMR spectrum of product **16** and the corresponding line shape simulation fits 56

Table S18. Peak data for the deconvolution of ^{29}Si MAS NMR spectra of prepared products 58

Table S19. Degrees of condensation of –SnMe$_3$ groups calculated from different sources according to procedures described in Section S2 59

Figure S33. Correlation plot relating the DC of –SnMe$_3$ groups determined by gravimetry/NMR to the values obtained independently from ICP-OES and ^{29}Si MAS NMR deconvolution 60

Figure S34. FTIR spectra (KBr pellet) of products prepared from L –AlX$_3$ (X = Me, Et, Cl) 60

Figure S35. ^{13}C CP/TOSS MAS NMR spectra of products prepared from L –AlX$_3$ (X = Me, Et, Cl) 61

Figure S36. ^{13}C CP/TOSS MAS NMR spectra of products **12–15** 61

Figure S37. ^{1}H MAS NMR spectra of products **1**, **11**, and **16** 62

Figure S38. ^{1}H MAS NMR spectra of products **6**, **8**, and **9** 62

Figure S39. ^{1}H MAS NMR spectra of products **12**, **14**, and **15** 63

Table S20. Fitted ^{27}Al NMR parameters and assignments of all observed resonances in products **1–16** 64

Figure S40. The plot of all observed ^{27}Al TQ/MAS NMR resonances and relevant DFT-calculated models in the η/C_Q parameter space 65

Figure S41. ^{27}Al TQ/MAS NMR spectrum of product **15** 66

S4. ^{27}Al NMR parameter calculation data 67

Table S21. DFT-calculated ^{27}Al ssNMR parameters of model structures with CUBEs terminated by –OMe 67

Table S22. DFT-calculated ^{27}Al ssNMR parameters of selected structures with CUBEs terminated by –OH, –$OSiMe_3$, and –$OSnMe_3$ 68

Figure S42. Comparison of predicted δ_{iso} for various sites containing residual Al–Cl and Al–Me groups 69

Figure S43. Comparison of predicted $|C_Q|$ for various sites containing residual Al–Cl and Al–Me groups 69

Figure S44. Comparison of predicted η for various sites containing residual Al–Cl and Al–Me groups 70

Figure S45. Comparison of predicted δ_{iso} for selected sites with CUBEs terminated by –OH, –OMe, –$OSiMe_3$, and –$OSnMe_3$ groups 70

Figure S46. Comparison of predicted $|C_Q|$ for selected sites with CUBEs terminated by –OH, –OMe, –$OSiMe_3$, and –$OSnMe_3$ groups 71

Figure S47. Comparison of predicted η for selected sites with CUBEs terminated by –OH, –OMe, –$OSiMe_3$, and –$OSnMe_3$ groups 71

S6. References 72

S1. Synthesis and characterization data of precursors

Py–AlMe$_3$

AlMe$_3$ (2.0 M in toluene, 15.00 cm^3, 30.00 mmol) was slowly added by syringe to a stirred solution of pyridine (3.00 cm^3, 37.2 mmol) in toluene (20 cm^3) at room temperature. Subsequently, all volatiles were thoroughly removed under dynamic vacuum at room temperature to afford colorless crystals of the product (4.429 g, 97.7 %). The drying time had to be carefully controlled to avoid excessive product loss. The compound must be stored in gas-tight containers as it continuously resublimes to form large crystals on the container walls.

^{1}H NMR (C_6D_6) δ: 8.12 (m, 2H, *o*-Ar*H*), 6.66 (m, 1H, *p*-Ar*H*), 6.30 (m, 2H, *m*-Ar*H*), –0.21 (s, 9H, Al(C*H*$_3$)$_3$) ppm.

^{13}C{^{1}H} NMR (C_6D_6) δ: 147.24 (s, *o*-Ar), 140.05 (s, *p*-Ar), 125.43 (s, *m*-Ar), –7.27 (s, Al(*C*H$_3$)$_3$) ppm.

^{27}Al NMR (C_6D_6) δ: 176.95 ppm.

ICP-OES: found 16.7 wt% Al; calc. 17.9 wt% Al.

Et$_3$N–AlMe$_3$

AlMe$_3$ (2.0 M in toluene, 5.00 cm^3, 10.0 mmol) was slowly added by syringe to a stirred solution of Et$_3$N (1.50 cm^3, 10.8 mmol) in toluene (10 cm^3) at room temperature. All volatiles were then removed under dynamic vacuum at room temperature to afford colorless crystals of the product (1.690 g, 97.5 %). The same volatility considerations as with py–AlMe$_3$ apply.

^{1}H NMR (C_6D_6) δ: 2.23 (q, $^3J_{HH}$ = 7.30 Hz, 6H, CH$_3$C*H*$_2$N), 0.71 (t, $^3J_{HH}$ = 7.30 Hz, 9H, C*H*$_3$CH$_2$N), –0.41 (s, 9H, Al(C*H*$_3$)$_3$) ppm.

^{13}C{^{1}H} NMR (C_6D_6) δ: 48.01 (s, CH$_3$*C*H$_2$N), 9.33 (s, *C*H$_3$CH$_2$N), –6.00 (s, Al(*C*H$_3$)$_3$) ppm.

^{27}Al NMR (C_6D_6) δ: 177.60 ppm.

ICP-OES: found 16.4 wt% Al; calc. 15.6 wt% Al.

TEPO–$AlMe_3$

$AlMe_3$ (2.0 M in toluene, 3.82 cm^3, 7.64 mmol) was slowly added by syringe to a stirred solution of TEPO (1.0233 g; 7.6275 mmol) in toluene (10 cm^3) at room temperature. All volatiles were then removed under dynamic vacuum at room temperature to obtain the pure product as a colorless viscous liquid with a negligible vapor pressure (1.5813 g; 100.5 %).

^{1}H NMR (C_6D_6) δ: 1.03 (dq, $^2J_{PH}$ = 11.76 Hz, $^3J_{HH}$ = 7.68 Hz, 6H, $CH_3C\mathit{H}_2P$), 0.63 (dt, $^3J_{PH}$ = 17.59 Hz, $^3J_{HH}$ = 7.68 Hz, 9H, $C\mathit{H}_3CH_2P$), –0.36 (s, 9H, $Al(C\mathit{H}_3)_3$) ppm.

^{13}C{^{1}H} NMR (C_6D_6) δ: 18.31 (d, $^1J_{PC}$ = 67.51 Hz, $CH_3\mathit{C}H_2P$), 5.36 (d, $^2J_{PC}$ = 4.97 Hz, $\mathit{C}H_3CH_2P$), –5.55 (s, $Al(\mathit{C}H_3)_3$) ppm.

^{27}Al NMR (C_6D_6) δ: 166.03 ppm.

^{31}P{^{1}H} NMR (C_6D_6) δ: 66.73 ppm.

ICP-OES: found 14.0 wt% Al / 15.7 wt% P; calc. 13.1 wt% Al / 15.0 wt% P.

[Me_4N] [$AlCl_4$]

$AlCl_3$ (0.629 g, 4.72 mmol) and Me_4NCl (0.580 g, 5.29 mmol) powders were loaded together into a Schlenk vessel in drybox and sealed. The vessel was evacuated, cooled by liquid nitrogen and THF (~10 cm^3) was vapor transferred onto the solids. The vessel was then allowed to warm up to room temperature to facilitate controlled reaction. The resultant hazy suspension was filtered through a Schlenk frit and all volatiles were removed under dynamic vacuum at 60 °C to afford the product (1.125 g, 98.2 %) as a white powder.

^{1}H NMR ($CDCl_3$) δ: 3.44 (s, $C\mathit{H}_3N$) ppm.

^{13}C{^{1}H} NMR ($CDCl_3$) δ: 60.10 (s, $\mathit{C}H_3N$) ppm.

^{27}Al NMR ($CDCl_3$) δ: 104.08 ppm.

ICP-OES: found 11.5 wt% Al; calc. 11.1 wt% Al.

S2. Auxiliary methods and composition calculation procedures

Solution NMR

The ^{1}H, $^{13}C\{^{1}H\}$, ^{27}Al, and $^{31}P\{^{1}H\}$ NMR spectra of liquid or soluble samples were acquired on Bruker Avance III 300 MHz spectrometer with a BBFO probehead using standard 90°-pulse excitation at 298 K. The samples were dissolved in dry C_6D_6 or $CDCl_3$ (for $[Me_4N]$ $[AlCl_4]$ due to solubility issues) in the drybox and measured without delay in sealed 5 mm NMR tubes. ^{1}H (300.15 MHz) and ^{13}C (75.47 MHz) chemical shifts were referenced internally to the solvent residual peaks at ^{1}H: 7.15 / ^{13}C: 128.6 ppm for C_6D_6 and ^{1}H: 7.26 / ^{13}C: 77.4 ppm for $CDCl_3$.[1] ^{27}Al (78.21 MHz), and ^{31}P (121.50 MHz) chemical shifts were referenced externally to $[Al(H_2O)_6]^{3+}$, and 85% H_3PO_4, respectively (δ = 0 ppm). The collected samples of volatiles (400 μL) were mixed with C_6D_6 (200 μL) and quantitative ^{1}H spectra (50 scans/10 s delays) were used to determine the molar ratio of byproducts $n(SnMe_4)/n(SnMe_3X)$ (X = Cl, Et) through the integration of the central peak of the $–Sn(CH_3)_3$ moiety.

FTIR spectroscopy

Near-infrared spectra were acquired on a Bruker FTIR Tensor 27 spectrometer in the 4000–400 cm^{-1} range with a 2 cm^{-1} resolution. The sample (~1 mg) was mixed with FTIR-grade KBr (100–110 mg) in the drybox, homogenized, pressed in a threaded-hole press to form a semi-transparent window, and measured within 20 min. A short exposition to ambient air (<30 s) was inevitable and therefore, even though the samples do not contain any –OH groups, the spectra always contain the corresponding vibration bands (1636 cm^{-1}, 3435 cm^{-1}) with variable intensity.

ICP-OES

All samples were analyzed by ICP-OES for Al, Sn, and P (samples with TEPO) content in order to provide independent validation of the information obtained from gravimetric methods (degree of condensation, composition, initial stoichiometry). The precisely weighed sample (~60 mg) was digested by 2 cm^3 of a 1:1 mixture of 65% HNO_3 and 50% HF and diluted to 100.00 cm^3. The volumetric solutions were measured on the Thermo Scientific iCAP

6500 spectrometer in an axial observation mode at 1150 W plasma power with 0.65 and 12 dm^3 min^{-1} nebulizer and coolant gas flows respectively. Observed emissions: Al (396.152 and 394.401 nm), Sn (189.989 nm), P (177.495, 213.618, and 214.914 nm). The ICP-determined Sn content together with the mass of the product from gravimetry were used to calculate the degree of condensation of the tin groups $DC_E(SnMe_3)$ to provide further validation to the figures obtained from the combined gravimetry/^{1}H NMR approach. The calculation procedure is provided in Table S1 and the resulting DC values in Table S19.

N_2 adsorption porosimetry

The textural properties of prepared materials were characterized by nitrogen adsorption at 77.4 K on a Quantachrome Autosorb iQ gas adsorption instrument. The samples were outgassed at 60 °C (synthesis temperature) prior to measurement. The specific surface area SA_{BET} was calculated using the multipoint Brunauer-Emmett-Teller method (BET)[2,3] from at least 4 data points acquired between relative pressures p/p_0 = 0.02–0.30. The relevant points were determined as the non-decreasing part of the Rouquerol transform.[4] The total pore volume V_{tot} was calculated from the total gas uptake at p/p_0 = 0.97.

Table S1. The DC and composition calculation methodology.

1. Input reagents: CUBE

Mass of the CUBE: $m_{CUBE} = m_{flask+CUBE} - m_{flask}$

Molar amount of the CUBE: $n_{CUBE} = \frac{m_{CUBE}}{M_{CUBE}}$

where: $m_{flask+CUBE}$, m_{flask} … *weighed inputs,* $M_{CUBE} = 1855.18\ g\ mol^{-1}$

2A. Input reagents: L–AlMe$_3$, L–AlEt$_3$, and [Me$_4$N] [AlCl$_4$]

Mass of the Al complex: $m_{complex} = m_{vial+complex} - m_{vial}$

Molar amount of the Al complex: $n_{complex} = \frac{m_{complex}}{M_{complex}}$

where: $m_{vial+complex}$, m_{vial} … *weighed inputs,* $M_{complex}$ … *molar mass of the complex*

2B. Input reagents: L–AlCl$_3$, where L = THF, py, Et$_3$N (excess ligands are volatile)

Mass of AlCl$_3$: $m_{AlCl_3} = m_{Al-vial+AlCl_3} - m_{Al-vial}$

Molar amount of the Al complex: $n_{complex} = \frac{m_{AlCl_3}}{M_{AlCl_3}}$

Mass of the Al complex: $m_{complex} = n_{complex}\ M_{complex}$

where: $m_{Al-vial+AlCl_3}$, $m_{Al-vial}$ … *weighed inputs,* $M_{AlCl_3} = 133.34\ g\ mol^{-1}$, $M_{complex}$ … *molar mass of the complex (THF–AlCl$_3$: 205.45, py–AlCl$_3$: 212.44, py–AlMe$_3$: 151.18, py–AlEt$_3$: 193.26, Et$_3$N–AlMe$_3$: 173.27, TEPO–AlCl$_3$: 267.50, TEPO–AlMe$_3$: 206.24, [Me$_4$N][AlCl$_4$]: 242.94 g mol^{-1})*

2C. Input reagents: TEPO–AlCl$_3$ (excess TEPO is not volatile – stays in the product)

Mass of AlCl$_3$: $m_{AlCl_3} = m_{Al-vial+AlCl_3} - m_{Al-vial}$

Mass of TEPO: $m_{TEPO} = m_{L-vial+TEPO} - m_{L-vial}$

Molar amount of the Al complex: $n_{complex} = \frac{m_{AlCl_3}}{M_{AlCl_3}}$

Mass of the Al complex: $m_{complex} = m_{AlCl_3} + m_{TEPO}$

where: $m_{Al-vial+AlCl_3}$, $m_{Al-vial}$, $m_{L-vial+TEPO}$, m_{L-vial} … *weighed inputs,* $M_{AlCl_3} = 133.34\ g\ mol^{-1}$

3. Input reagents: common

Molar amount of the Al: $n_{Al} = n_{complex}$

Molar amount of the reactive groups: $n_{AlX} = N_{AlX}\ n_{complex}$

where: N_{AlX} … *the number of reactive groups (Al–Me, Al–Et or Al–Cl) per molecule of the complex*

4. The mass of the product (oligomers) and byproducts (from condensation + additional SnMe$_4$)

Mass of the product: $m_{product} = m_{flask+product} - m_{flask}$

Mass of the volatiles: $\Delta m = m_{flask+CUBE} + m_{complex} - m_{flask+product}$

where: $m_{flask+CUBE}$, $m_{flask+product}$, m_{flask} *… weighed inputs*

5A. Molar amounts of the byproducts: L–$AlEt_3$, L–$AlCl_3$, and [Me_4N] [$AlCl_4$] ($SnMe_3X$ from the condensation + additional $SnMe_4$)

Molar amount of the condensation byproduct: $n_{byproduct} = \frac{\Delta m}{M_{byproduct} + \left(\frac{n_{SnMe_4}}{n_{SnMe_3X}}\right)_{NMR} M_{SnMe_4}}$

Molar amount of the additional $SnMe_4$: $n_{SnMe_4} = \frac{\Delta m}{M_{SnMe_4} + \frac{1}{\left(\frac{n_{SnMe_4}}{n_{SnMe_3X}}\right)_{NMR}} M_{byproduct}}$

where: X = (Et, Cl), $\left(\frac{n_{SnMe_4}}{n_{SnMe_3X}}\right)_{NMR}$ *… determined by* ^1H *NMR integration,* $M_{SnMe_4} = 178.85\ g\ mol^{-1}$, $M_{byproduct}$ *… molar mass of the condensation byproduct ($SnMe_3Et$: 192.87, $SnMe_3Cl$: 199.27 g mol^{-1})*

5B. Molar amount of the byproduct: L–$AlMe_3$ ($SnMe_4$ only)

Molar amount of the condensation byproduct: $n_{byproduct} = min\left(\frac{\Delta m}{M_{SnMe_4}}, n_{AlX}\right)$

Molar amount of the additional $SnMe_4$: $n_{SnMe_4} = max\left(0, \frac{\Delta m}{M_{SnMe_4}} - n_{AlX}\right)$

where: $M_{SnMe_4} = 178.85\ g\ mol^{-1}$

6. Gravimetry output: stoichiometry, degree of condensation (DC), and composition

Reaction stoichiometry Al/CUBE and Al–X/CUBE: $\frac{n_{Al}}{n_{CUBE}}$ and $\frac{n_{AlX}}{n_{CUBE}}$

Degree of condensation of –$SnMe_3$ groups: $DC_G(SnMe_3) = \frac{n_{byproduct} + n_{SnMe_4}}{8\ n_{CUBE}}$

Degree of condensation of Al–X groups: $DC_G(AlX) = \frac{n_{byproduct}}{n_{AlX}}$

Additional $SnMe_4$ quantity: $DC_G(SnMe_4) = \frac{n_{SnMe_4}}{n_{Al}}$

Average connectivity at Al and CUBE: $\frac{n_{byproduct} + n_{SnMe_4}}{n_{Al}}$ and $8\ DC_G(SnMe_3)$

Predicted Al content in the product: $Al_G(wt\%) = \frac{n_{Al}\ M_{Al}}{m_{product}}$

Predicted Sn content in the product: $Sn_G(wt\%) = \frac{(8n_{CUBE} - n_{byproduct} - n_{SnMe_4})\ M_{Sn}}{m_{product}}$

where: $M_{Al} = 26.982\ g\ mol^{-1}$, $M_{Sn} = 118.71\ g\ mol^{-1}$

7. ICP-OES: Partially independent validation of *DC*$_G$($SnMe_3$)

Degree of condensation of –$SnMe_3$ groups: $DC_E(SnMe_3) = 1 - \frac{Sn_I(wt\%)\ m_{product}}{8\ n_{CUBE}\ M_{Sn}}$

where: $Sn_I(wt\%)$ *… Sn content determined by ICP-OES,* $M_{Sn} = 118.71\ g\ mol^{-1}$

8. ^{29}Si MAS NMR: Completely independent validation of *DC*$_G$($SnMe_3$)

Degree of condensation of –$SnMe_3$ groups: $DC_N(SnMe_3) = \frac{A(AlOSi)}{A(AlOSi)+A(SnOSi)}$

where: $A(AlOSi)$, $A(SnOSi)$ … *areas of deconvolution peaks corresponding to* ^{29}*Si resonances of the [AlOSi] and [SnOSi] moieties*

S3. Product synthesis and characterization data

Product 1 (THF–$AlCl_3$/THF)

Synthesis: $(Me_3Sn)_8Si_8O_{20}$ (2.1630 g, 1.1659 mmol), $AlCl_3$ (0.1537 g, 1.153 mmol), THF (solvent/ligand, 30 cm^3). **Byproducts:** 0.7812 g, *n(SnMe₄)/n(SnMe₃Cl)* = 0.087675.

IR (KBr, cm^{-1}) *ν*: 513 w, 542 m, 583 w, 623 m, 670 vw, 724 vw, 778 m (ν SiOSi), 875 vw, 918 vw, 1048 vs (ν_{as} SiOAl), 1127 vs (ν_{as} SiOSi), 1403 vw (δ_{as} CH_3), 2919 vw (ν_s CH_3), 2990 vw (ν_{as} CH_3), 3435 vw (ν O–H).

^{1}H MAS NMR *δ*: 3.6 (THF), 1.3 (THF), –0.4 (–OSn(CH_3)$_3$) ppm.

^{13}C TOSS NMR *δ*: 75.0 (THF), 27.8 (THF), –0.3 (–OSn(CH_3)$_3$) ppm.

^{29}Si MAS NMR *δ*: –101.9 ((CH_3)$_3$SnO*Si*(OSi)$_3$), –107.5 (AlO*Si*(OSi)$_3$) ppm.

Composition: ICP-OES: 2.07 wt% Al, 41.2 wt% Sn; **Gravimetry:** 1.92 wt% Al, 46.0 wt% Sn.

N_2 ads.: non-porous, V_{tot} 0.00713 cm^3 g^{-1}, Type III isotherm.

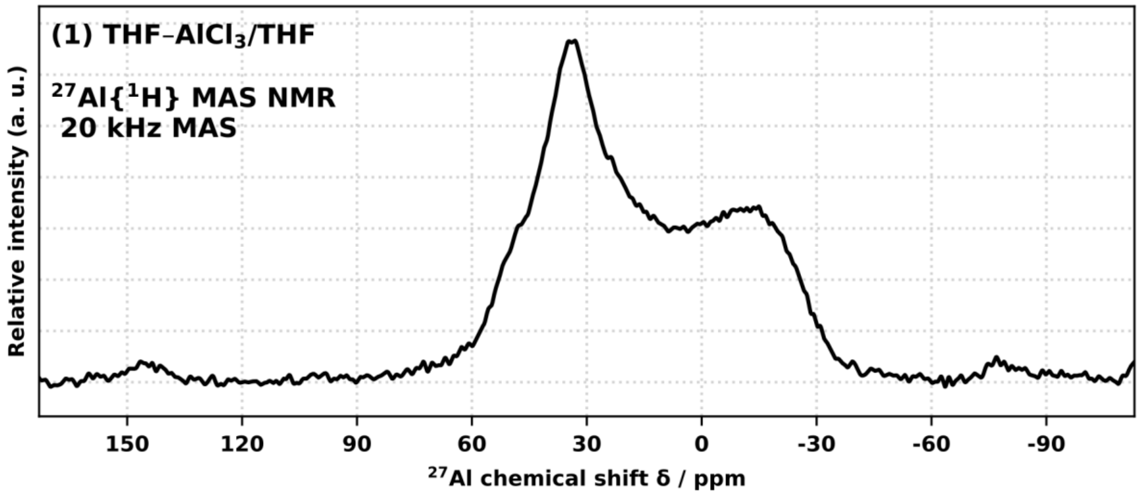

Figure S1. ^{27}Al{^{1}H} MAS NMR spectrum of product **1** (20 kHz MAS).

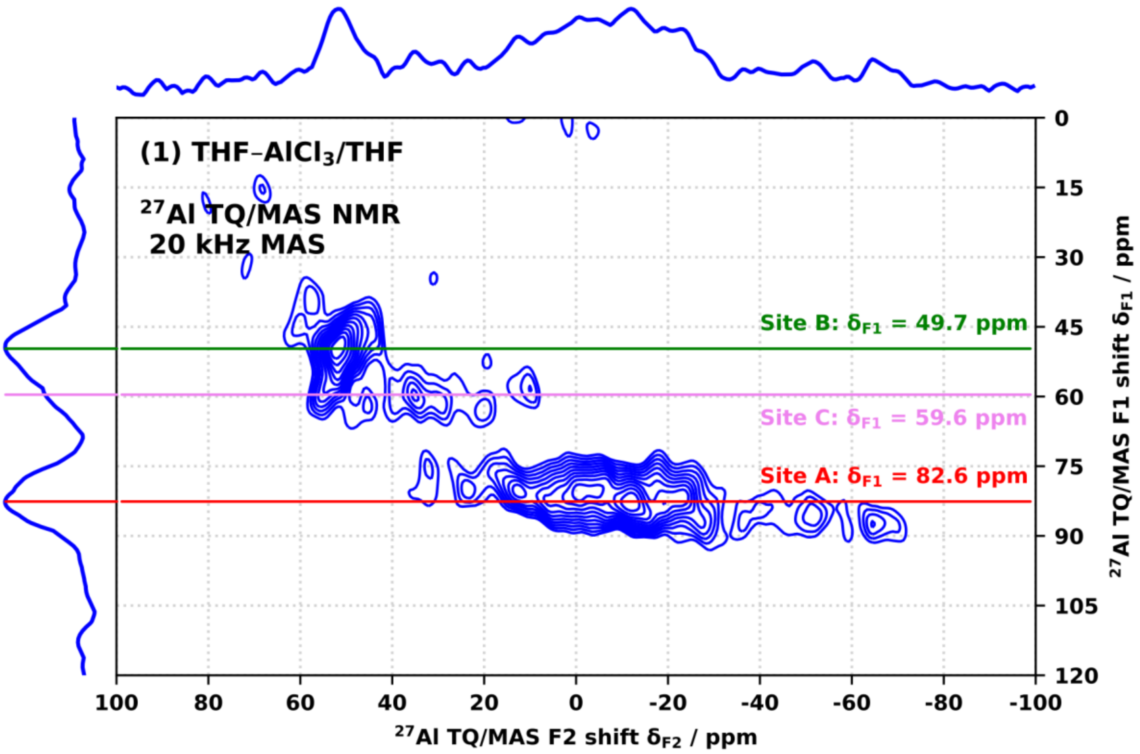

Figure S2. ^{27}Al TQ/MAS NMR spectrum of product **1** (20 kHz MAS).

Table S2. Slices through the ^{27}Al TQ/MAS NMR spectrum of product **1** and the corresponding line shape simulation fits.

Site	^{27}Al line shape simulation fit (quadrupole central transition)			
	δ_{iso} (ppm)	C_Q (MHz)	η	*LB* (fixed)
	38.102	15.144	0.683	1000
**A**	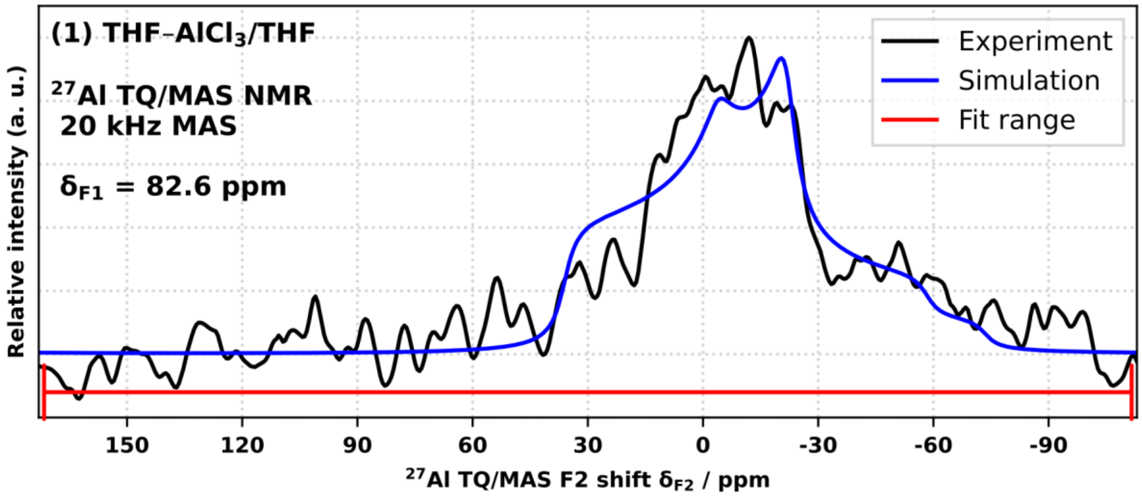			

	59.047	6.139	0.625	600

B

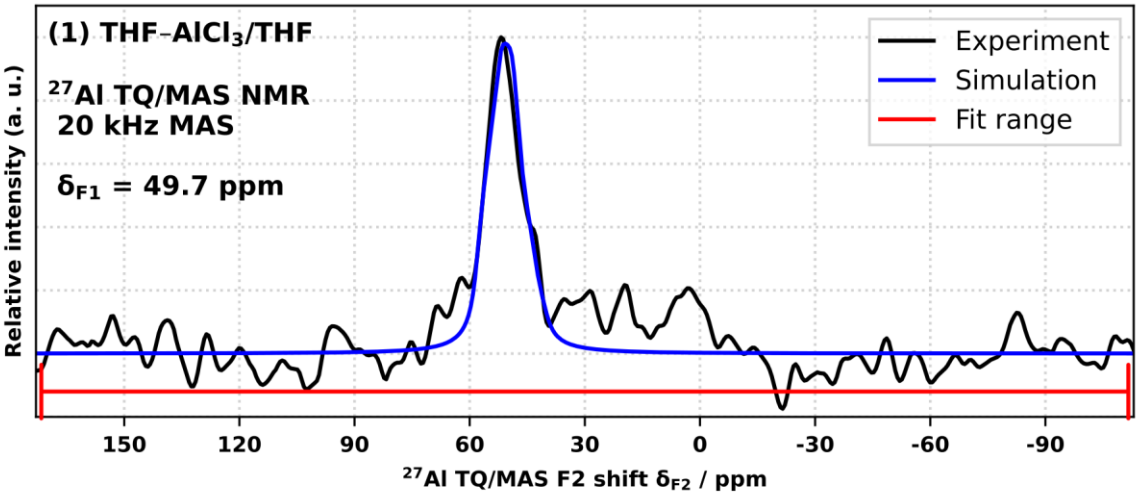

	49.731	12.025	0.100 (fixed)	500

C

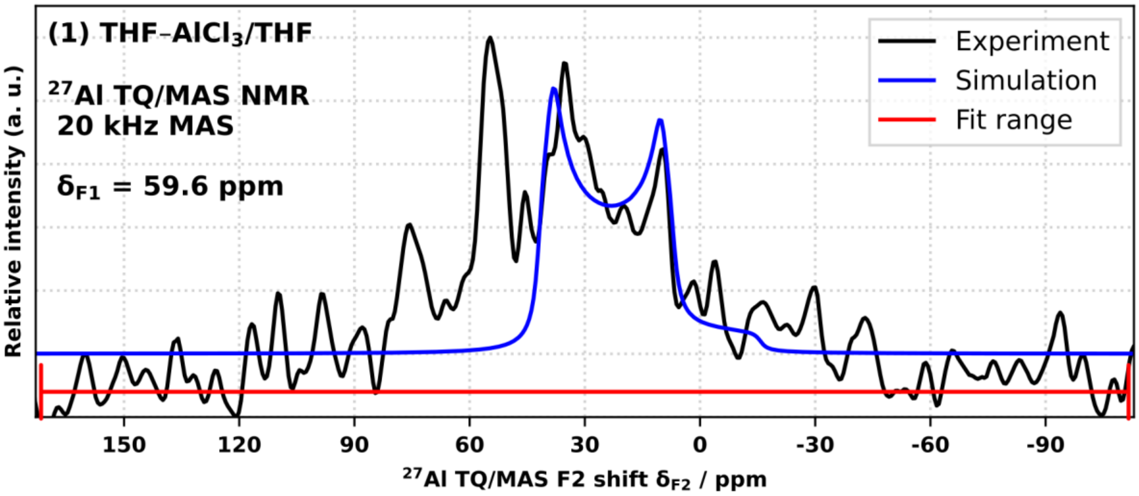

Product 2 (0.5 py–$AlCl_3$/THF)

Synthesis: $(Me_3Sn)_8Si_8O_{20}$ (2.0226 g, 1.0902 mmol), $AlCl_3$ (0.0704 g, 0.528 mmol), py (0.050 cm^3, 0.64 mmol), THF (solvent, 30 cm^3). **Byproducts:** 0.3687 g. $n(SnMe_4)/n(SnMe_3Cl)$ = 0.187206.

IR (KBr, cm^{-1}) *ν*: 513 w, 542 m, 583 w, 623 m, 664 vw, 698 vw, 724 vw, 778 m (ν SiOSi), 1035 vs ($ν_{as}$ SiOAl), 1139 vs ($ν_{as}$ SiOSi), 1402 vw ($δ_{as}$ CH_3), 1455 vw (ν py C–C/C–N), 1495 vw (ν py C–C/C–N), 1622 vw (ν py C–C/C–N), 2919 vw ($ν_s$ CH_3), 2990 vw ($ν_{as}$ CH_3), 3435 vw (ν O–H).

^{1}H MAS NMR *δ*: 8.8 (py *o*-Ar), 7.5 (py *m*-Ar), 0.1 (–OSn(CH_3)$_3$) ppm.

^{13}C TOSS NMR *δ*: 150.9 (py *o*-Ar), 128.9 (py *m*-Ar), 0.5 (–OSn(CH_3)$_3$) ppm.

^{29}Si MAS NMR *δ*: –101.9 ((CH_3)$_3$SnO*Si*(OSi)$_3$), –107.8 (AlO*Si*(OSi)$_3$) ppm.

Composition: ICP-OES: 0.846 wt% Al, 49.0 wt% Sn; **Gravimetry:** 0.807 wt% Al, 46.0 wt% Sn.

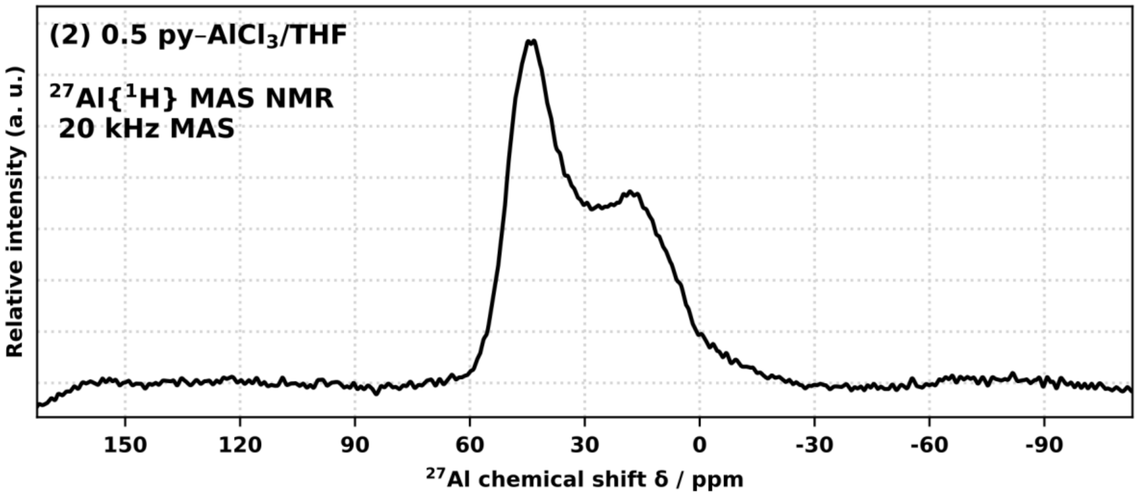

Figure S3. ^{27}Al{^{1}H} MAS NMR spectrum of product **2** (20 kHz MAS).

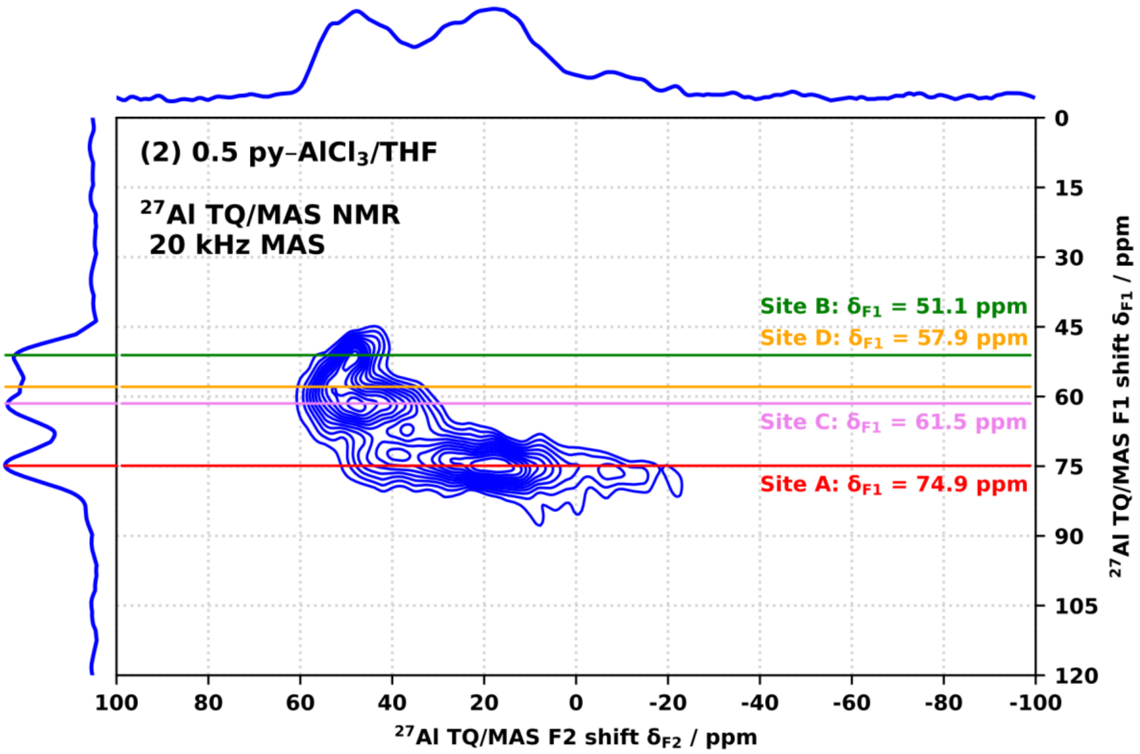

Figure S4. ^{27}Al TQ/MAS NMR spectrum of product **2** (20 kHz MAS).

Table S3. Slices through the ^{27}Al TQ/MAS NMR spectrum of product **2** and the corresponding line shape simulation fits.

Site	^{27}Al line shape simulation fit (quadrupole central transition)			
	δ_{iso} (ppm)	C_Q (MHz)	η	*LB* (fixed)
	49.866	11.798	0.745	1000
A	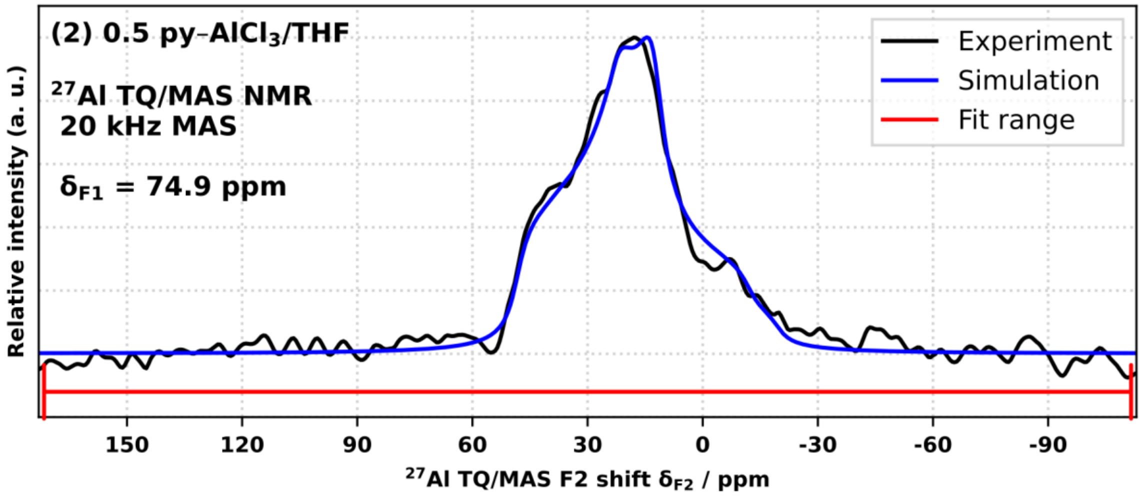			

	52.180	4.814	0.526	600

B

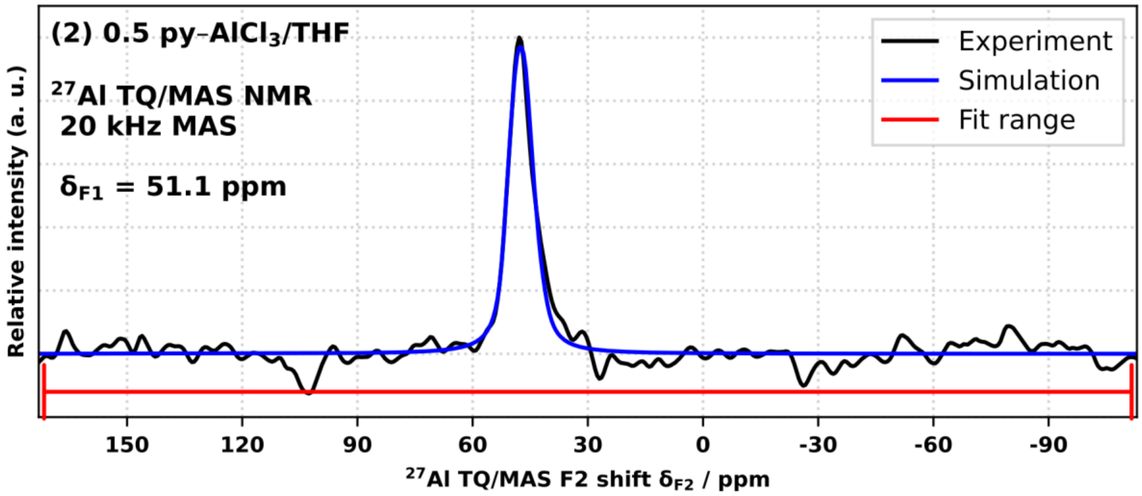

	57.981	9.002	0.424	600

C

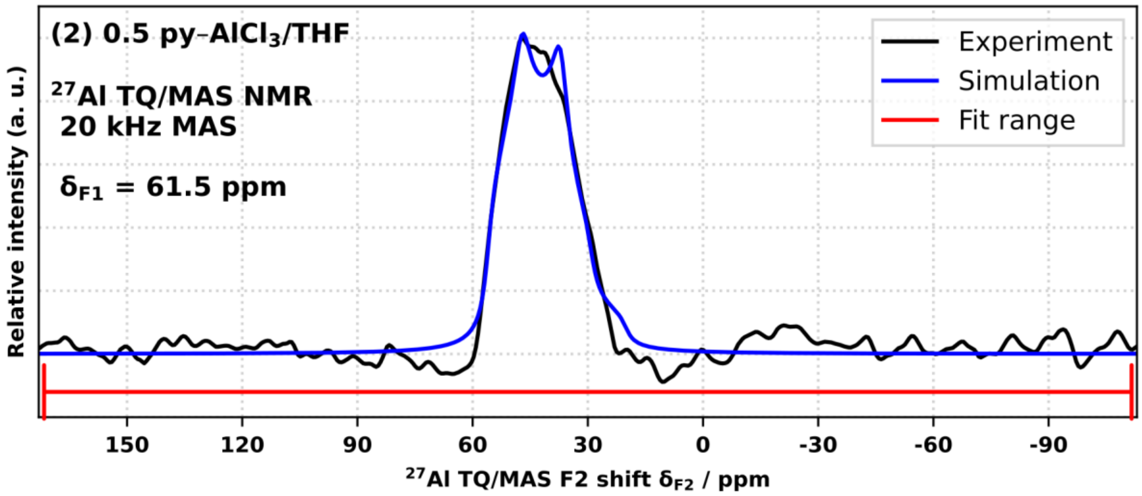

	58.605	6.296	0.426	600

D

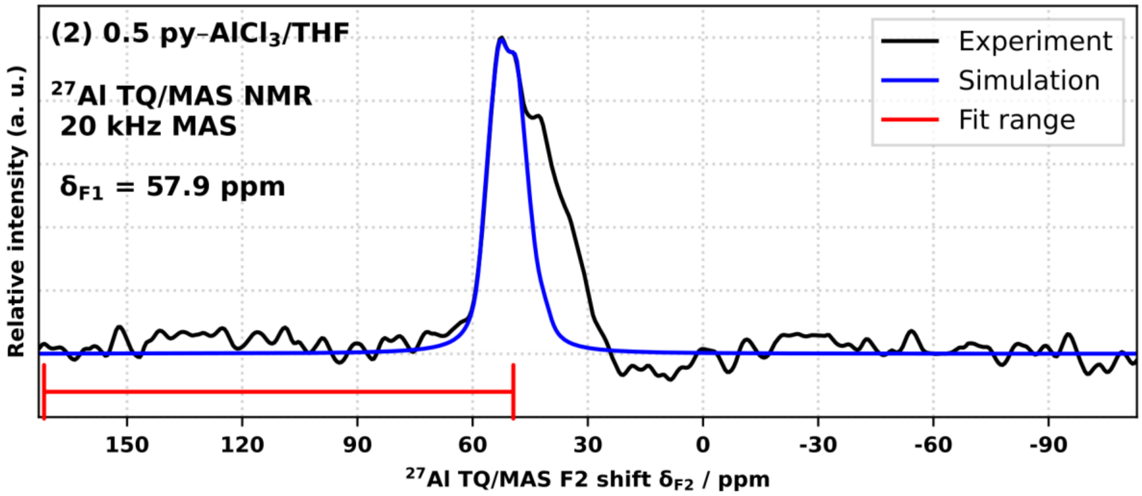

Product 3 (py–$AlCl_3$/THF)

Synthesis: $(Me_3Sn)_8Si_8O_{20}$ (1.9385 g, 1.0449 mmol), $AlCl_3$ (0.1349 g, 1.012 mmol), py (0.100 cm^3; 1.24 mmol), THF (solvent, 30 cm^3). **Byproducts:** 0.6363 g, *n($SnMe_4$)/n($SnMe_3Cl$)* = 0.023025.

IR (KBr, cm^{-1}) *v*: 513 w, 542 m, 583 w, 623 m, 664 vw, 698 vw, 724 vw, 778 m (v SiOSi), 1035 vs (v_{as} SiOAl), 1136 vs (v_{as} SiOSi), 1402 vw (δ_{as} CH_3), 1455 vw (v py C–C/C–N), 1495 vw (v py C–C/C–N), 1622 vw (v py C–C/C–N), 2919 vw (v_s CH_3), 2990 vw (v_{as} CH_3), 3435 vw (v O–H).

^{13}C TOSS NMR *δ*: 149.3 (py *o*-Ar), 127.0 (py *m*-Ar), –1.7 (–OSn(CH_3)$_3$) ppm.

^{29}Si MAS NMR *δ*: –103.1 ((CH_3)$_3$SnO*Si*(OSi)$_3$), –109.3 (AlO*Si*(OSi)$_3$) ppm.

Composition: ICP-OES: 1.83 wt% Al, 41.1 wt% Sn; **Gravimetry:** 1.80 wt% Al, 40.4 wt% Sn.

N_2 ads.: non-porous, V_{tot} 0.0160 cm^3 g^{-1}, Type III isotherm.

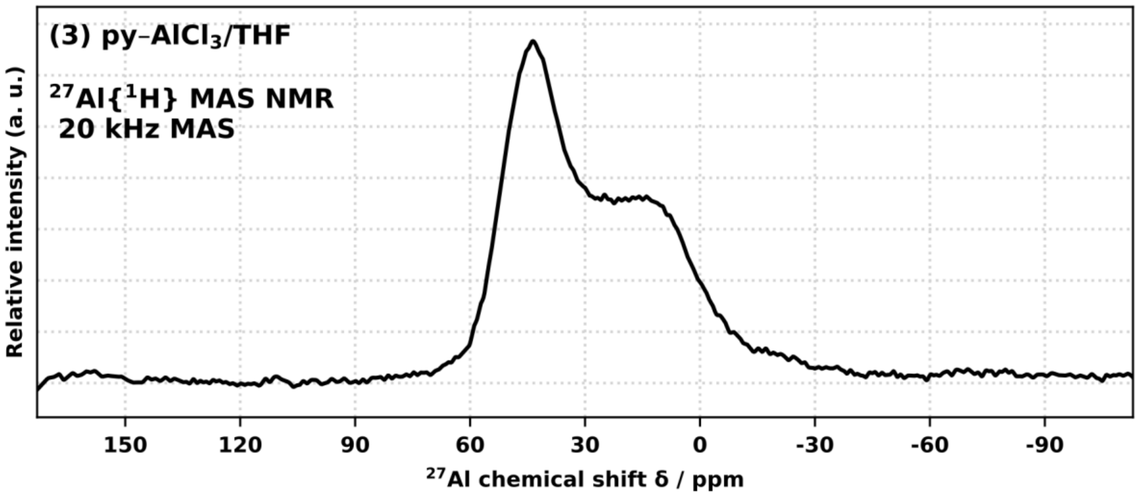

Figure S5. ^{27}Al{^{1}H} MAS NMR spectrum of product **3** (20 kHz MAS).

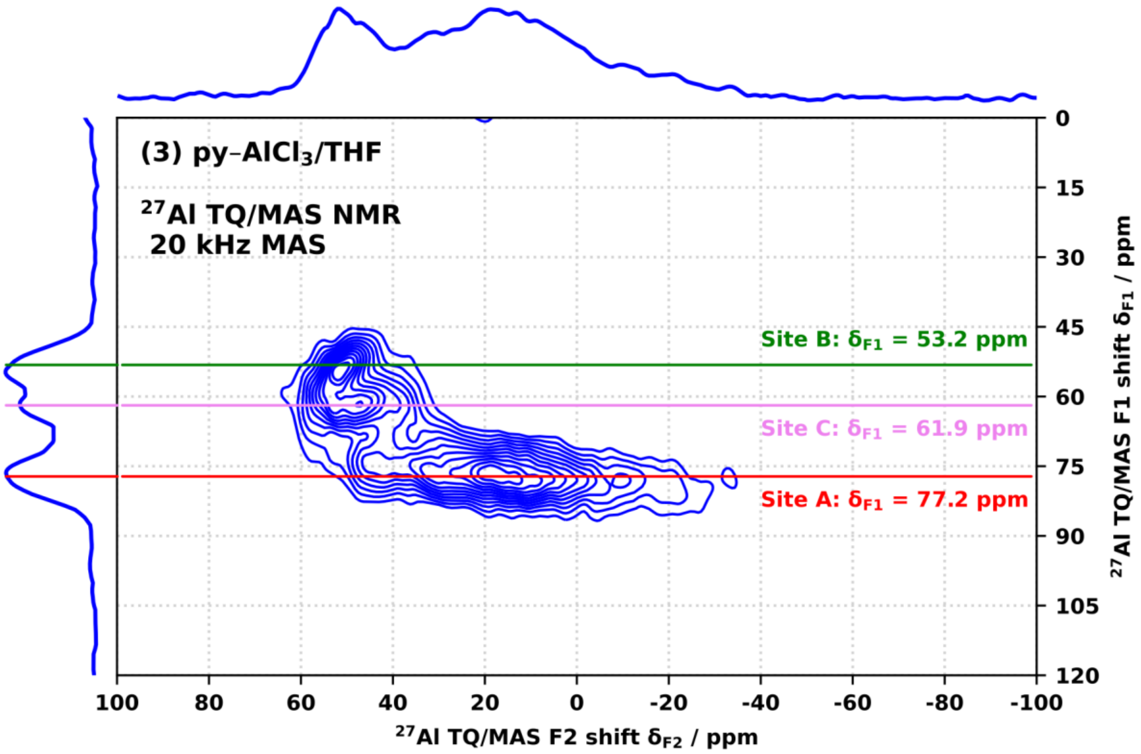

Figure S6. ^{27}Al TQ/MAS NMR spectrum of product **3** (20 kHz MAS).

Table S4. Slices through the ^{27}Al TQ/MAS NMR spectrum of product **3** and the corresponding line shape simulation fits.

**Site**	**^{27}Al line shape simulation fit (quadrupole central transition)**			
	**δ_{iso} (ppm)**	**C_Q (MHz)**	**η**	***LB* (fixed)**
	51.799	13.320	0.670	1000
**A**	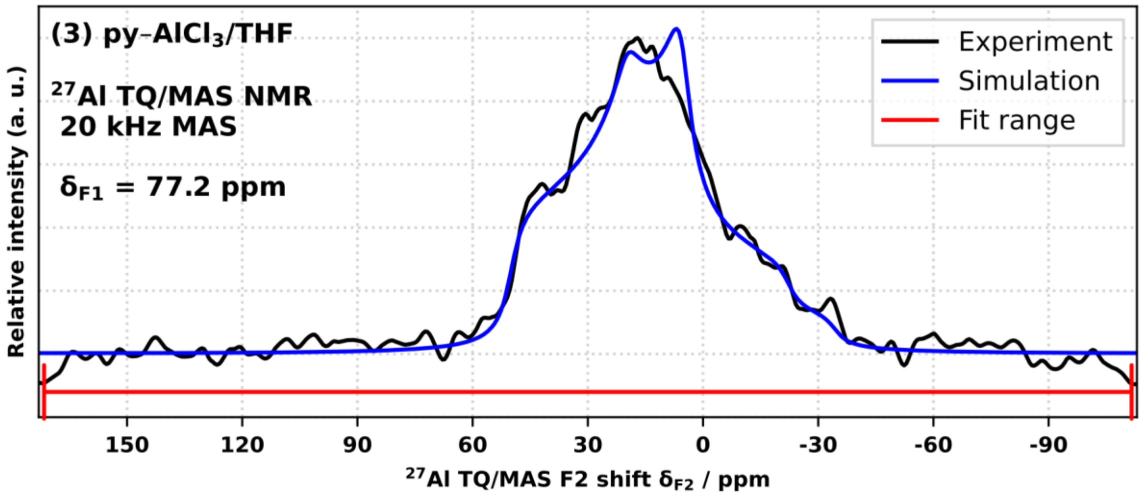			

58.552	6.195	0.690	600

B

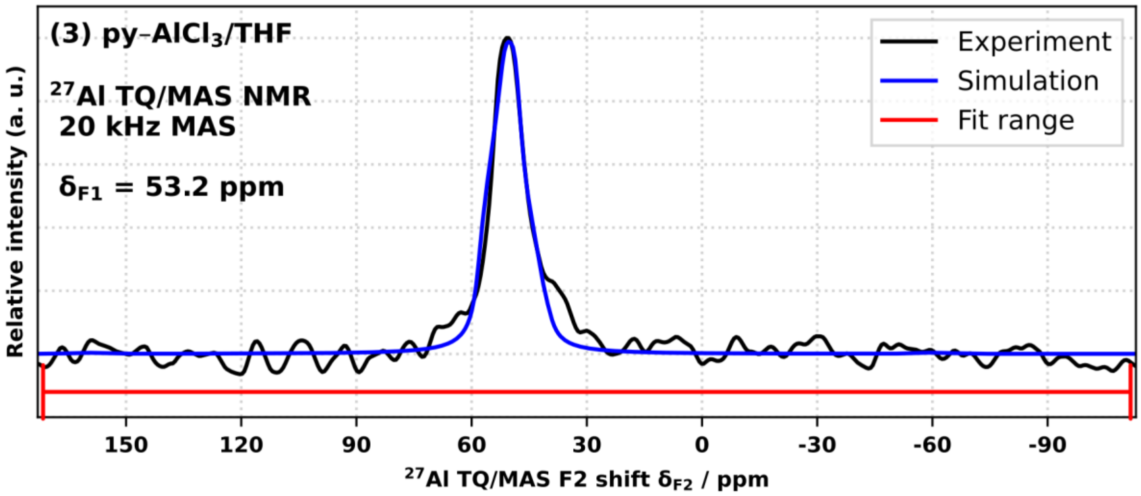

61.724	8.918	0.429	600

C

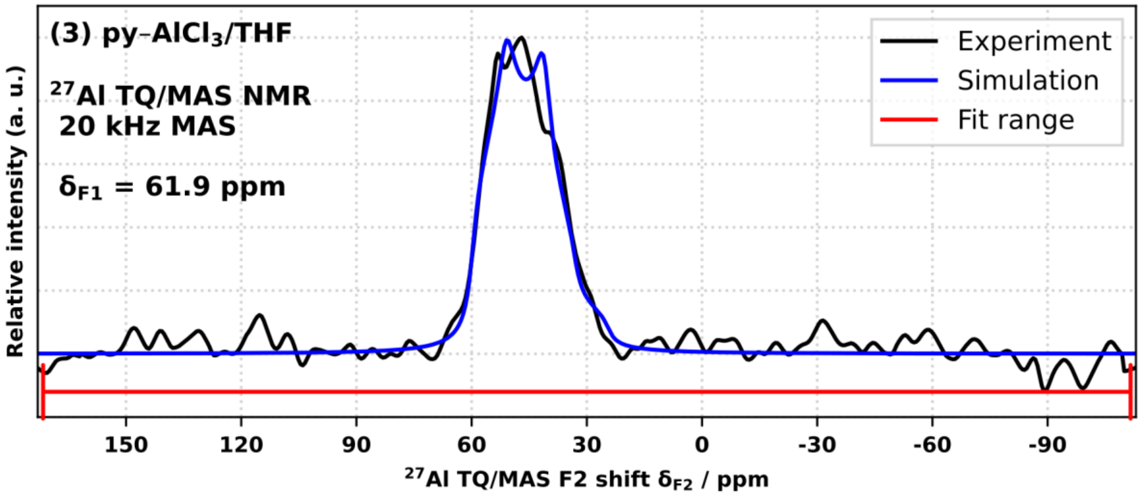

Product 4 (0.5 py–AlMe$_3$/toluene)

Synthesis: $(Me_3Sn)_8Si_8O_{20}$ (2.0629 g, 1.1120 mmol), py–AlMe$_3$ (0.0826 g, 0.546 mmol), toluene (solvent, 30 cm^3). **Byproducts:** 0.3242 g ($SnMe_4$ only).

IR (KBr, cm^{-1}) *ν*: 513 w, 542 m, 583 w, 623 m, 664 vw, 698 vw, 724 vw, 778 m (ν SiOSi), 1035 vs ($ν_{as}$ SiOAl), 1139 vs ($ν_{as}$ SiOSi), 1402 vw ($δ_{as}$ CH_3), 1455 vw (ν py C–C/C–N), 1495 vw (ν py C–C/C–N), 1622 vw (ν py C–C/C–N), 2919 vw ($ν_s$ CH_3), 2990 vw ($ν_{as}$ CH_3), 3435 vw (ν O–H).

^{1}H MAS NMR *δ*: 8.7 (py *o*-Ar), 7.5 (py *m*-Ar), 0.1 (–OSn(C*H*$_3$)$_3$) ppm.

^{13}C TOSS NMR *δ*: 151.4 (py *o*-Ar), 128.7 (py *m*-Ar), 0.5 (–OSn(*C*H$_3$)$_3$) ppm.

^{29}Si MAS NMR *δ*: –101.8 ((CH$_3$)$_3$SnO*Si*(OSi)$_3$), –107.9 (AlO*Si*(OSi)$_3$) ppm.

Composition: ICP-OES: 0.883 wt% Al, 48.9 wt% Sn; **Gravimetry:** 0.809 wt% Al, 46.2 wt% Sn.

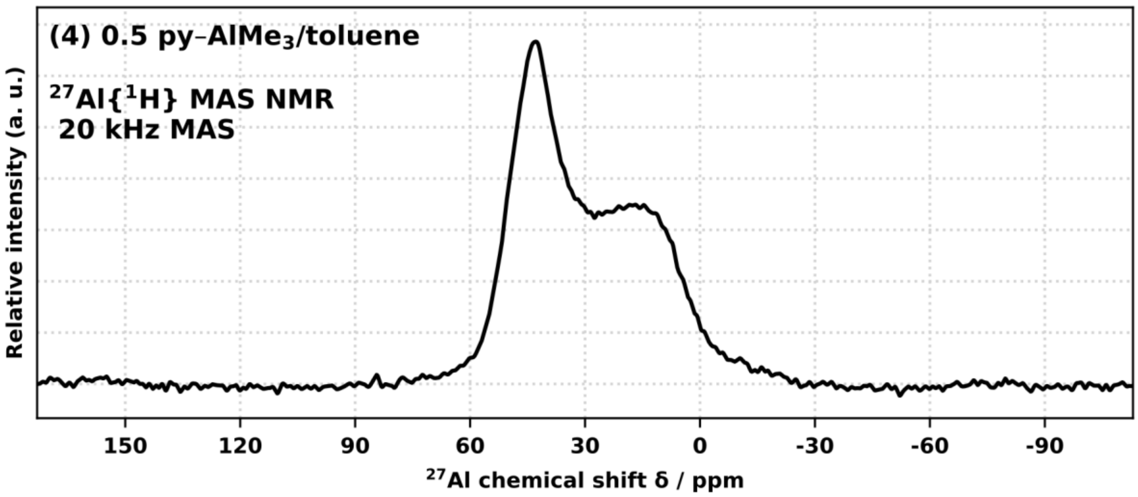

Figure S7. ^{27}Al{^{1}H} MAS NMR spectrum of product **4** (20 kHz MAS).

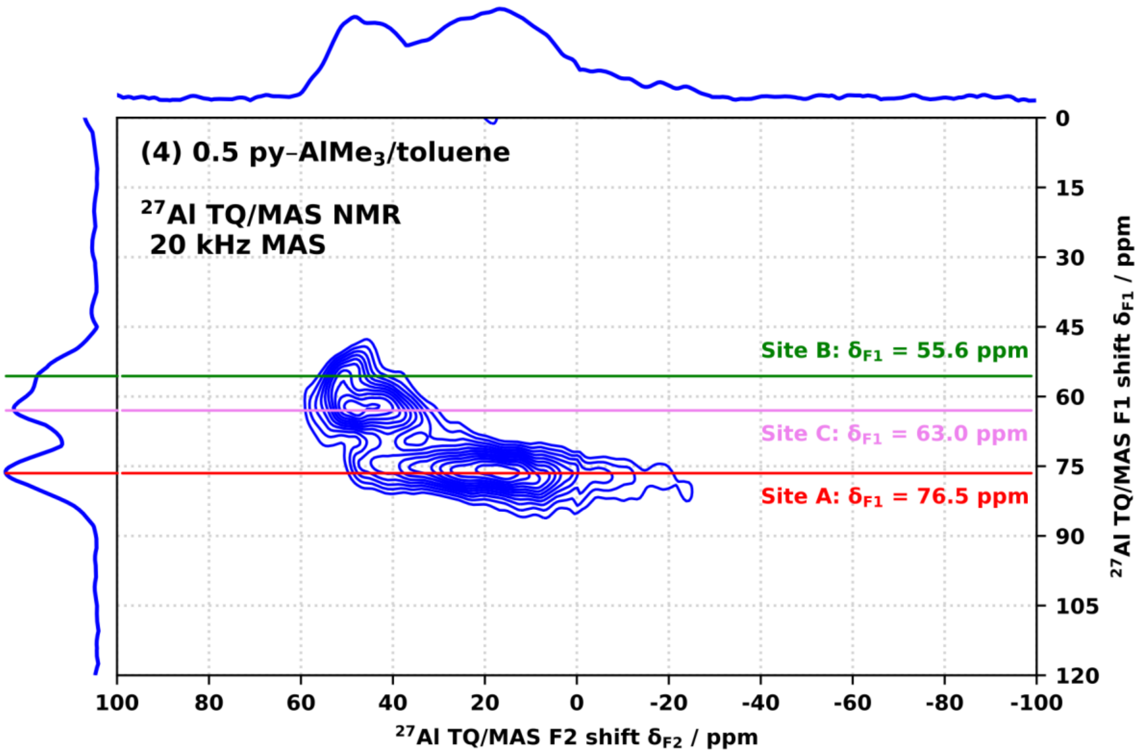

Figure S8. ^{27}Al TQ/MAS NMR spectrum of product **4** (20 kHz MAS).

Table S5. Slices through the ^{27}Al TQ/MAS NMR spectrum of product **4** and the corresponding line shape simulation fits.

Site	^{27}Al line shape simulation fit (quadrupole central transition)			
	δ_{iso} (ppm)	C_Q (MHz)	η	*LB* (fixed)
	49.319	12.282	0.630	1000

A

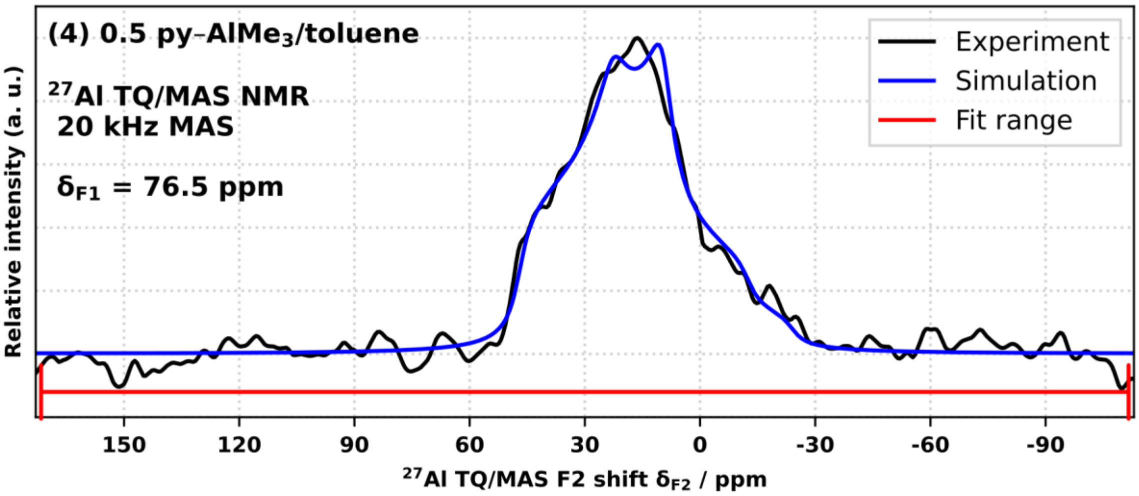

55.418	5.923	0.364	500

B

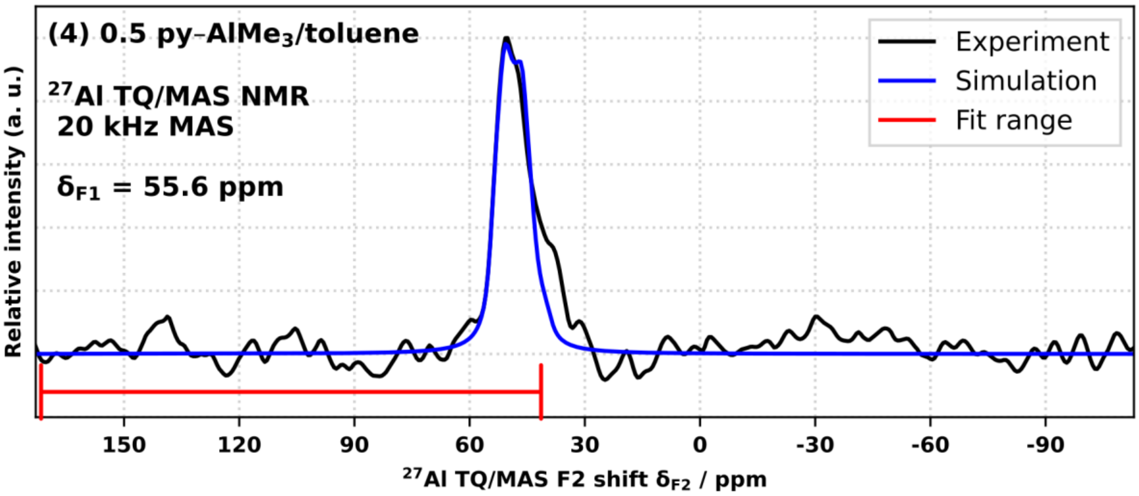

56.218	8.395	0.434	600

C

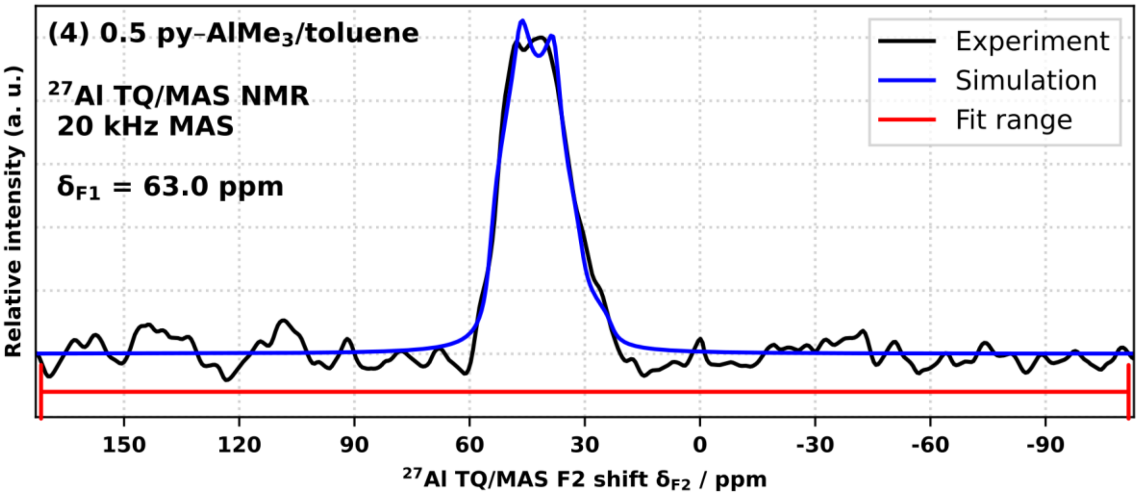

Product 5 (py–AlMe$_3$/toluene)

Synthesis: $(Me_3Sn)_8Si_8O_{20}$ (1.8221 g, 0.9822 mmol), py–AlMe$_3$ (0.1500 g, 0.9922 mmol), toluene (solvent, 35 cm^3). **Byproducts:** 0.5446 g ($SnMe_4$ only).

IR (KBr, cm^{-1}) *ν*: 513 w, 542 m, 583 w, 623 m, 664 vw, 698 vw, 724 vw, 778 m (ν SiOSi), 1040 vs ($ν_{as}$ SiOAl), 1136 vs ($ν_{as}$ SiOSi), 1402 vw ($δ_{as}$ CH_3), 1455 vw (ν py C–C/C–N), 1495 vw (ν py C–C/C–N), 1622 vw (ν py C–C/C–N), 2919 vw ($ν_s$ CH_3), 2990 vw ($ν_{as}$ CH_3), 3435 vw (ν O–H).

^{13}C TOSS NMR *δ*: 155.9 (s, *p*-Ar), 152.2 (py *o*-Ar), 129.0 (py *m*-Ar), 0.5 (–OSn(CH_3)$_3$) ppm.

^{29}Si MAS NMR *δ*: –101.2 ((CH_3)$_3$SnO*Si*(OSi)$_3$), –107.4 (AlO*Si*(OSi)$_3$) ppm.

Composition: ICP-OES: 1.95 wt% Al, 42.4 wt% Sn; **Gravimetry:** 1.88 wt% Al, 40.0 wt% Sn.

N_2 ads.: non-porous, V_{tot} 0.00864 cm^3 g^{-1}, Type III isotherm.

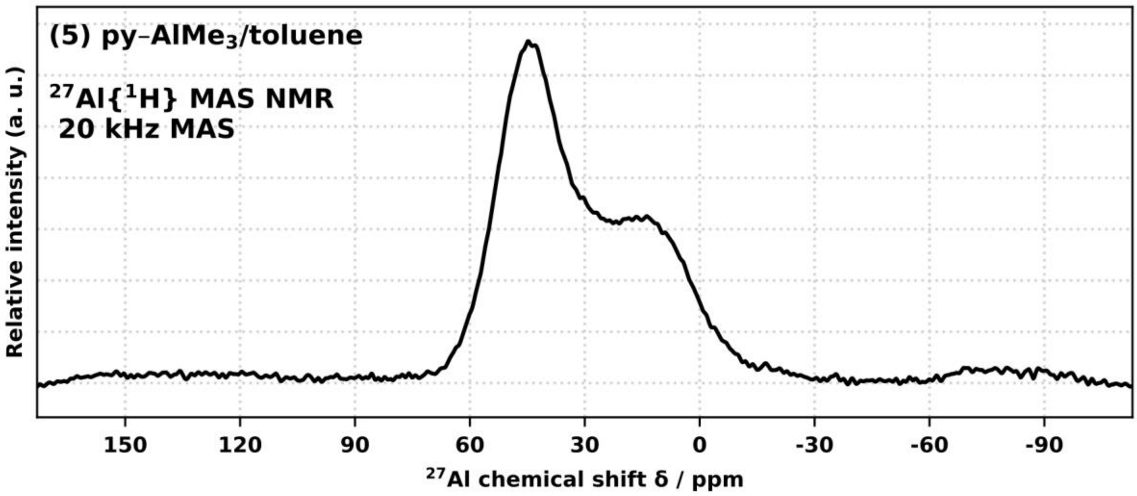

Figure S9. ^{27}Al{^{1}H} MAS NMR spectrum of product **5** (20 kHz MAS).

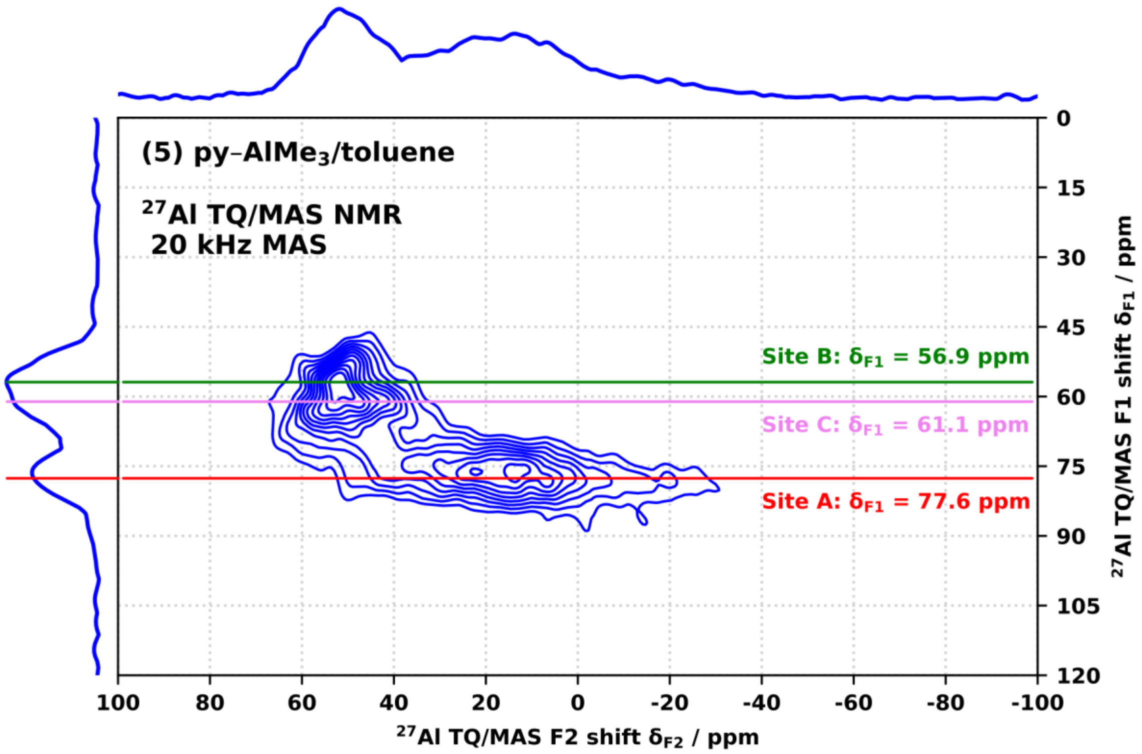

Figure S10. ^{27}Al TQ/MAS NMR spectrum of product **5** (20 kHz MAS).

Table S6. Slices through the ^{27}Al TQ/MAS NMR spectrum of product **5** and the corresponding line shape simulation fits.

Site	^{27}Al line shape simulation fit (quadrupole central transition)			
	δ_{iso} (ppm)	C_Q (MHz)	η	*LB* (fixed)
	55.077	14.052	0.652	1000
A	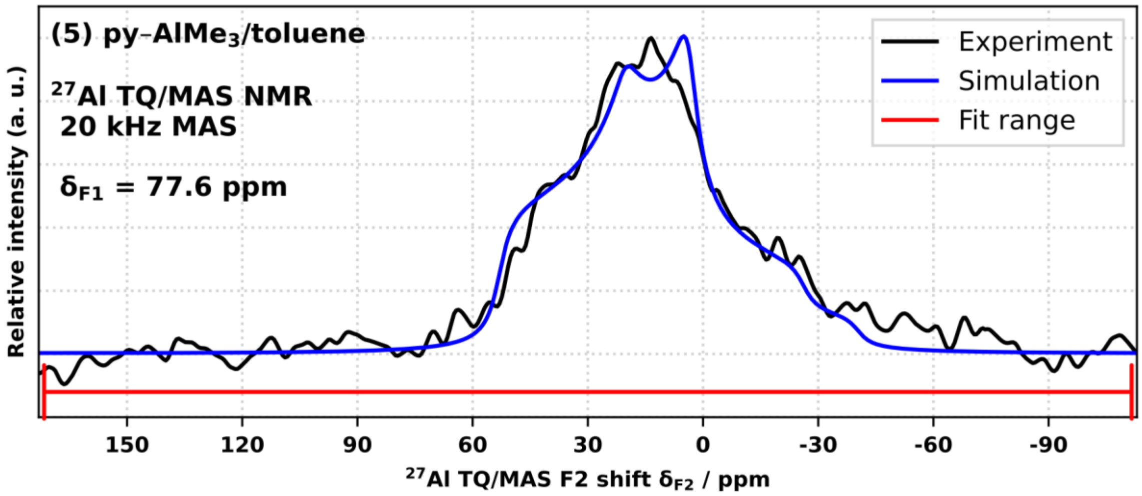			

59.205	6.281	0.532	1000

B

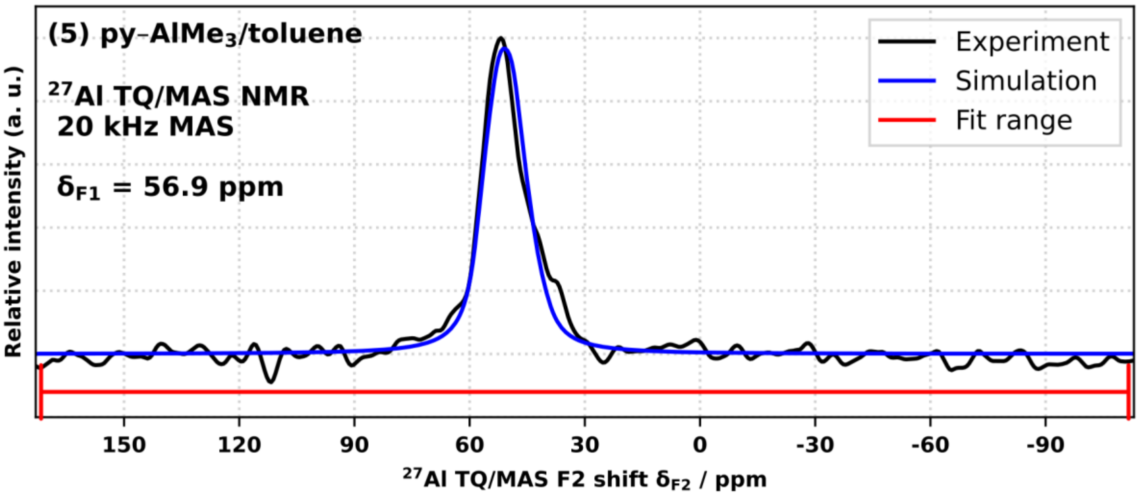

63.151	8.580	0.450	600

C

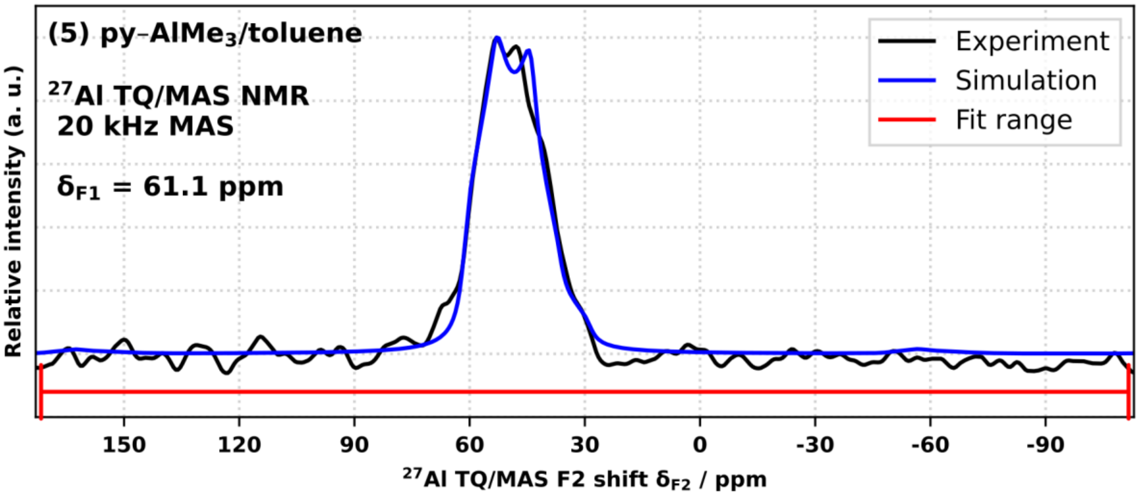

Product 6 (py–$AlMe_3$/toluene/100 °C)

Synthesis: $(Me_3Sn)_8Si_8O_{20}$ (2.1225 g, 1.1441 mmol), py–$AlMe_3$ (0.1707 g, 1.129 mmol), toluene (solvent, 30 cm^3). Heated at 100 °C after warm-up to r.t. The product is a clear gel. **Byproducts:** 0.7248 g ($SnMe_4$ only).

IR (KBr, cm^{-1}) *ν*: 513 w, 542 m, 583 w, 623 m, 664 vw, 698 vw, 724 vw, 778 m (ν SiOSi), 1045 vs (ν_{as} SiOAl), 1136 vs (ν_{as} SiOSi), 1402 vw (δ_{as} CH_3), 1455 vw (ν py C–C/C–N), 1495 vw (ν py C–C/C–N), 1622 vw (ν py C–C/C–N), 2919 vw (ν_s CH_3), 2990 vw (ν_{as} CH_3), 3435 vw (ν O–H).

^{1}H MAS NMR *δ*: 8.1 (py *o*-Ar), 6.8 (py *m*-Ar), –0.5 (–OSn(CH_3)$_3$) ppm.

^{13}C TOSS NMR *δ*: 151.6 (py *o*-Ar), 129.5 (py *m*-Ar), 0.8 (–OSn(CH_3)$_3$) ppm.

^{29}Si MAS NMR *δ*: –101.2 ((CH_3)$_3$SnO*Si*(OSi)$_3$), –106.6 (AlO*Si*(OSi)$_3$) ppm.

Composition: ICP-OES: 2.07 wt% Al, 40.9 wt% Sn; **Gravimetry:** 1.94 wt% Al, 38.6 wt% Sn.

N_2 ads.: SA_{BET} 126 m^2 g^{-1}, C_{BET} 52.6, V_{tot} 0.0878 cm^3 g^{-1}, Type I isotherm, Type H4 open-loop hysteresis.

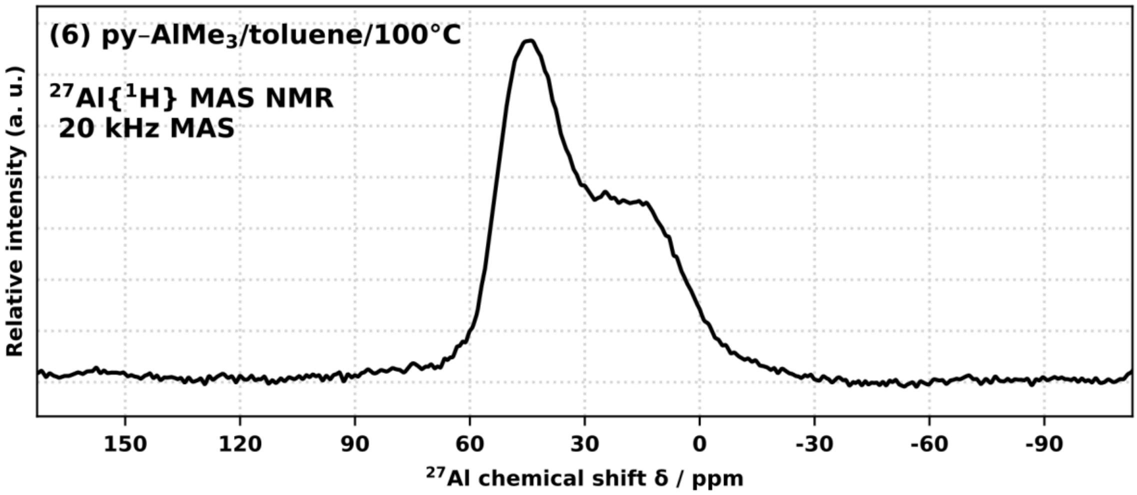

Figure S11. ^{27}Al{^{1}H} MAS NMR spectrum of product **6** (20 kHz MAS).

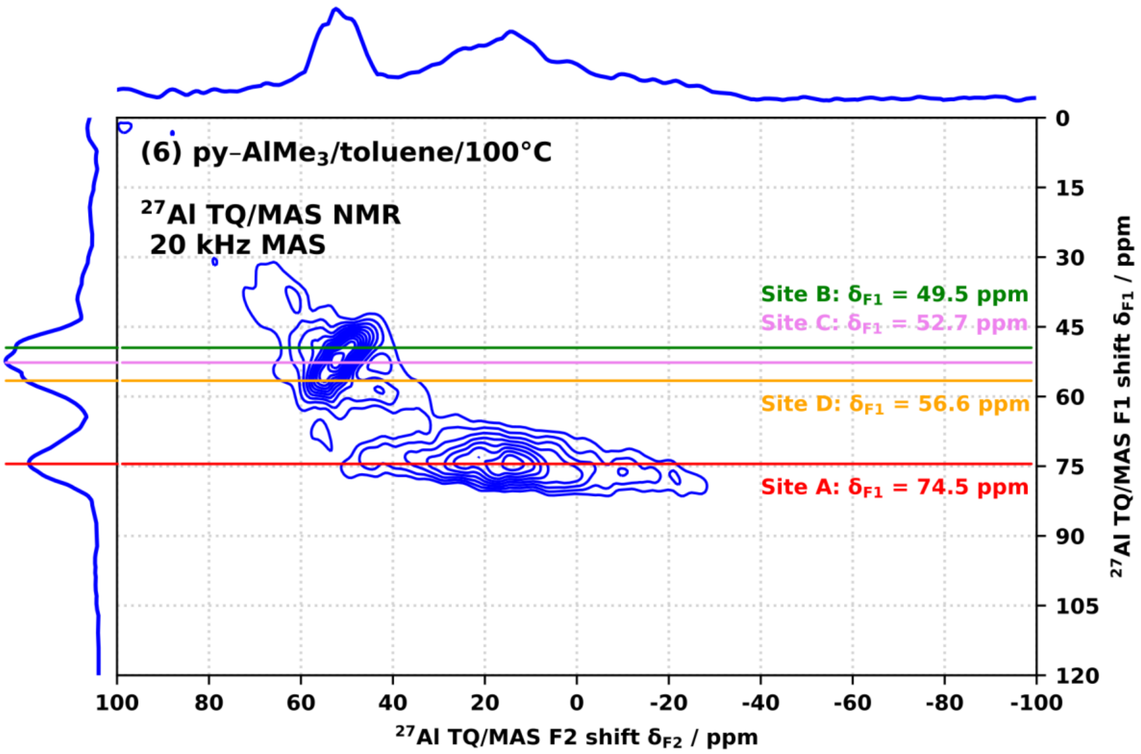

Figure S12. ^{27}Al TQ/MAS NMR spectrum of product **6** (20 kHz MAS).

Table S7. Slices through the ^{27}Al TQ/MAS NMR spectrum of product **6** and the corresponding line shape simulation fits.

Site	^{27}Al line shape simulation fit (quadrupole central transition)			
	δ_{iso} (ppm)	C_Q (MHz)	*η*	*LB* (fixed)
	51.851	12.917	0.772	1000
A	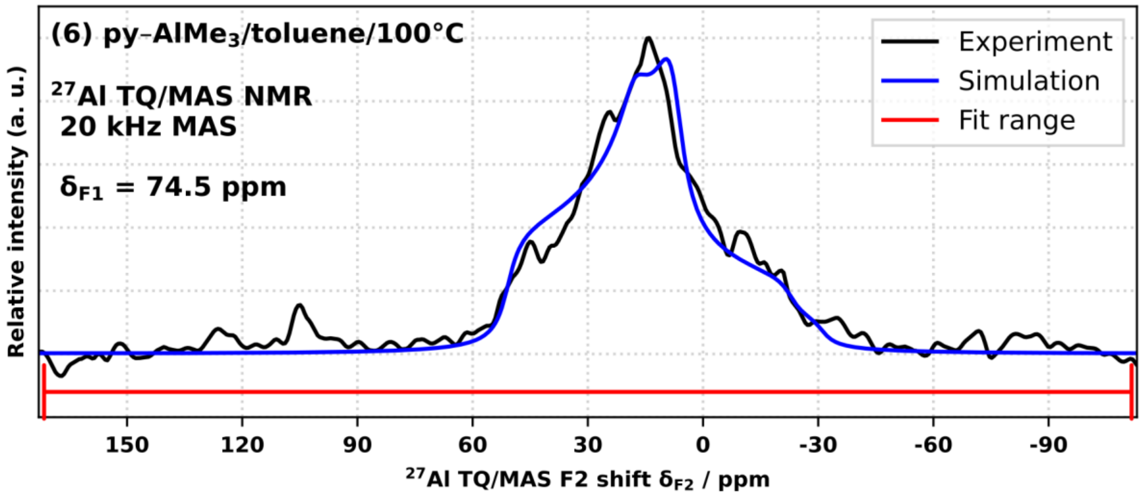			

56.519	5.591	0.650	800

B

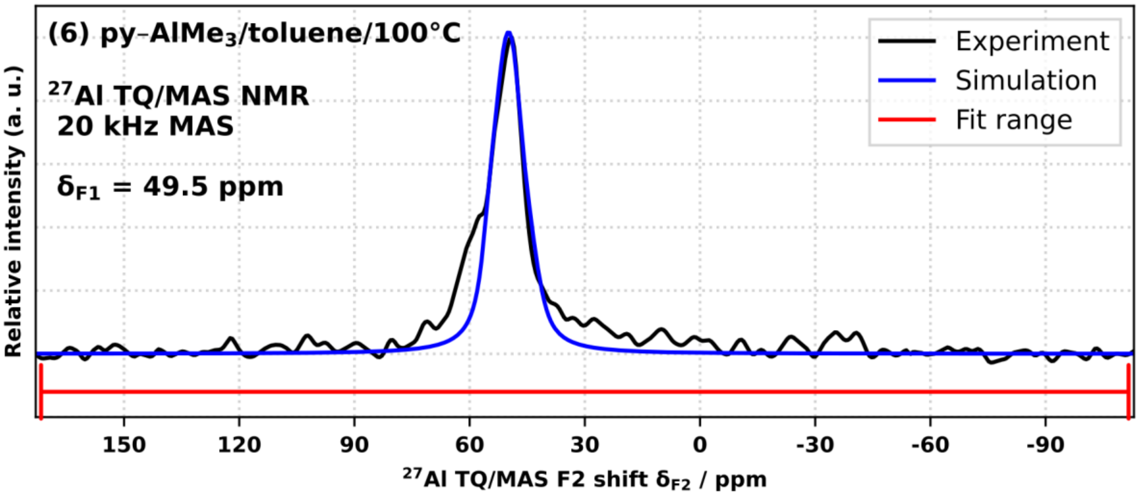

58.585	5.161	0.821	600

C

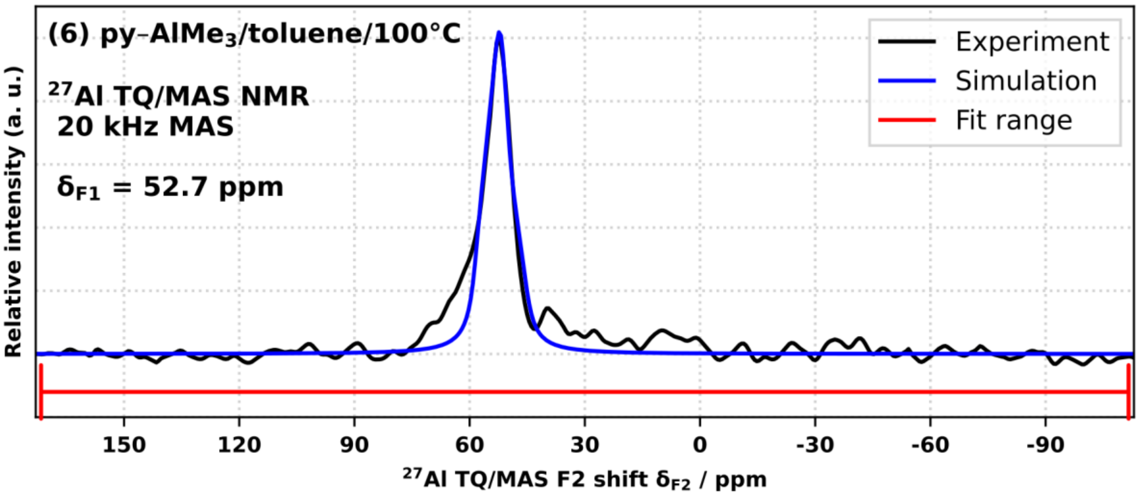

59.466	4.921	0.549	800

D

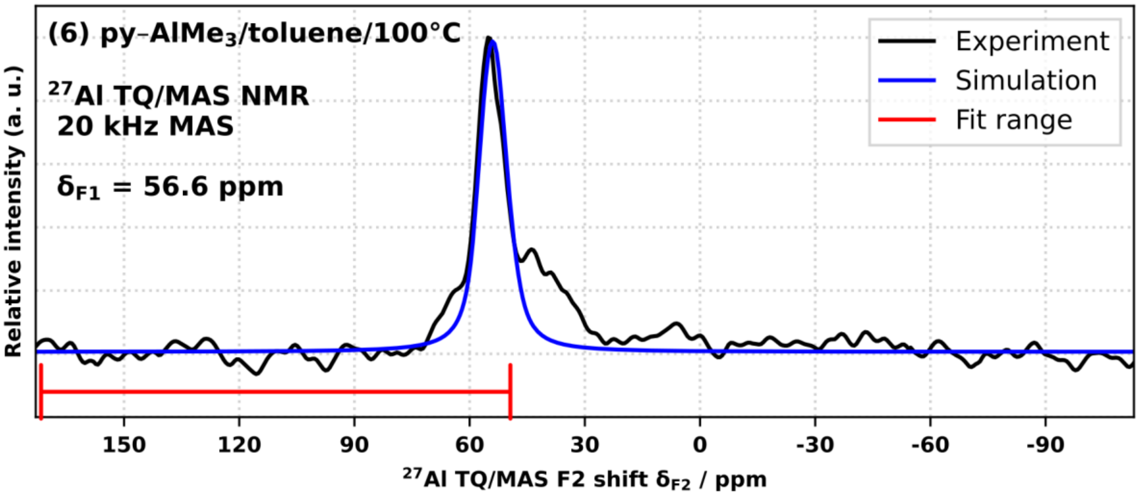

Product 7 (0.5 py–AlMe$_3$/THF)

Synthesis: $(Me_3Sn)_8Si_8O_{20}$ (2.0560 g, 1.1082 mmol), py–AlMe$_3$ (0.0814 g, 0.538 mmol), THF (solvent, 30 cm^3). **Byproducts:** 0.3488 g ($SnMe_4$ only).

IR (KBr, cm^{-1}) *v*: 513 w, 542 m, 623 m, 664 vw, 698 vw, 724 vw, 778 m (v SiOSi), 1035 vs (v_{as} SiOAl), 1139 vs (v_{as} SiOSi), 1402 vw (δ_{as} CH_3), 1455 vw (v py C–C/C–N), 1495 vw (v py C–C/C–N), 1622 vw (v py C–C/C–N), 2919 vw (v_s CH_3), 2990 vw (v_{as} CH_3), 3435 vw (v O–H).

^{1}H MAS NMR *δ*: 8.8 (py *o*-Ar), 8.3 (py *p*-Ar), 7.6 (py *m*-Ar), 0.3 (–OSn(C*H*$_3$)$_3$) ppm.

^{13}C TOSS NMR *δ*: 151.3 (py *o*-Ar), 129.2 (py *m*-Ar), 0.7 (–OSn(*C*H$_3$)$_3$) ppm.

^{29}Si MAS NMR *δ*: –101.2 ((CH$_3$)$_3$SnO*Si*(OSi)$_3$), –107.2 (AlO*Si*(OSi)$_3$) ppm.

Composition: ICP-OES: 0.890 wt% Al, 51.0 wt% Sn; **Gravimetry:** 0.812 wt% Al, 45.9 wt% Sn.

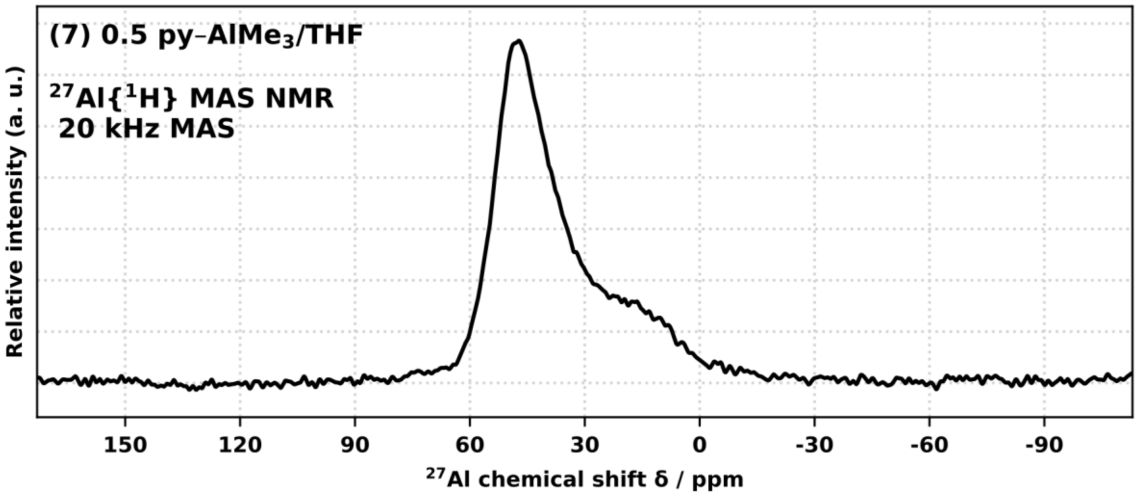

Figure S13. ^{27}Al{^{1}H} MAS NMR spectrum of product **7** (20 kHz MAS).

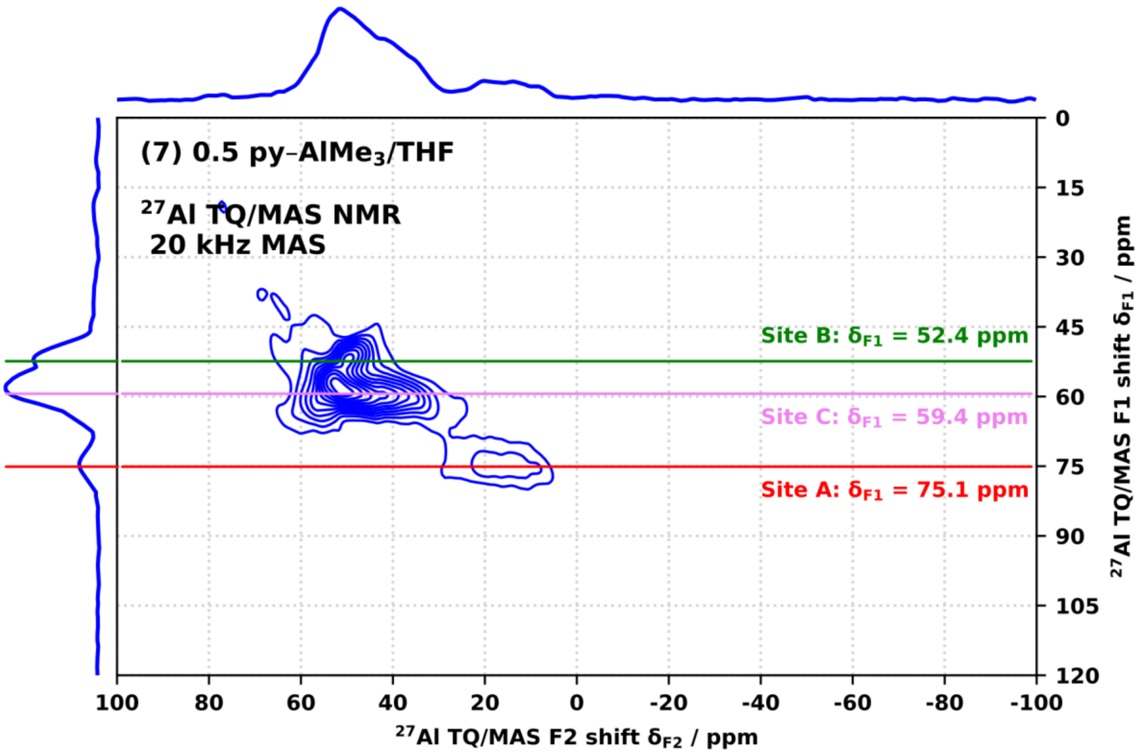

Figure S14. ^{27}Al TQ/MAS NMR spectrum of product **7** (20 kHz MAS).

Table S8. Slices through the ^{27}Al TQ/MAS NMR spectrum of product **7** and the corresponding line shape simulation fits.

Site	^{27}Al line shape simulation fit (quadrupole central transition)			
	δ_{iso} (ppm)	C_Q (MHz)	η	*LB* (fixed)
A	32.086	9.305	0.500 (fixed)	500

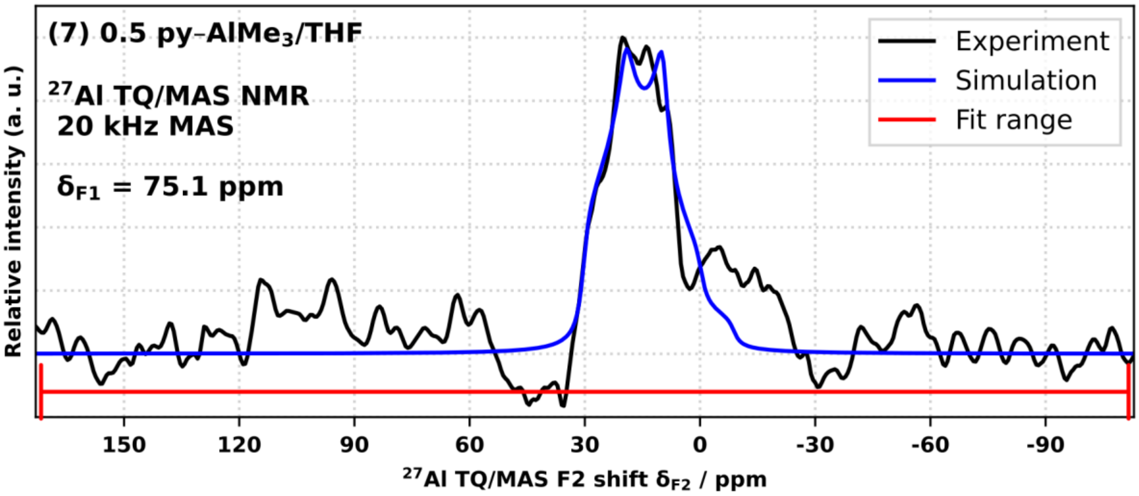

59.408	6.082	1.000 (fixed)	600

B

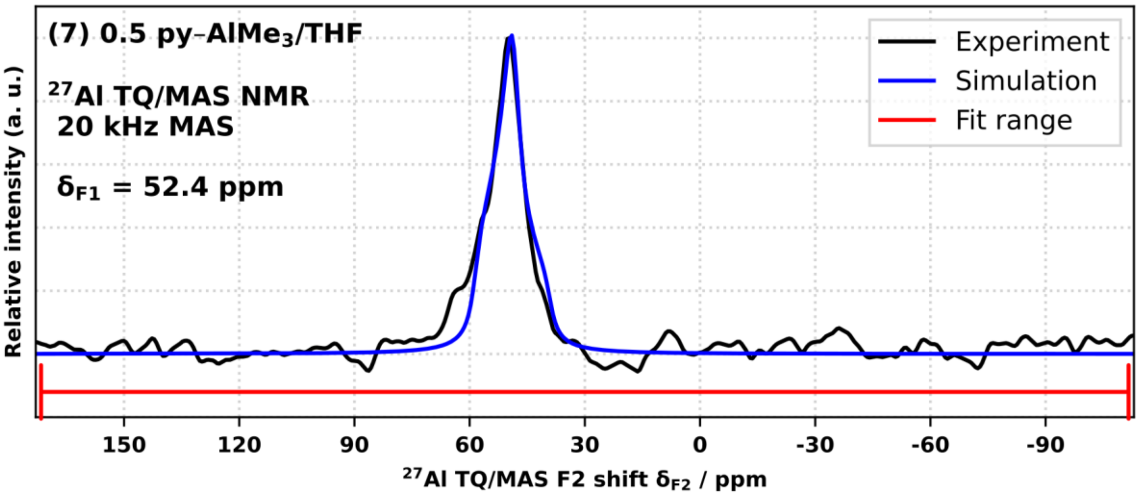

61.315	8.862	0.404	800

C

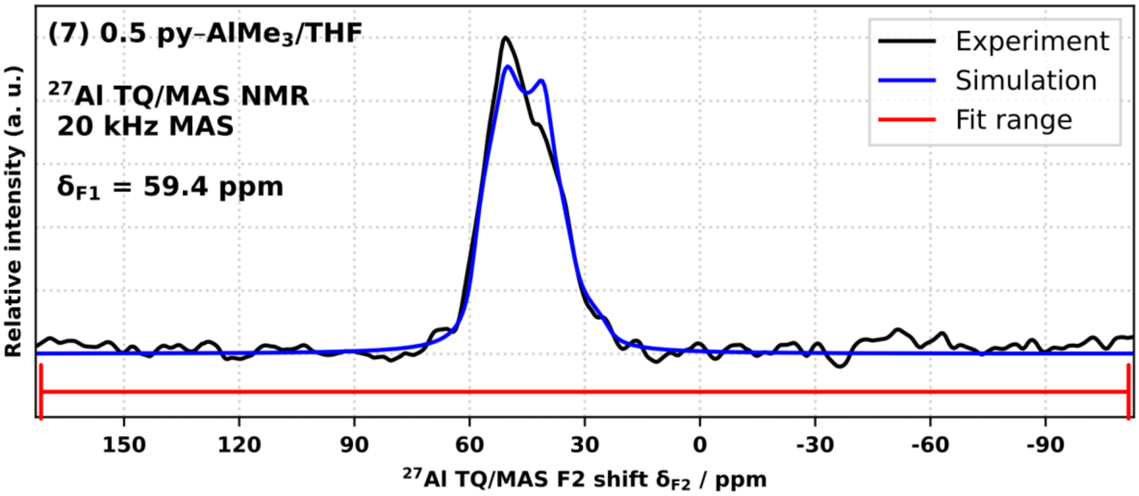

Product 8 (py–$AlMe_3$/THF)

Synthesis: $(Me_3Sn)_8Si_8O_{20}$ (2.0961 g, 1.1299 mmol), py–$AlMe_3$ (0.1689 g, 1.1172 mmol), THF (solvent, 30 cm^3). **Byproducts:** 0.6225 g ($SnMe_4$ only).

IR (KBr, cm^{-1}) *ν*: 513 w, 542 m, 583 w, 623 m, 664 vw, 698 vw, 724 vw, 778 m (ν SiOSi), 1040 vs ($ν_{as}$ SiOAl), 1136 vs ($ν_{as}$ SiOSi), 1402 vw ($δ_{as}$ CH_3), 1455 vw (ν py C–C/C–N), 1495 vw (ν py C–C/C–N), 1622 vw (ν py C–C/C–N), 2919 vw ($ν_s$ CH_3), 2990 vw ($ν_{as}$ CH_3), 3435 vw (ν O–H).

^{1}H MAS NMR *δ*: 8.6 (py *o*-Ar), 8.2 (py *p*-Ar), 7.5 (py *m*-Ar), 0.1 (–OSn(CH_3)$_3$) ppm.

^{13}C TOSS NMR *δ*: 151.3 (py *o*-Ar), 129.2 (py *m*-Ar), –0.8 (–OSn(CH_3)$_3$) ppm.

^{29}Si MAS NMR *δ*: –100.9 ((CH_3)$_3$SnO*Si*(OSi)$_3$), –106.9 (AlO*Si*(OSi)$_3$) ppm.

Composition: ICP-OES: 1.99 wt% Al, 42.6 wt% Sn; **Gravimetry:** 1.84 wt% Al, 40.2 wt% Sn.

N_2 ads.: non-porous, V_{tot} 0.00541 cm^3 g^{-1}, Type III isotherm.

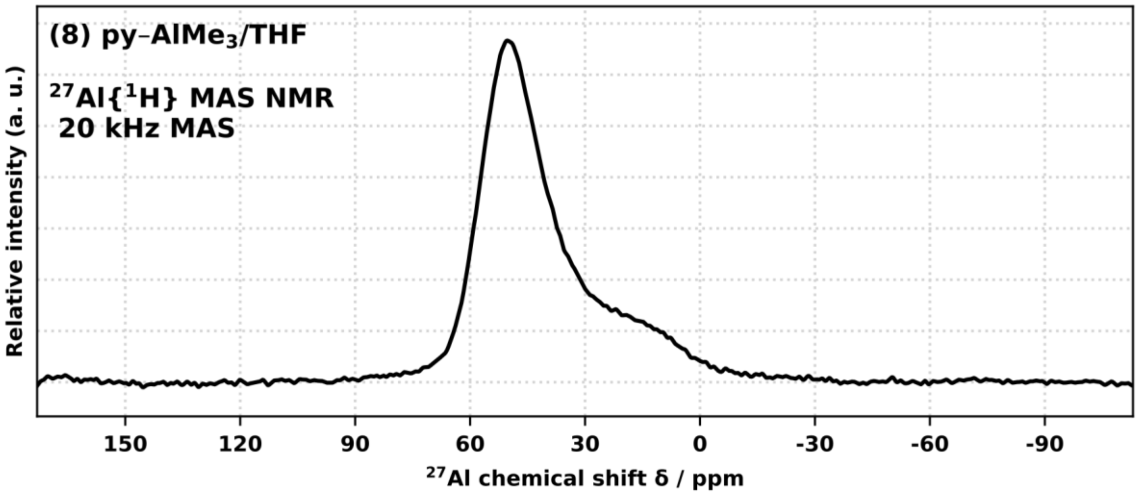

Figure S15. ^{27}Al{^{1}H} MAS NMR spectrum of product **8** (20 kHz MAS).

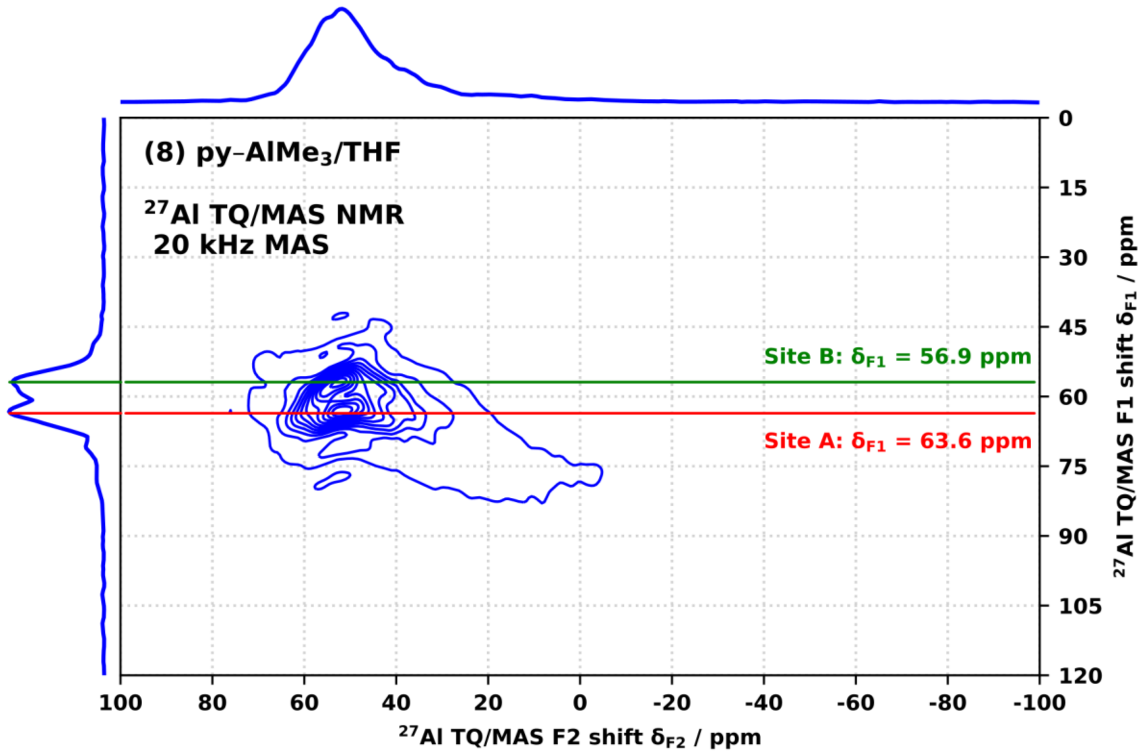

Figure S16. ^{27}Al TQ/MAS NMR spectrum of product **8** (20 kHz MAS).

Table S9. Slices through the ^{27}Al TQ/MAS NMR spectrum of product **8** and the corresponding line shape simulation fits.

Site	^{27}Al line shape simulation fit (quadrupole central transition)			
	δ_{iso} (ppm)	C_Q (MHz)	η	*LB* (fixed)
	64.351	8.015	0.674	600
A	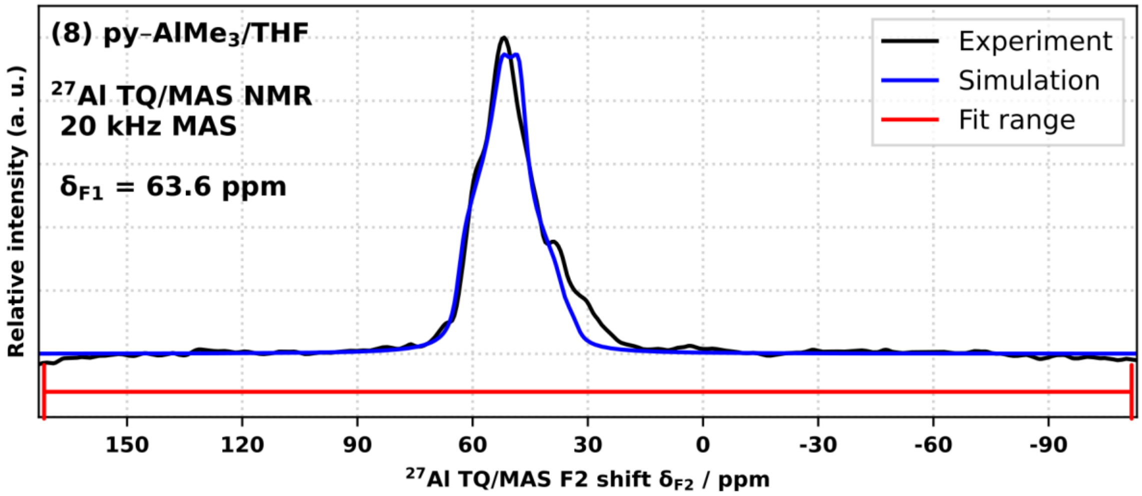			

57.282	5.804	0.351	800

B

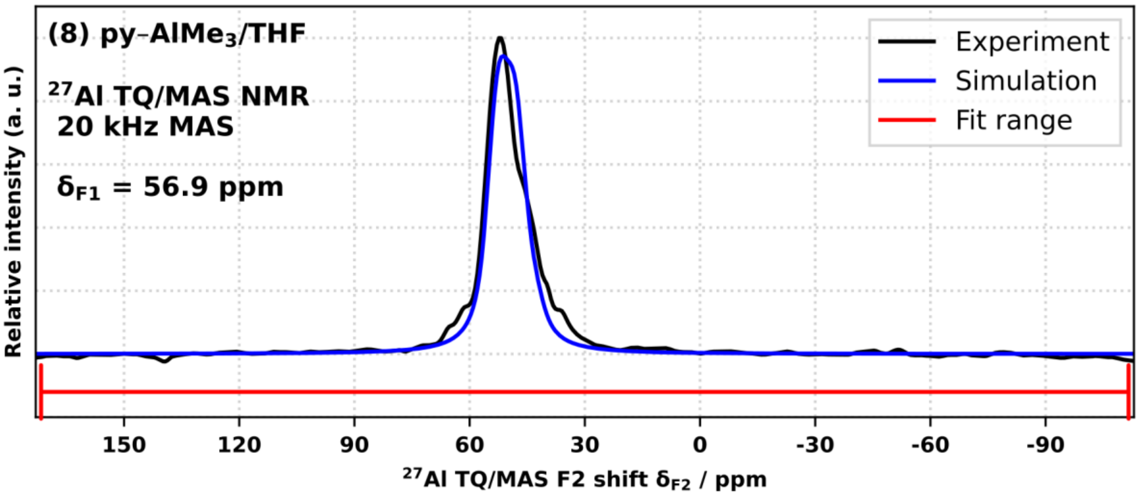

Product 9 (py–$AlEt_3$/toluene)

Synthesis: $(Me_3Sn)_8Si_8O_{20}$ (2.2554 g, 1.2157 mmol), py–$AlEt_3$ (0.2350 g, 1.216 mmol), toluene (solvent, 35 cm^3). **Byproducts:** 0.6943 g, *n($SnMe_4$)/n($SnMe_3Et$)* = 0.022275.

IR (KBr, cm^{-1}) *ν*: 513 w, 542 m, 583 w, 623 m, 664 vw, 698 vw, 724 vw, 778 m (ν SiOSi), 1045 vs ($ν_{as}$ SiOAl), 1136 vs ($ν_{as}$ SiOSi), 1402 vw ($δ_{as}$ CH_3), 1455 vw (ν py C–C/C–N), 1495 vw (ν py C–C/C–N), 1622 vw (ν py C–C/C–N), 2919 vw ($ν_s$ CH_3), 2990 vw ($ν_{as}$ CH_3), 3435 vw (ν O–H).

^{1}H MAS NMR *δ*: 8.7 (py *o*-Ar), 7.4 (py *m*-Ar), 0.1 (–OSn(CH_3)$_3$) ppm.

^{13}C TOSS NMR *δ*: 152.1 (py *o*-Ar), 129.3 (py *m*-Ar), –0.8 (–OSn(CH_3)$_3$) ppm.

^{29}Si MAS NMR *δ*: –101.3 ((CH_3)$_3$SnO*Si*(OSi)$_3$), –107.6 (AlO*Si*(OSi)$_3$) ppm.

Composition: ICP-OES: 1.90 wt% Al, 42.1 wt% Sn; **Gravimetry:** 1.83 wt% Al, 40.5 wt% Sn.

N_2 ads.: non-porous, V_{tot} 0.0144 cm^3 g^{-1}, Type III isotherm.

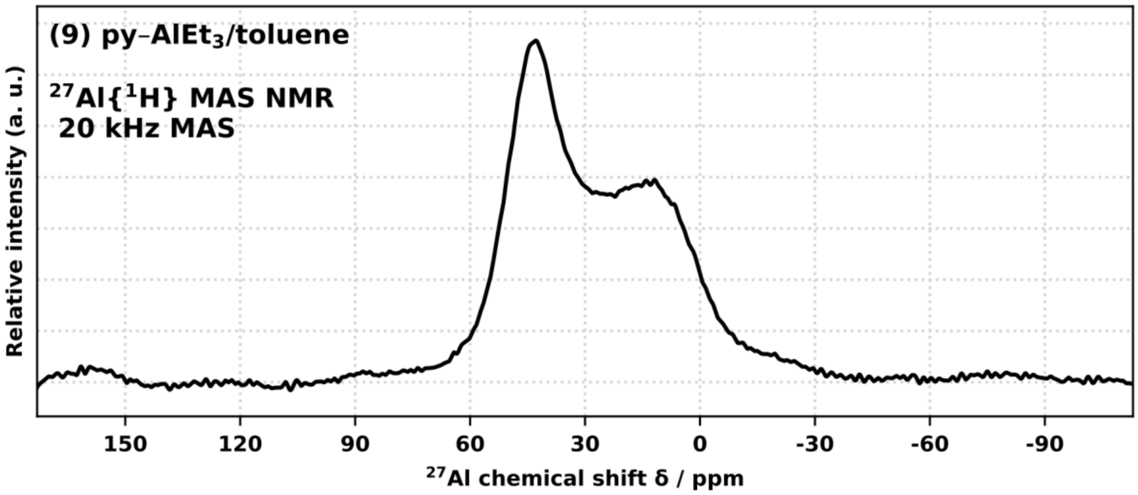

Figure S17. ^{27}Al{^{1}H} MAS NMR spectrum of product **9** (20 kHz MAS).

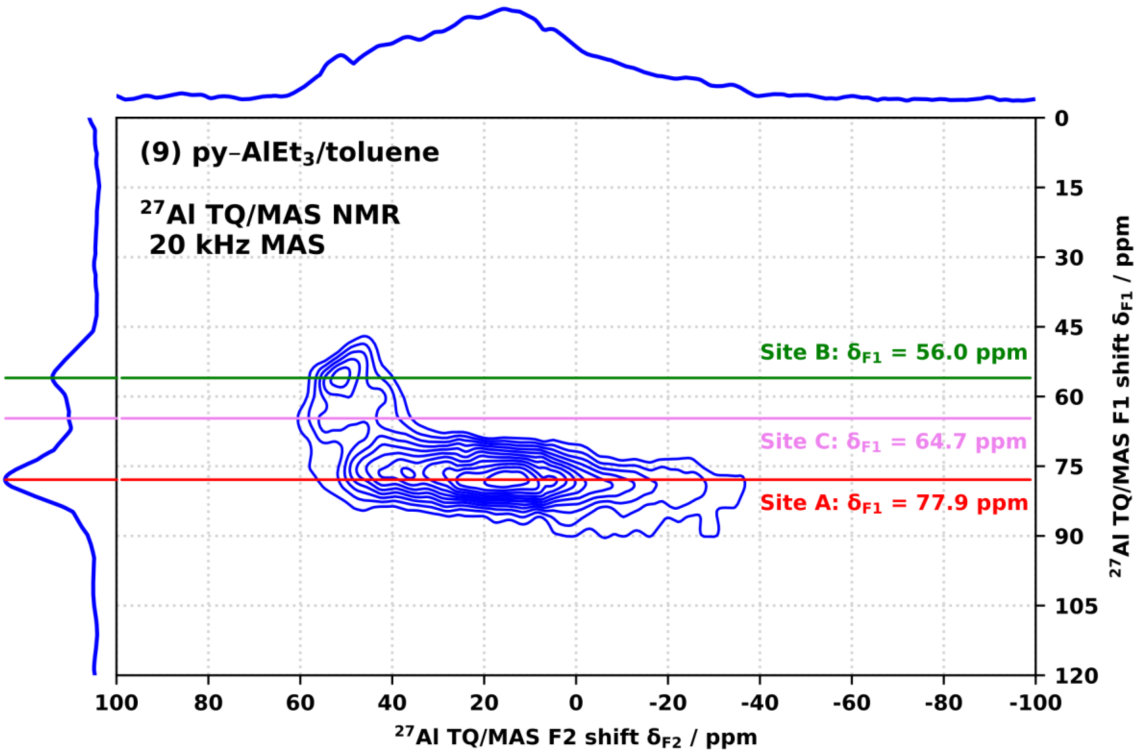

Figure S18. ^{27}Al TQ/MAS NMR spectrum of product **9** (20 kHz MAS).

Table S10. Slices through the ^{27}Al TQ/MAS NMR spectrum of product **9** and the corresponding line shape simulation fits.

Site	^{27}Al line shape simulation fit (quadrupole central transition)			
	δ_{iso} (ppm)	C_Q (MHz)	η	*LB* (fixed)
	55.591	13.928	0.624	1000
A	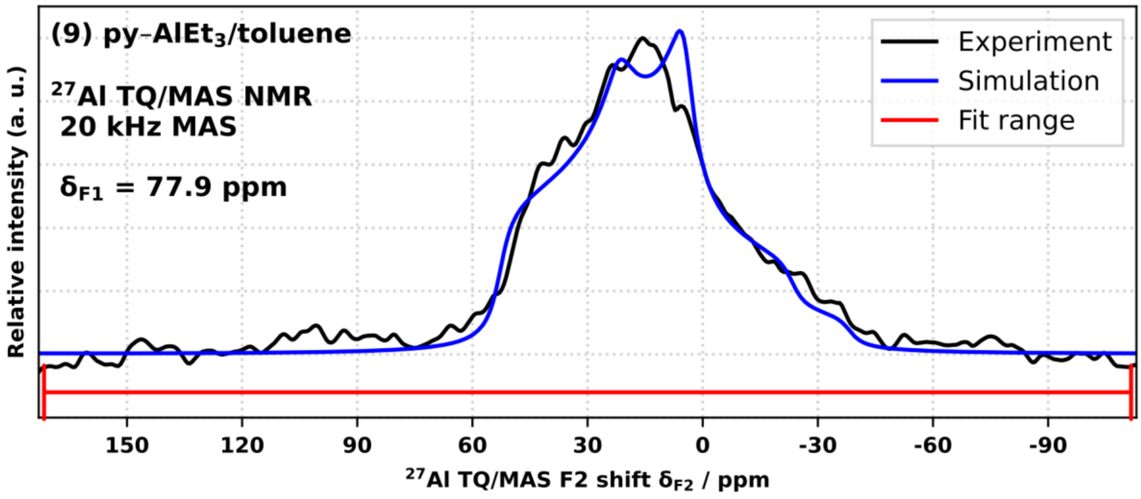			

57.030	6.079	0.482	500

B

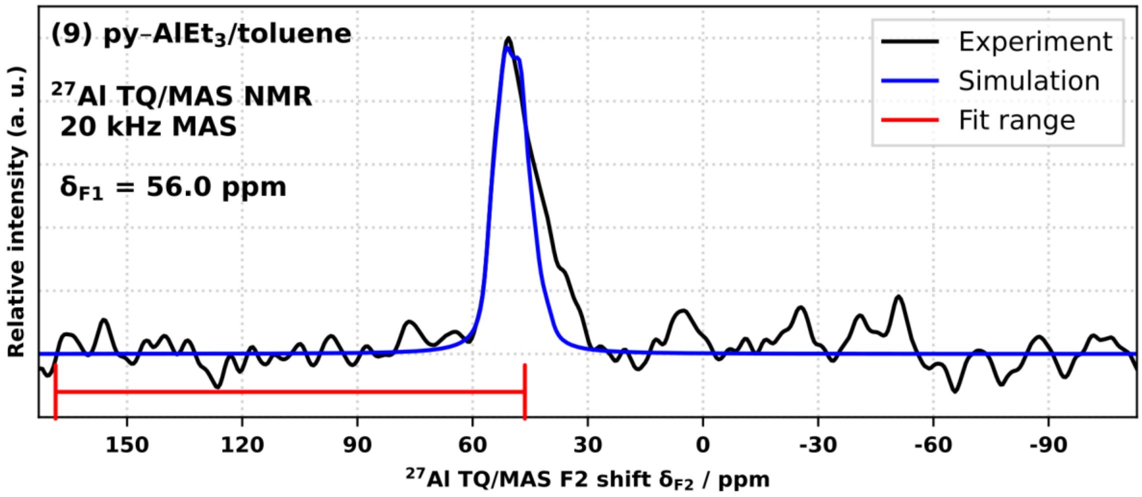

57.767	9.427	0.502	1200

C

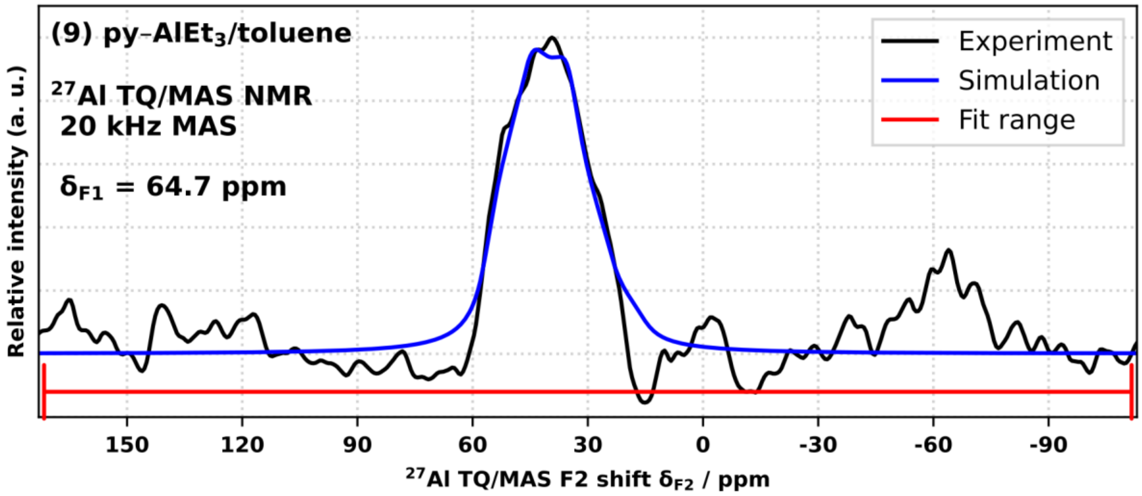

Product 10 (0.5 $Et_3N–AlMe_3$/toluene)

Synthesis: $(Me_3Sn)_8Si_8O_{20}$ (2.1418 g, 1.1545 mmol), $Et_3N–AlMe_3$ (0.1008 g, 0.5818 mmol), toluene (solvent, 30 cm^3). **Byproducts:** 0.3294 g ($SnMe_4$ only).

IR (KBr, cm^{-1}) *v*: 513 w, 541 s, 580 w, 625 s, 663 vw, 723 w, 778 s (ν SiOSi), 900 vw, 1028 vs (ν_{as} SiOAl), 1138 vs (ν_{as} SiOSi), 1402 vw (δ_{as} CH_3), 1470 vw (δ_{as} CH_3), 2919 vw (ν_s CH_3), 2989 vw (ν_{as} CH_3), 3434 vw (ν O–H).

^{1}H MAS NMR *δ*: 2.8 (CH_3CH_2N), 1.1 (CH_3CH_2N), 0.3 (–$OSn(CH_3)_3$) ppm.

^{13}C TOSS NMR *δ*: 50.4 (CH_3CH_2N), 13.2 (CH_3CH_2N), 0.5 (–$OSn(CH_3)_3$) ppm.

^{29}Si MAS NMR *δ*: –101.4 ($(CH_3)_3SnOSi(OSi)_3$), –108.7 ($AlOSi(OSi)_3$) ppm.

Composition: ICP-OES: 0.823 wt% Al, 46.9 wt% Sn; **Gravimetry:** 0.820 wt% Al, 45.9 wt% Sn.

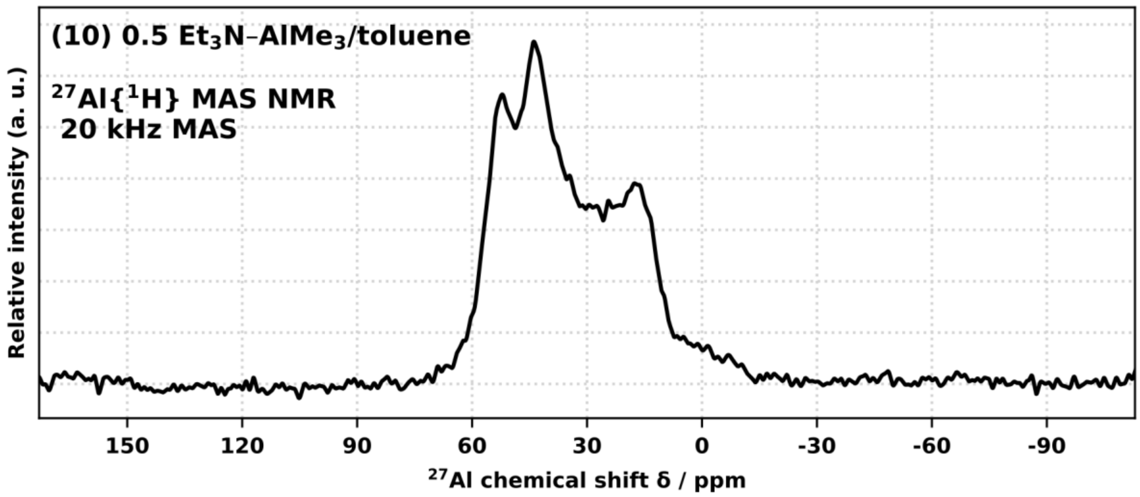

Figure S19. ^{27}Al{^{1}H} MAS NMR spectrum of product **10** (20 kHz MAS).

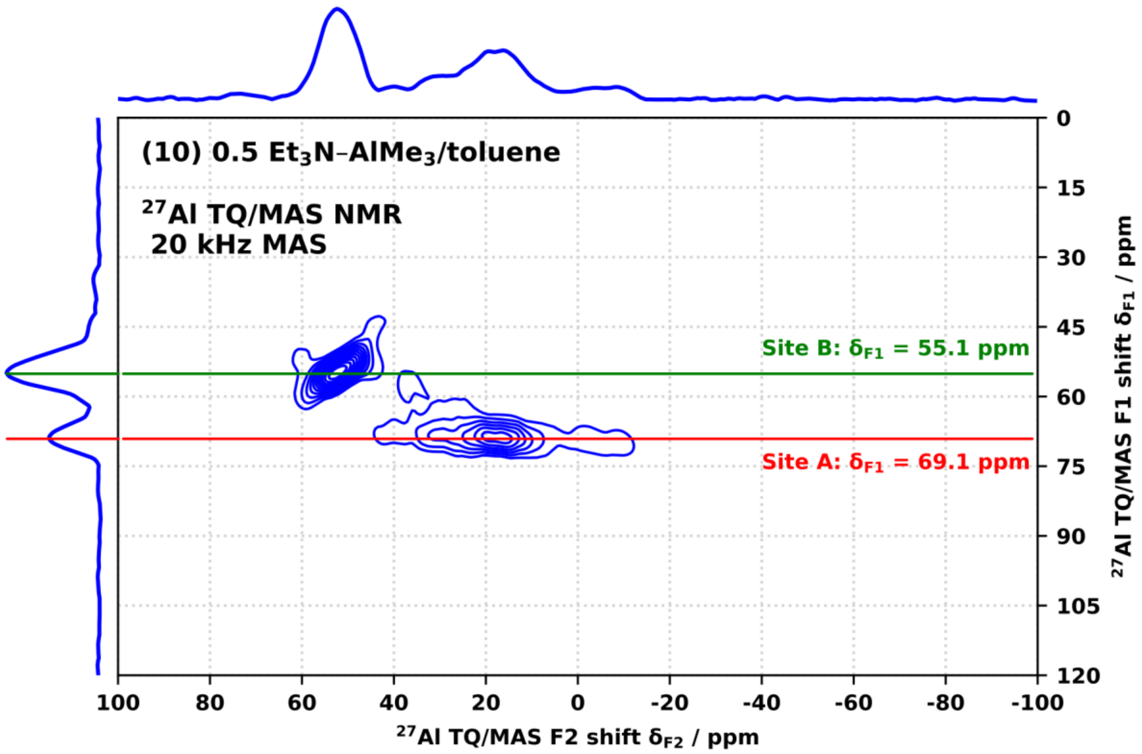

Figure S20. ^{27}Al TQ/MAS NMR spectrum of product **10** (20 kHz MAS).

Table S11. Slices through the ^{27}Al TQ/MAS NMR spectrum of product **10** and the corresponding line shape simulation fits.

Site	^{27}Al line shape simulation fit (quadrupole central transition)			
	**δ_{iso} (ppm)**	**C_Q (MHz)**	***η***	***LB* (fixed)**
	44.918	10.736	0.861	500
**A**	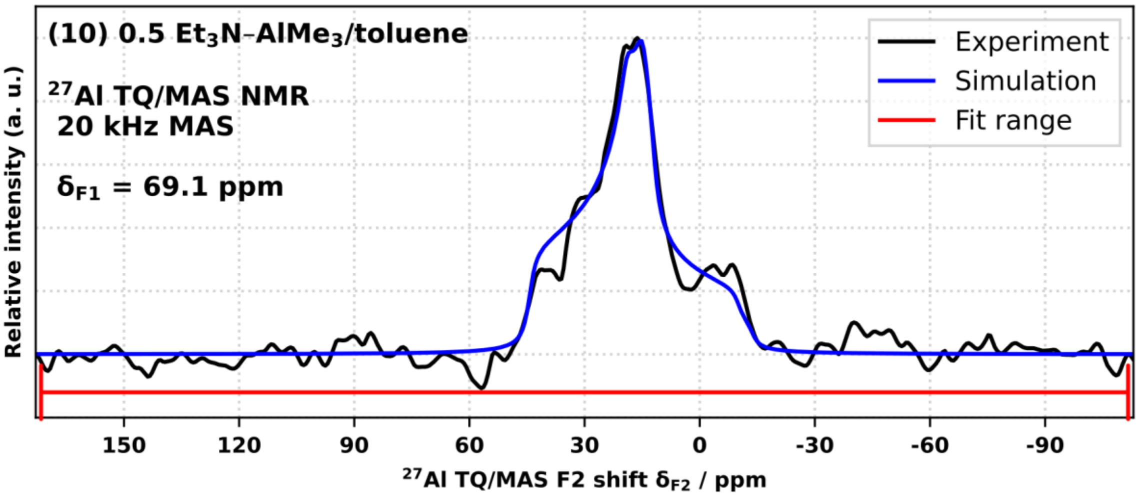			

57.615	5.136	0.500	500

B

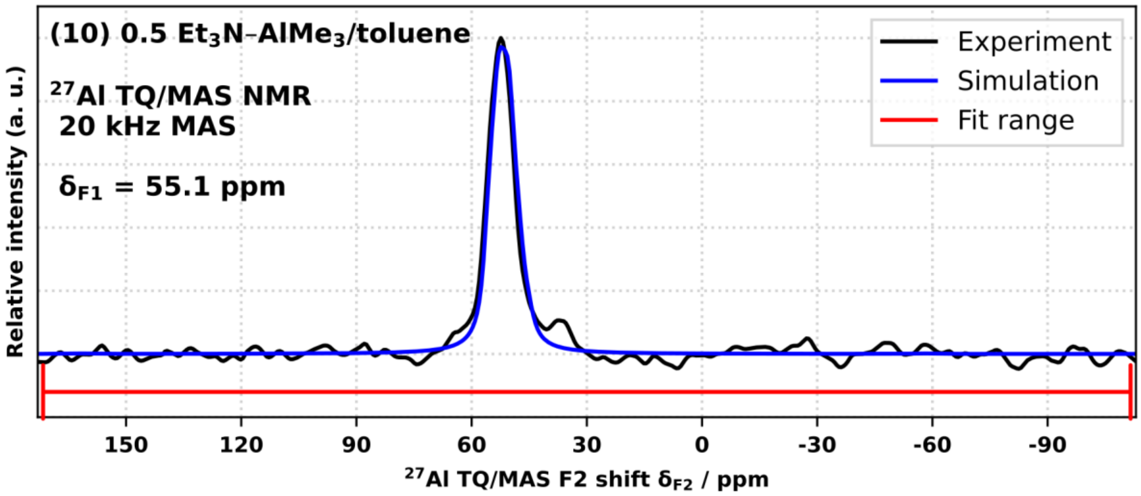

Product 11 (Et_3N–$AlMe_3$/toluene)

Synthesis: $(Me_3Sn)_8Si_8O_{20}$ (2.0705 g, 1.1161 mmol), Et_3N–$AlMe_3$ (0.1934 g, 1.116 mmol), toluene (solvent, 30 cm^3). **Byproducts:** 0.6136 g ($SnMe_4$ only).

IR (KBr, cm^{-1}) *ν*: 513 w, 541 s, 578 w, 623 w, 663 vw, 723 w, 777 m (ν SiOSi), 900 vw, 1034 vs ($ν_{as}$ SiOAl), 1126 vs ($ν_{as}$ SiOSi), 1402 vw ($δ_{as}$ CH_3), 1470 vw ($δ_{as}$ CH_3), 1635 vw (δ HOH), 2919 vw ($ν_s$ CH_3), 2989 vw ($ν_{as}$ CH_3), 3434 vw (ν O–H).

^1H MAS NMR *δ*: 2.8 ($CH_3C\mathit{H}_2N$), 1.1 ($C\mathit{H}_3CH_2N$), 0.2 (–$OSn(C\mathit{H}_3)_3$) ppm.

^{13}C TOSS NMR *δ*: 49.9 ($CH_3\mathit{C}H_2N$), 12.5 ($\mathit{C}H_3CH_2N$), 0.0 (–$OSn(\mathit{C}H_3)_3$) ppm.

^{29}Si MAS NMR *δ*: –101.3 ($(CH_3)_3SnO\mathit{Si}(OSi)_3$), –109.1 ($AlO\mathit{Si}(OSi)_3$) ppm.

Composition: ICP-OES: 1.81 wt% Al, 40.6 wt% Sn; **Gravimetry:** 1.83 wt% Al, 39.6 wt% Sn.

N_2 ads.: non-porous, V_{tot} 0.00920 cm^3 g^{-1}, Type III isotherm.

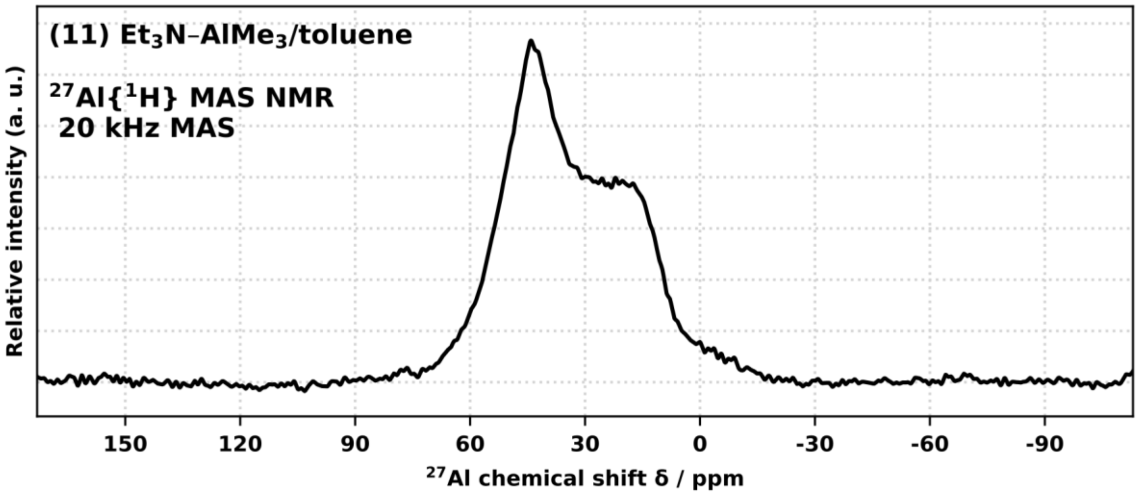

Figure S21. $^{27}Al\{^1H\}$ MAS NMR spectrum of product **11** (20 kHz MAS).

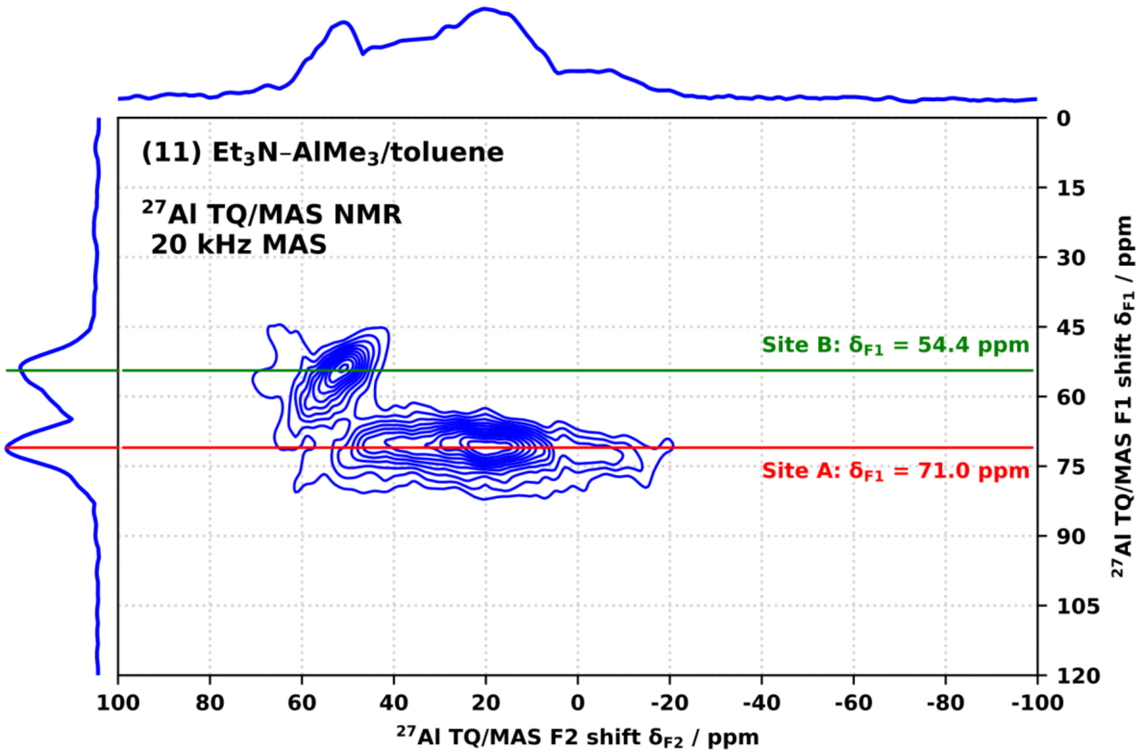

Figure S22. ^{27}Al TQ/MAS NMR spectrum of product **11** (20 kHz MAS).

Table S12. Slices through the ^{27}Al TQ/MAS NMR spectrum of product **11** and the corresponding line shape simulation fits.

Site	^{27}Al line shape simulation fit (quadrupole central transition)			
	δ_{iso} (ppm)	C_Q (MHz)	η	*LB* (fixed)
	51.429	12.051	0.835	1800

A

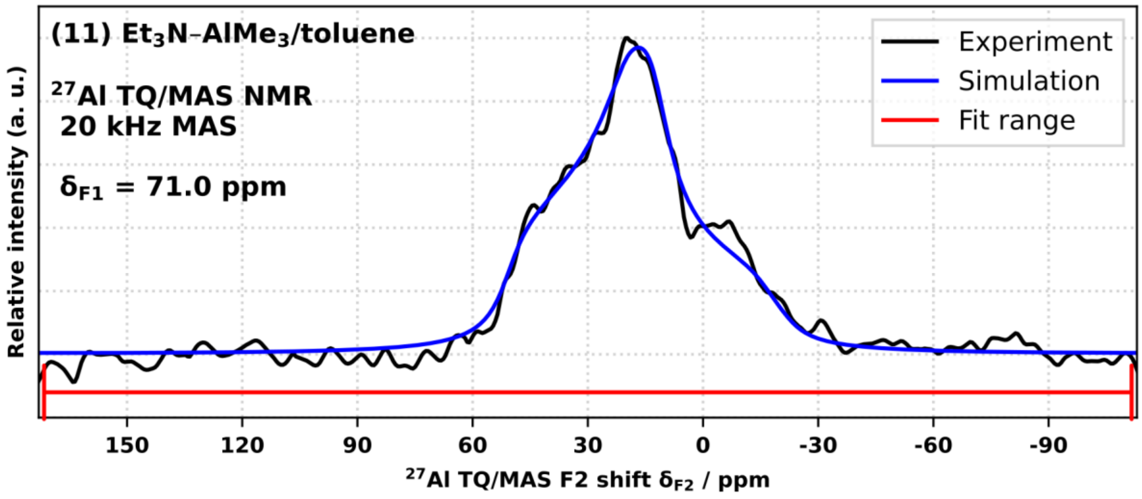

57.088	6.030	0.540	500

B

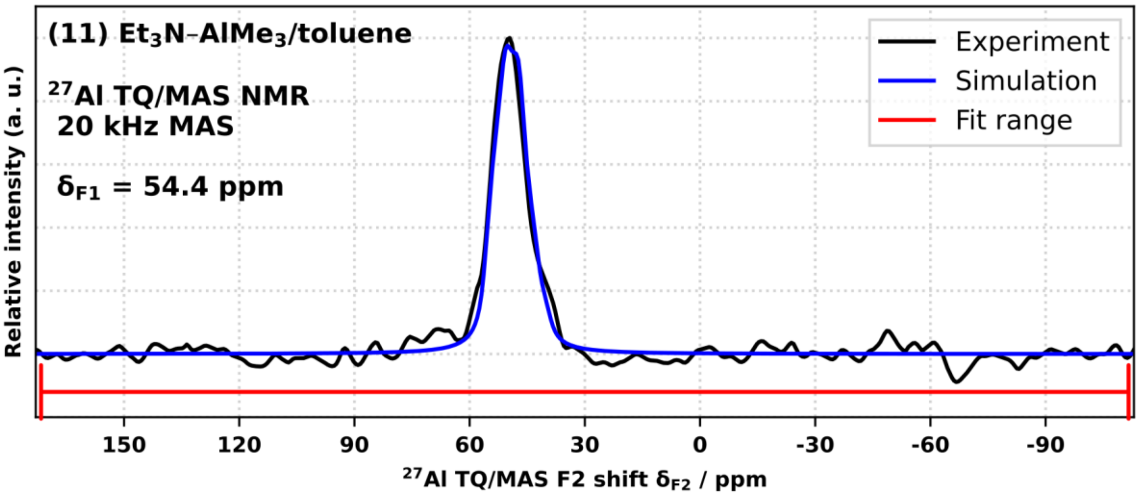

Product 12 (0.5 TEPO–$AlCl_3$/THF)

Synthesis: $(Me_3Sn)_8Si_8O_{20}$ (2.2878 g, 1.2332 mmol), $AlCl_3$ (0.0814 g, 0.610 mmol),TEPO (0.0892 g, 0.665 mmol), THF (solvent, 32 cm^3). **Byproducts:** 0.3663 g, *n(SnMe*$_4$*)/n(SnMe*$_3$*Cl)* = 0.0.

IR (KBr, cm^{-1}) *v*: 513 w, 542 s, 583 w, 623 s, 668 w, 727 w, 779 s (ν SiOSi), 1026 vs (ν_{as} SiOAl), 1136 vs (ν_{as} SiOSi), 1408 vw (δ_{as} CH_3), 1460 vw (δ_{as} CH_3), 2890 vw (ν C–H), 2918 vw (ν_s CH_3), 2942 vw (ν C–H), 2988 vw (ν_{as} CH_3), 3440 vw (ν O–H).

^{1}H MAS NMR *δ*: 1.9 (CH_3CH_2P), 1.1 (CH_3CH_2P), 0.2 (–OSn$(CH_3)_3$) ppm.

^{13}C TOSS NMR *δ*: 21.1 (CH_3CH_2P), 9.6 (CH_3CH_2P), 1.1 (–OSn$(CH_3)_3$) ppm.

^{29}Si MAS NMR *δ*: –101.4 ($(CH_3)_3$SnO*Si*$(OSi)_3$), –107.7 (AlO*Si*$(OSi)_3$) ppm.

^{31}P MAS NMR *δ*: 75.8 (TEPO–AlO_3), 55.7 (trace, TEPO) ppm.

Composition: ICP-OES: 0.809 wt% Al, 45.7 wt% Sn, 1.02 wt% P; **Gravimetry:** 0.790 wt% Al, 45.7 wt% Sn, 0.907 wt% P.

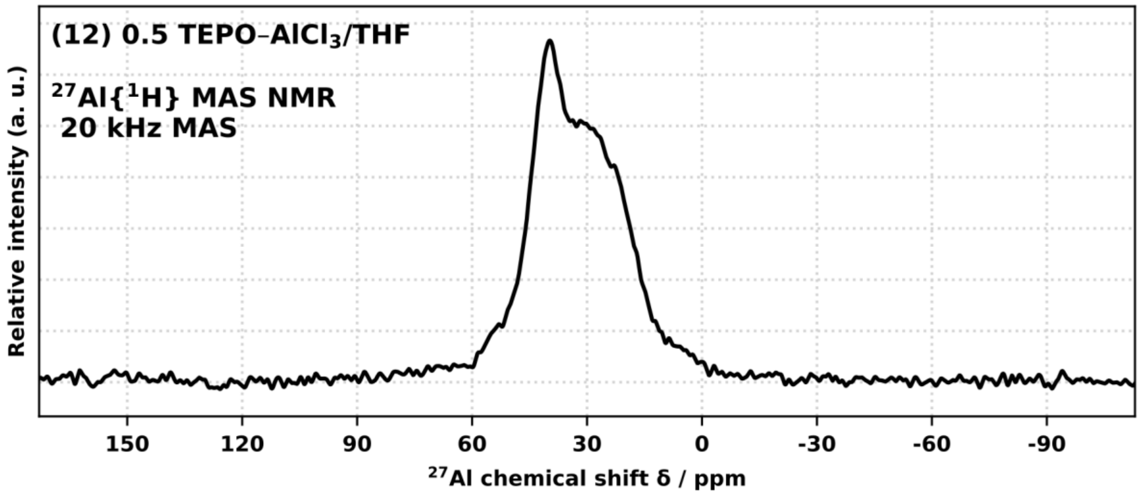

Figure S23. ^{27}Al{^{1}H} MAS NMR spectrum of product **12** (20 kHz MAS).

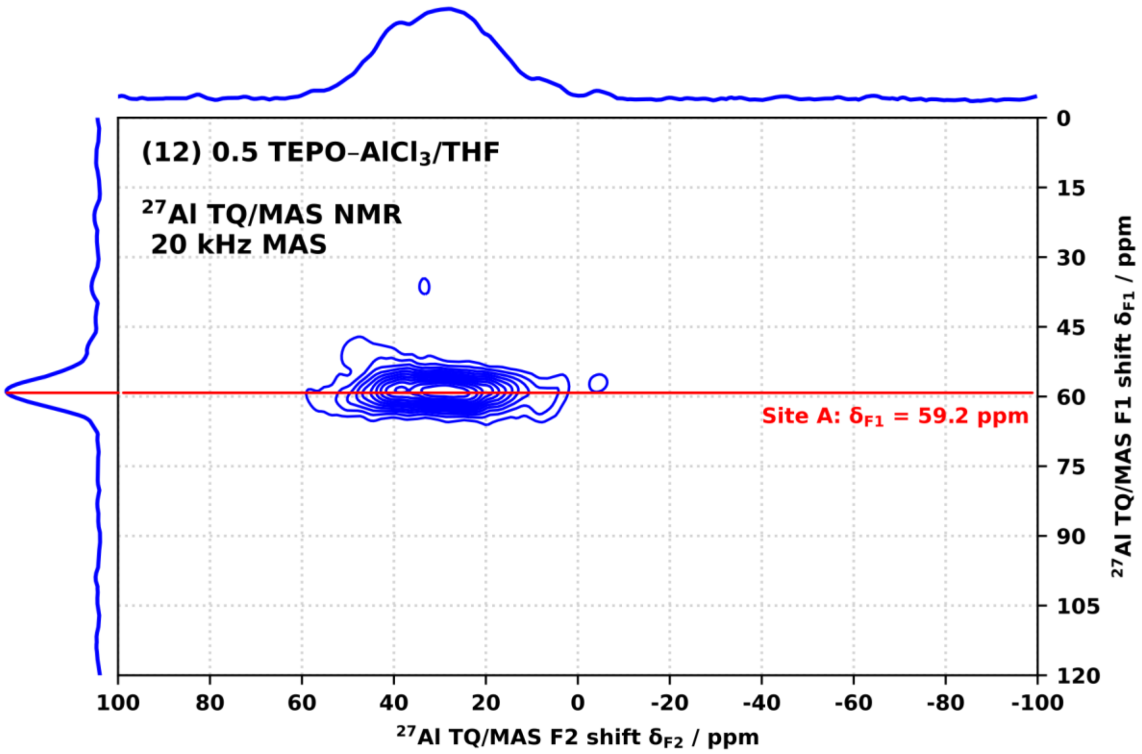

Figure S24. ^{27}Al TQ/MAS NMR spectrum of product **12** (20 kHz MAS).

Table S13. Slices through the ^{27}Al TQ/MAS NMR spectrum of product **12** and the corresponding line shape simulation fits.

Site	^{27}Al line shape simulation fit (quadrupole central transition)			
	δ_{iso} (ppm)	C_Q (MHz)	η	*LB* (fixed)
	49.897	10.120	0.481	1300
A	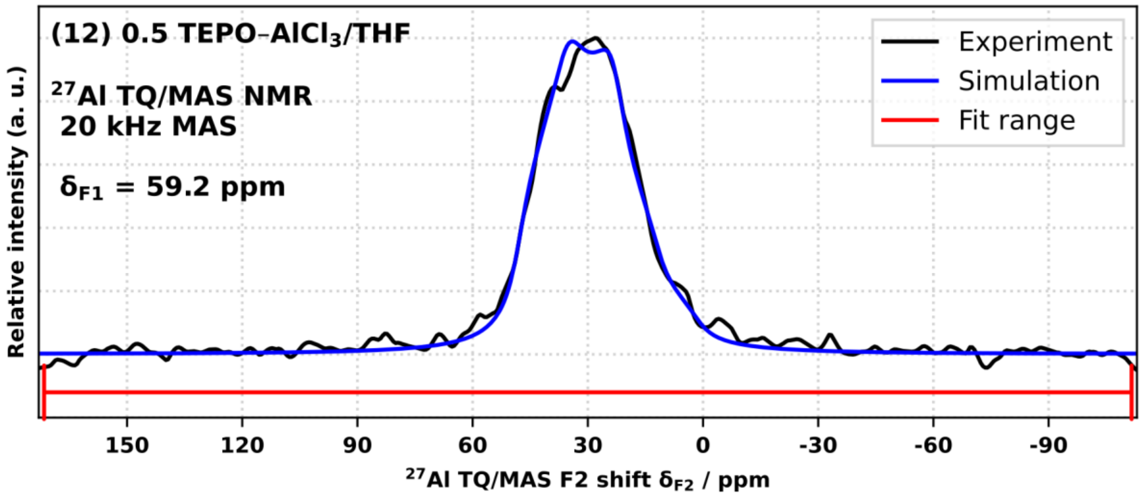			

Product 13 (TEPO–$AlCl_3$/THF)

Synthesis: $(Me_3Sn)_8Si_8O_{20}$ (1.9653 g, 1.0594 mmol), $AlCl_3$ (0.1400 g, 1.050 mmol), TEPO (0.1428 g, 1.064 mmol), THF (solvent, 30 cm^3). **Byproducts:** 0.6063 g, $n(SnMe_4)/n(SnMe_3Cl)$ = 0.0060.

IR (KBr, cm^{-1}) *ν*: 513 w, 542 m, 583 w, 623 m, 668 w, 727 w, 779 m (ν SiOSi), 1040 vs (ν_{as} SiOAl), 1124 vs (ν_{as} SiOSi), 1408 vw (δ_{as} CH_3), 1460 vw (δ_{as} CH_3), 2890 vw (ν C–H), 2918 vw (ν_s CH_3), 2942 vw (ν C–H), 2985 vw (ν_{as} CH_3), 3440 vw (ν O–H).

^{13}C TOSS NMR *δ*: 20.5 (CH_3*C*H_2P), 8.9 (*C*H_3CH_2P), 0.1 (–OSn(*C*$H_3)_3$) ppm.

^{29}Si MAS NMR *δ*: –101.1 ($(CH_3)_3$SnO*Si*$(OSi)_3$), –108.5 (AlO*Si*$(OSi)_3$) ppm.

^{31}P MAS NMR *δ*: 81.0 (shoulder, TEPO–$AlClO_2$), 75.3 (TEPO–AlO_3), 54.1 (trace, TEPO) ppm.

Composition: ICP-OES: 1.79 wt% Al, 43.1 wt% Sn, 2.12 wt% P; **Gravimetry:** 1.73 wt% Al, 39.3 wt% Sn, 1.98 wt% P.

N_2 ads.: non-porous, V_{tot} 0.00997 cm^3 g^{-1}, Type III isotherm.

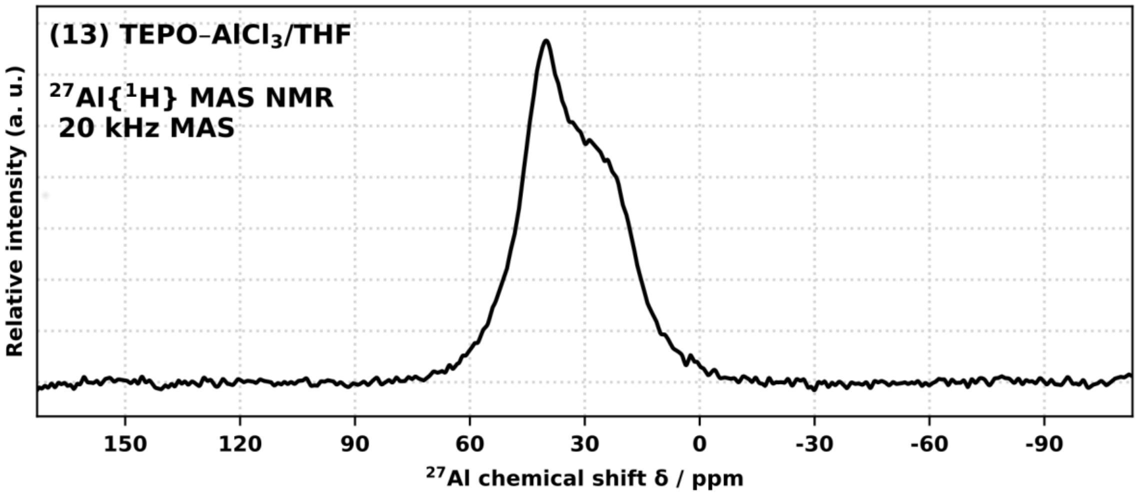

Figure S25. ^{27}Al{^{1}H} MAS NMR spectrum of product **13** (20 kHz MAS).

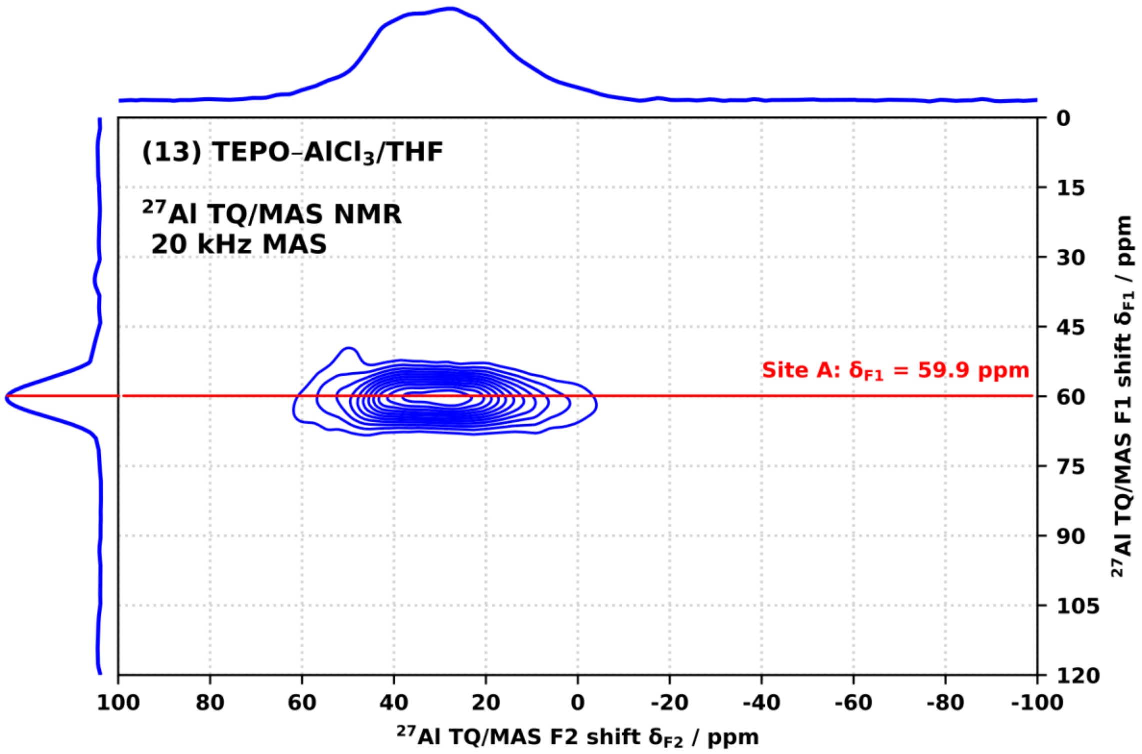

Figure S26. ^{27}Al TQ/MAS NMR spectrum of product **13** (20 kHz MAS).

Table S14. Slices through the ^{27}Al TQ/MAS NMR spectrum of product **13** and the corresponding line shape simulation fits.

Site	^{27}Al line shape simulation fit (quadrupole central transition)			
	δ_{iso} (ppm)	C_Q (MHz)	η	*LB* (fixed)
	50.976	10.525	0.405	1800

A

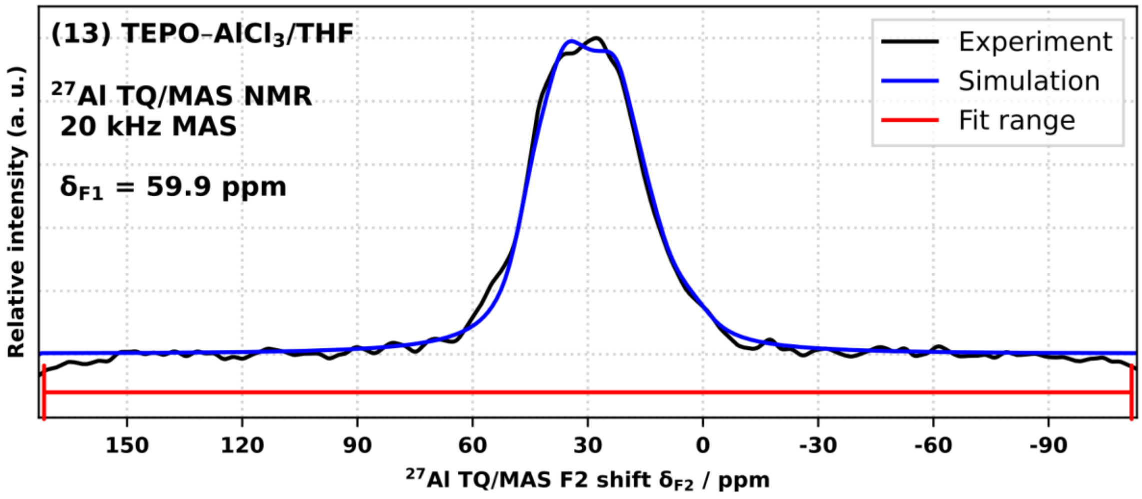

Product 14 (0.5 TEPO–$AlMe_3$/toluene)

Synthesis: $(Me_3Sn)_8Si_8O_{20}$ (2.0100 g, 1.0835 mmol), TEPO–$AlMe_3$ (0.1117 g, 0.5416 mmol), toluene (solvent, 30 cm^3). **Byproducts:** 0.2911 g ($SnMe_4$ only).

IR (KBr, cm^{-1}) *v*: 513 w, 542 s, 583 w, 623 s, 668 w, 727 w, 779 s (v SiOSi), 1026 vs (v_{as} SiOAl), 1138 vs (v_{as} SiOSi), 1407 vw (δ_{as} CH_3), 1460 vw (δ_{as} CH_3), 2890 vw (v C–H), 2920 vw (v_s CH_3), 2984 vw (v_{as} CH_3), 3440 vw (v O–H).

^{1}H MAS NMR *δ*: 1.8 ($CH_3C\mathit{H}_2P$), 1.0 ($C\mathit{H}_3CH_2P$), 0.1 (–OSn($C\mathit{H}_3)_3$) ppm.

^{13}C TOSS NMR *δ*: 21.0 ($CH_3\mathit{C}H_2P$), 10.0 ($\mathit{C}H_3CH_2P$), 1.1 (–OSn($\mathit{C}H_3)_3$) ppm.

^{29}Si MAS NMR *δ*: –101.4 ($(CH_3)_3SnO\mathit{Si}(OSi)_3$), –108.1 ($AlO\mathit{Si}(OSi)_3$) ppm.

^{31}P MAS NMR *δ*: 81.0 (trace shoulder, TEPO–$AlMeO_2$), 75.4 (TEPO–AlO_3), 54.9 (trace, TEPO) ppm.

Composition: ICP-OES: 0.866 wt% Al, 48.8 wt% Sn, 1.02 wt% P; **Gravimetry:** 0.798 wt% Al, 45.7 wt% Sn, 0.916 wt% P.

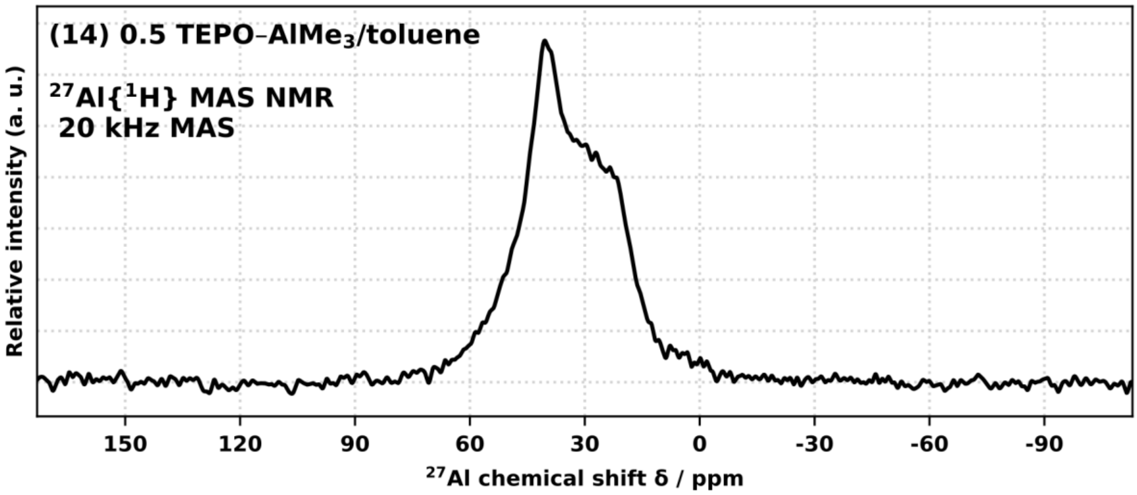

Figure S27. ^{27}Al{^{1}H} MAS NMR spectrum of product **14** (20 kHz MAS).

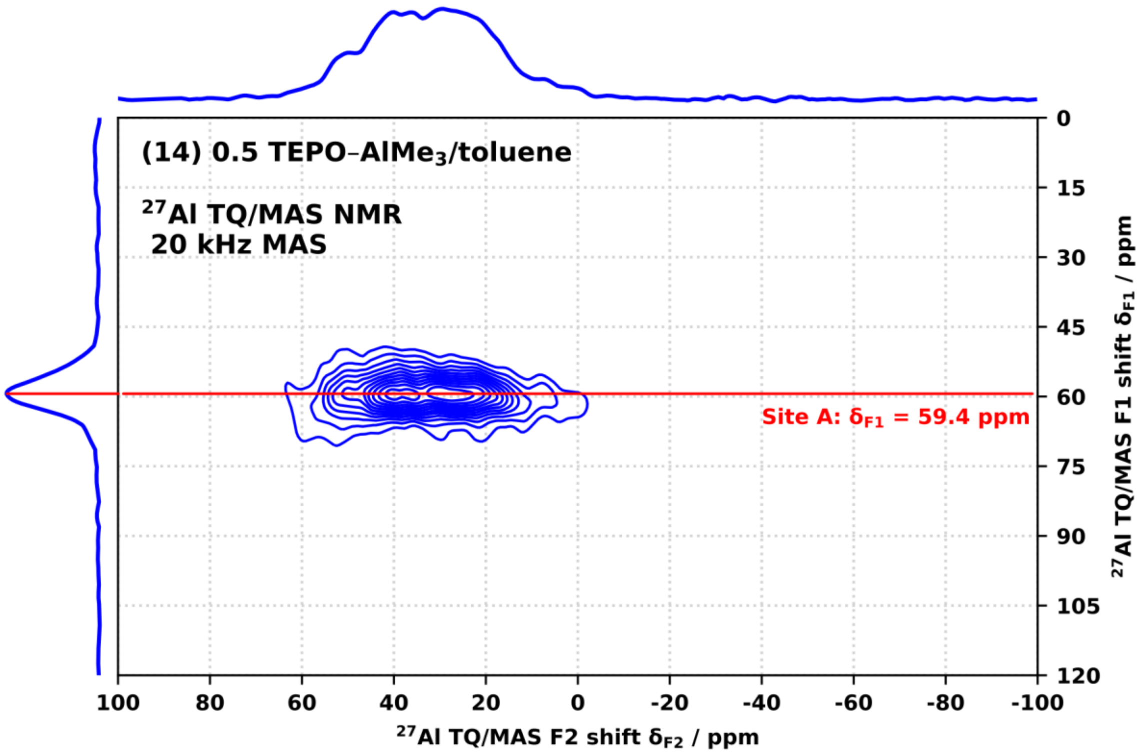

Figure S28. ^{27}Al TQ/MAS NMR spectrum of product **14** (20 kHz MAS).

Table S15. Slices through the ^{27}Al TQ/MAS NMR spectrum of product **14** and the corresponding line shape simulation fits.

Site	^{27}Al line shape simulation fit (quadrupole central transition)			
	δ_{iso} (ppm)	C_Q (MHz)	η	*LB* (fixed)
	54.080	10.805	0.375	1800
A	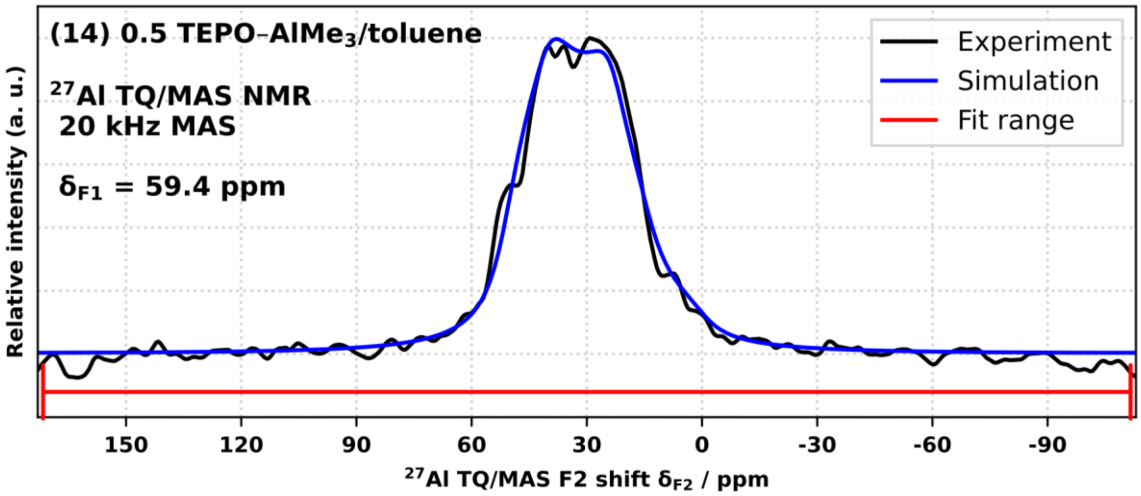			

Product 15 (TEPO–$AlMe_3$/toluene)

Synthesis: $(Me_3Sn)_8Si_8O_{20}$ (2.0766 g, 1.1194 mmol), TEPO–$AlMe_3$ (0.2267 g, 1.099 mmol), toluene (solvent, 30 cm^3). **Byproducts:** 0.4946 g ($SnMe_4$ only).

IR (KBr, cm^{-1}) *ν*: 513 w, 542 s, 583 w, 623 m, 668 w, 727 w, 779 s (ν SiOSi), 1037 vs ($ν_{as}$ SiOAl), 1134 vs ($ν_{as}$ SiOSi), 1410 vw ($δ_{as}$ CH_3), 1462 vw ($δ_{as}$ CH_3), 1637 vw (δ HOH), 2853 vw ($ν_s$ CH_3), 2890 vw (ν C–H), 2920 vw ($ν_s$ CH_3), 2984 vw ($ν_{as}$ CH_3), 3440 vw (ν O–H).

^{1}H MAS NMR *δ*: 1.8 ($CH_3C\mathit{H}_2P$), 1.0 ($C\mathit{H}_3CH_2P$), 0.2 (–OSn($C\mathit{H}_3$)$_3$) , –1.3 (AlCH_3) ppm.

^{13}C TOSS NMR *δ*: 21.3 ($CH_3\mathit{C}H_2P$), 9.7 ($\mathit{C}H_3CH_2P$), 1.0 (–OSn($\mathit{C}H_3$)$_3$), –10.2 (Al$\mathit{C}H_3$) ppm.

^{29}Si MAS NMR *δ*: –100.9 ((CH_3)$_3$SnO*Si*(OSi)$_3$), –107.5 (AlO*Si*(OSi)$_3$) ppm.

^{31}P MAS NMR *δ*: 80.9 (shoulder, TEPO–$AlMeO_2$), 74.6 (TEPO–AlO_3), 53.8 (trace, TEPO) ppm.

Composition: ICP-OES: 1.83 wt% Al, 43.5 wt% Sn, 2.05 wt% P; **Gravimetry:** 1.64 wt% Al, 40.6 wt% Sn, 1.88 wt% P.

N_2 ads.: non-porous, V_{tot} 0.00213 cm^3 g^{-1}, Type III isotherm.

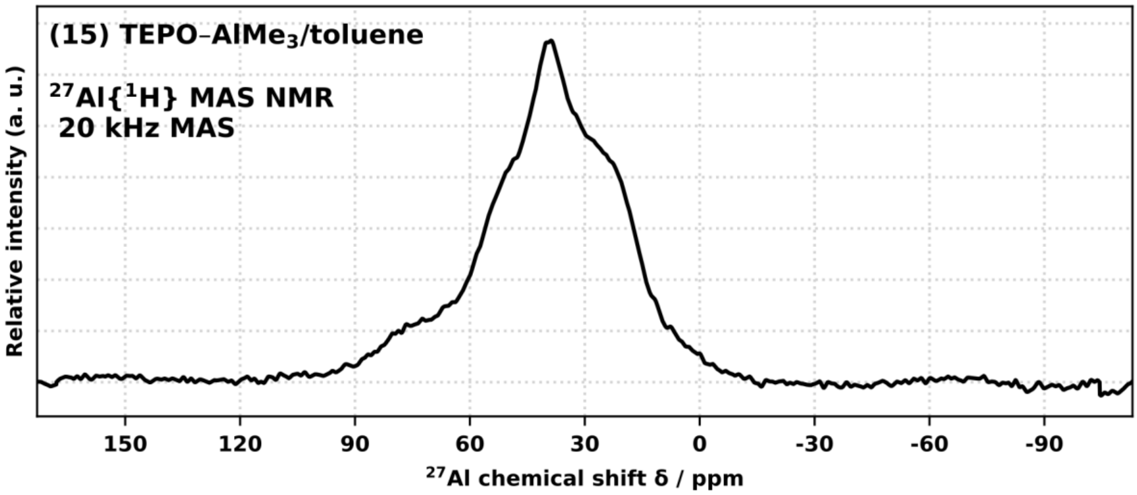

Figure S29. ^{27}Al{^{1}H} MAS NMR spectrum of product **15** (20 kHz MAS).

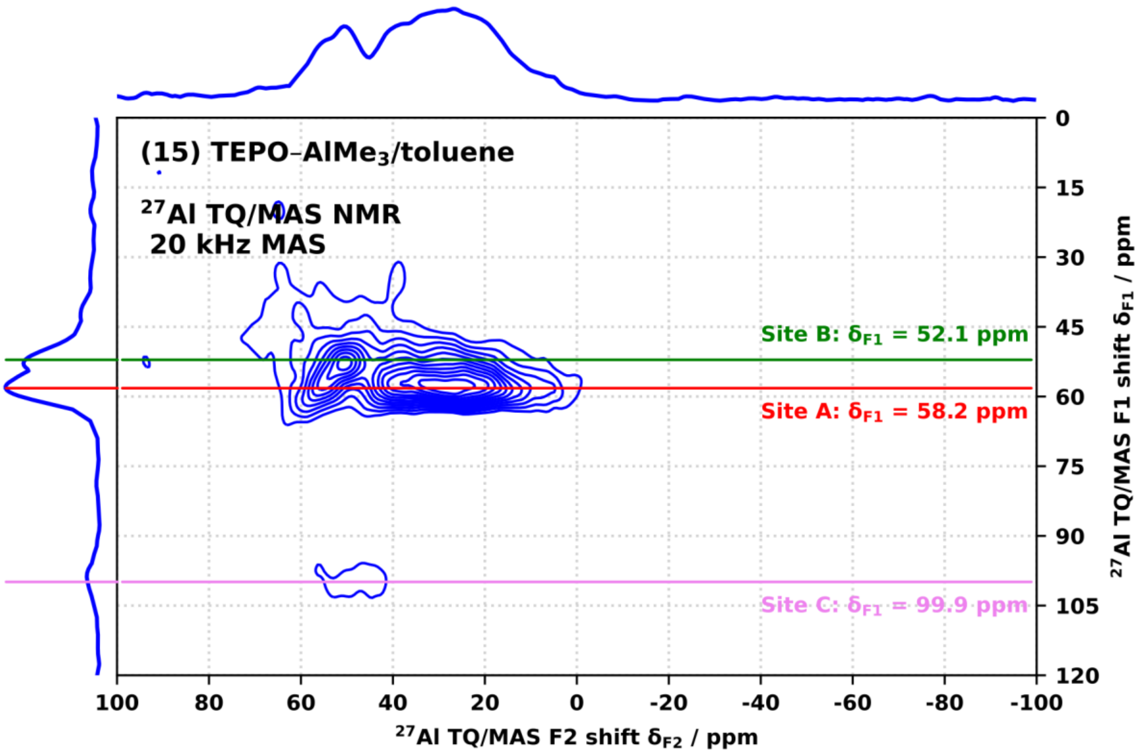

Figure S30. ^{27}Al TQ/MAS NMR spectrum of product **15** (20 kHz MAS).

Table S16. Slices through the ^{27}Al TQ/MAS NMR spectrum of product **15** and the corresponding line shape simulation fits.

Site	^{27}Al line shape simulation fit (quadrupole central transition)			
	δ_{iso} (ppm)	C_Q (MHz)	η	*LB* (fixed)
	48.313	10.259	0.461	1400
A	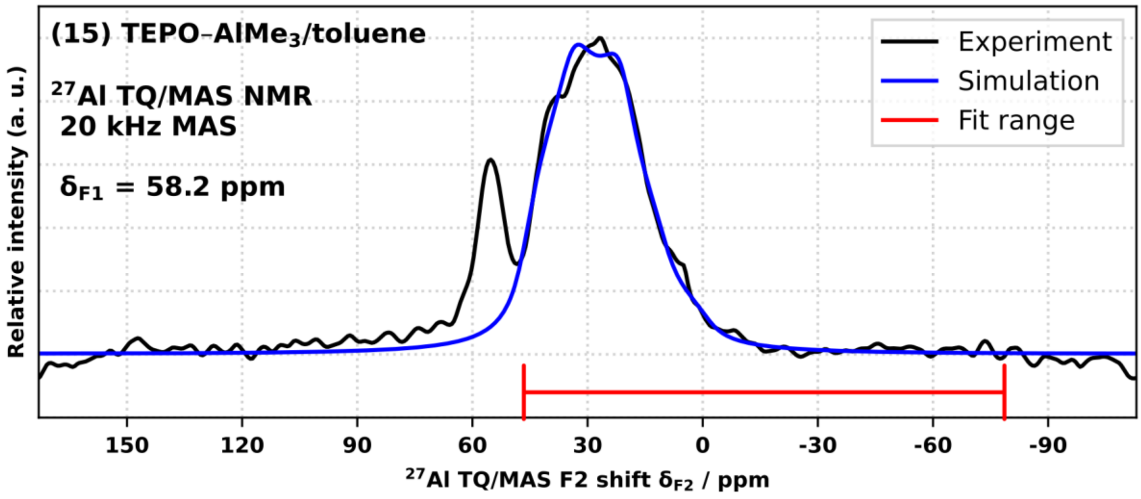			

58.613	6.260	0.600 (fixed)	1200

B

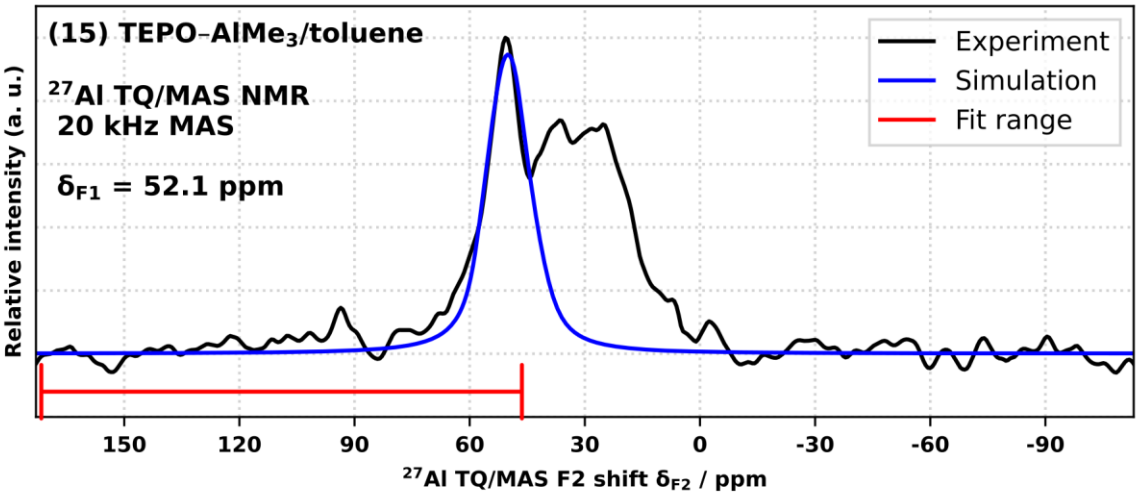

66.908	12.822	0.439	1000

C

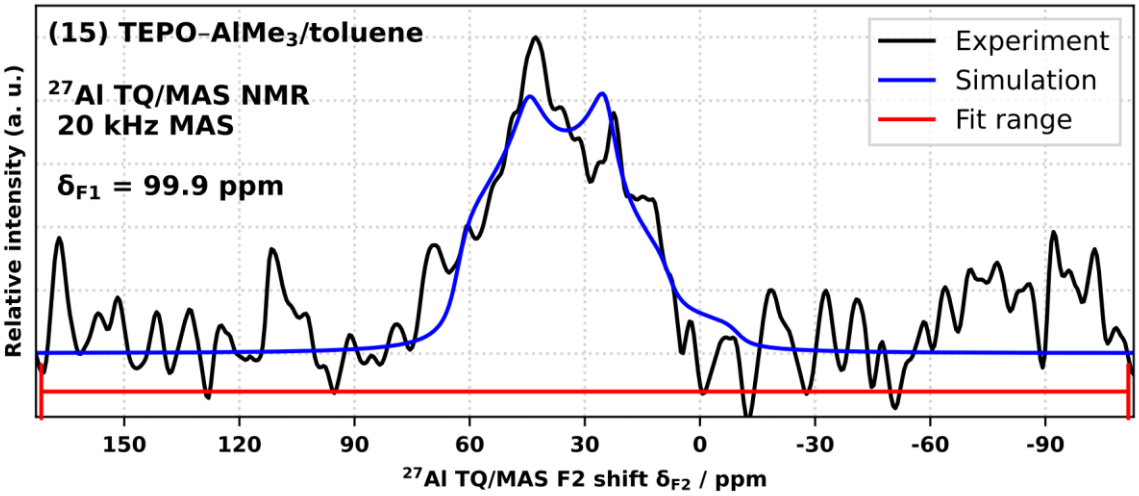

Product 16 (0.5 x 0.75 $[Me_4N][AlCl_4]$/THF)

Synthesis: $(Me_3Sn)_8Si_8O_{20}$ (2.0914 g, 1.1273 mmol), $[Me_4N][AlCl_4]$ (0.1018 g, 0.4190 mmol), THF (solvent, 30 cm^3). **Byproducts:** 0.3286 g, *n*($SnMe_4$)/*n*($SnMe_3Cl$) = 0.1568.

IR (KBr, cm^{-1}) *ν*: 513 m, 542 s, 623 s, 672 w, 721 w, 778 m (ν SiOSi), 1030 vs (ν_{as} SiOAl), 1134 vs (ν_{as} SiOSi), 1402 vw (δ_{as} CH_3), 1488 vw (δ_{as} CH_3), 1637 vw (δ HOH), 2919 vw (ν_s CH_3), 2990 vw (ν_{as} CH_3), 3435 vw (ν O–H).

^{1}H MAS NMR *δ*: 3.0 (CH_3N), 0.1 (–$OSn(CH_3)_3$) ppm.

^{13}C TOSS NMR *δ*: 59.3 (CH_3N), 0.5 (–$OSn(CH_3)_3$) ppm.

^{29}Si MAS NMR *δ*: –101.9 ($(CH_3)_3SnOSi(OSi)_3$), –108.1 ($AlOSi(OSi)_3$) ppm.

Composition: ICP-OES: 0.668 wt% Al, 50.0 wt% Sn; **Gravimetry:** 0.606 wt% Al, 46.8 wt% Sn.

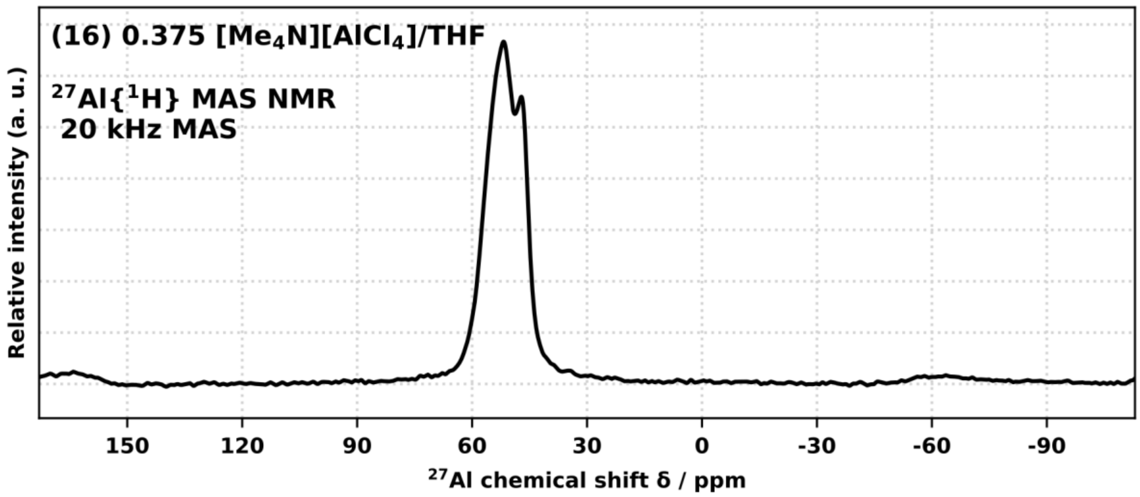

Figure S31. ^{27}Al{^{1}H} MAS NMR spectrum of product **16** (20 kHz MAS).

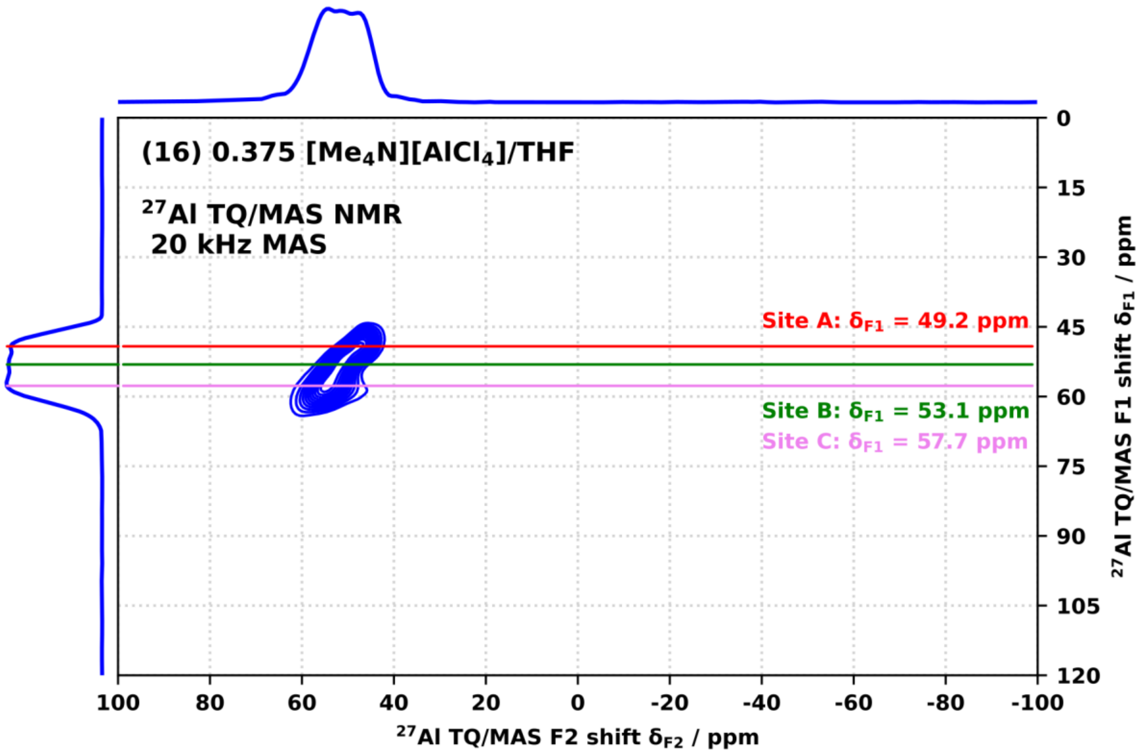

Figure S32. ^{27}Al TQ/MAS NMR spectrum of product **16** (20 kHz MAS).

Table S17. Slices through the ^{27}Al TQ/MAS NMR spectrum of product **16** and the corresponding line shape simulation fits.

Site	^{27}Al line shape simulation fit (quadrupole central transition)			
	δ_{iso} (ppm)	C_Q (MHz)	η	*LB* (fixed)
	51.683	4.444	0.477	500

A

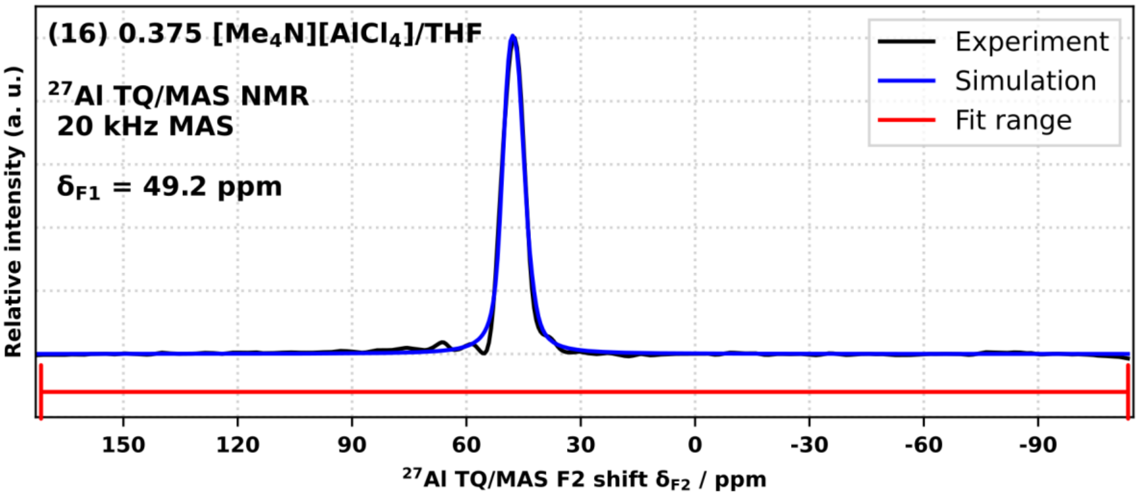

55.349	4.542	0.508	500

B

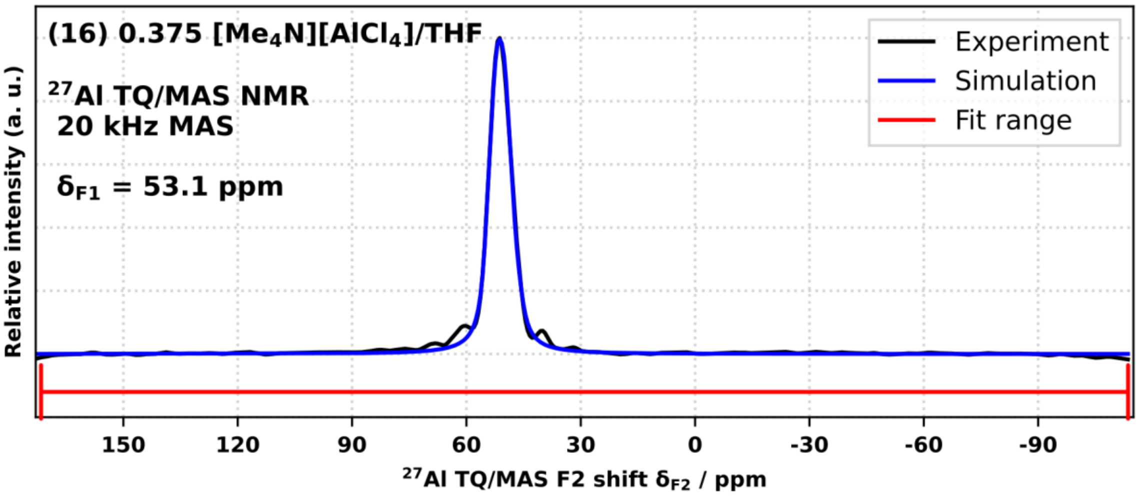

59.231	5.177	0.504	500

C

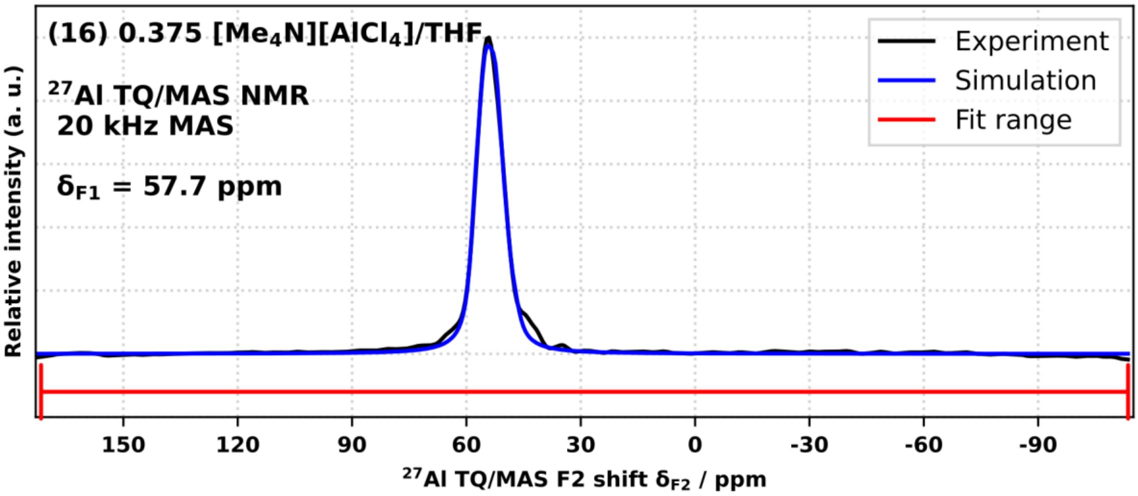

Table S18. Peak data for the deconvolution of ^{29}Si MAS NMR spectra of prepared products (oligomers).

Product	^{29}Si Site	Chemical shift δ_{Si} (ppm)	Relative intensity	Width (FWHM[a]) (Hz)	Lorentzian + gaussian mix ratio (% gauss.)	Relative area
**1**	SnO*Si*	-101.96	0.722	411.7	62	297.09
	AlO*Si*	-107.02	0.278	800.0	35	222.78
**2**	SnO*Si*	-101.88	0.880	374.4	57	329.33
	AlO*Si*	-107.64	0.120	796.2	0	95.85
**3**	SnO*Si*	-103.20	0.778	415.7	46	323.57
	AlO*Si*	-108.78	0.222	726.3	16	160.98
**4**	SnO*Si*	-101.79	0.850	381.6	46	324.22
	AlO*Si*	-107.77	0.150	482.5	0	72.50
**5**	SnO*Si*	-101.01	0.739	428.3	51	316.57
	AlO*Si*	-106.40	0.261	742.9	0	193.78
**6**	SnO*Si*	-101.34	0.741	415.7	47	308.02
	AlO*Si*	-106.49	0.259	995.6	0	257.84
**7**	SnO*Si*	-101.26	0.915	389.0	45	355.80
	AlO*Si*	-107.30	0.085	683.5	0	58.36
**8**	SnO*Si*	-101.09	0.744	423.7	58	315.45
	AlO*Si*	-106.36	0.256	740.6	32	189.23
**9**	SnO*Si*	-101.37	0.772	402.4	39	310.88
	AlO*Si*	-107.10	0.228	702.5	0	159.82
**10**	SnO*Si*	-101.20	0.807	369.4	45	298.09
	AlO*Si*	-108.64	0.193	338.7	0	65.39
**11**	SnO*Si*	-101.37	0.709	423.2	50	300.02
	AlO*Si*	-108.66	0.291	555.0	11	161.59
**12**	SnO*Si*	-101.23	0.869	349.1	45	303.31
	AlO*Si*	-107.81	0.131	619.5	0	81.21
**13**	SnO*Si*	-101.22	0.762	387.5	43	295.33
	AlO*Si*	-107.85	0.238	596.9	20	141.95
**14**	SnO*Si*	-101.13	0.854	353.3	44	301.79
	AlO*Si*	-107.93	0.146	524.0	0	76.42
**15**	SnO*Si*	-100.79	0.797	388.3	40	309.28
	AlO*Si*	-107.33	0.203	574.5	0	116.88
**16**	SnO*Si*	-101.79	0.910	351.0	25	319.28
	AlO*Si*	-108.92	0.090	881.7	0	79.60

[a] FWHM …full width at half maximum

Table S19. Degrees of condensation of $-SnMe_3$ groups calculated from different sources according to procedures described in Section S2 (the input data for Figure S33).

Product	Degree of condensation of $-SnMe_3$ groups		
	gravimetry	ICP-OES	^{29}Si NMR
	$DC_G(-SnMe_3)$ (%)	$DC_E(-SnMe_3)$ (%)	$DC_N(-SnMe_3)$ (%)
**1**	42.38	39.72	42.85
**2**	21.56	16.46	22.54
**3**	38.29	37.17	33.22
**4**	20.38	15.71	18.28
**5**	38.75	35.18	37.97
**6**	44.28	41.01	45.57
**7**	22.00	13.35	14.09
**8**	38.51	34.79	37.50
**9**	37.07	34.47	33.95
**10**	19.94	18.08	17.99
**11**	38.43	36.79	35.01
**12**	18.63	18.59	21.12
**13**	35.92	29.70	32.46
**14**	18.78	13.18	20.21
**15**	30.88	25.93	27.43
**16**	18.48	12.86	19.96

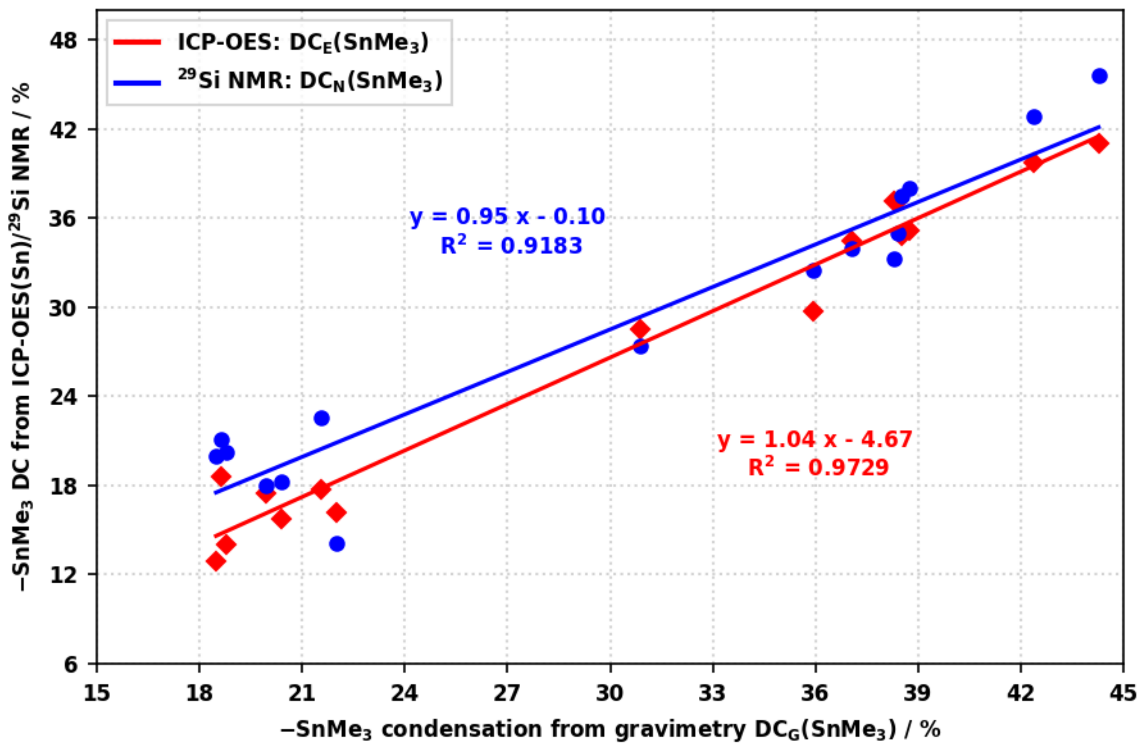

Figure S33. Correlation plot relating the DC of –SnMe$_3$ groups determined by gravimetry/NMR to the values obtained independently from ICP-OES and ^{29}Si MAS NMR deconvolution.

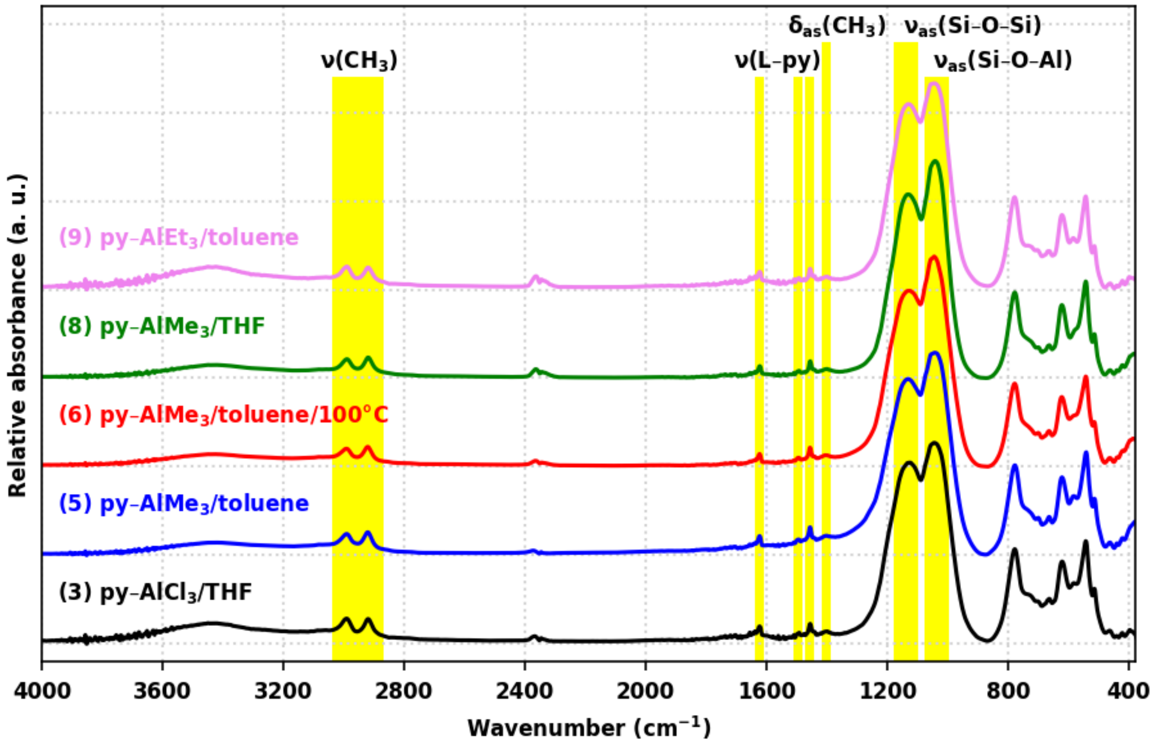

Figure S34. FTIR spectra (KBr pellet) of products prepared from L –AlX$_3$ (X = Me, Et, Cl), illustrating their nearly-identical character.

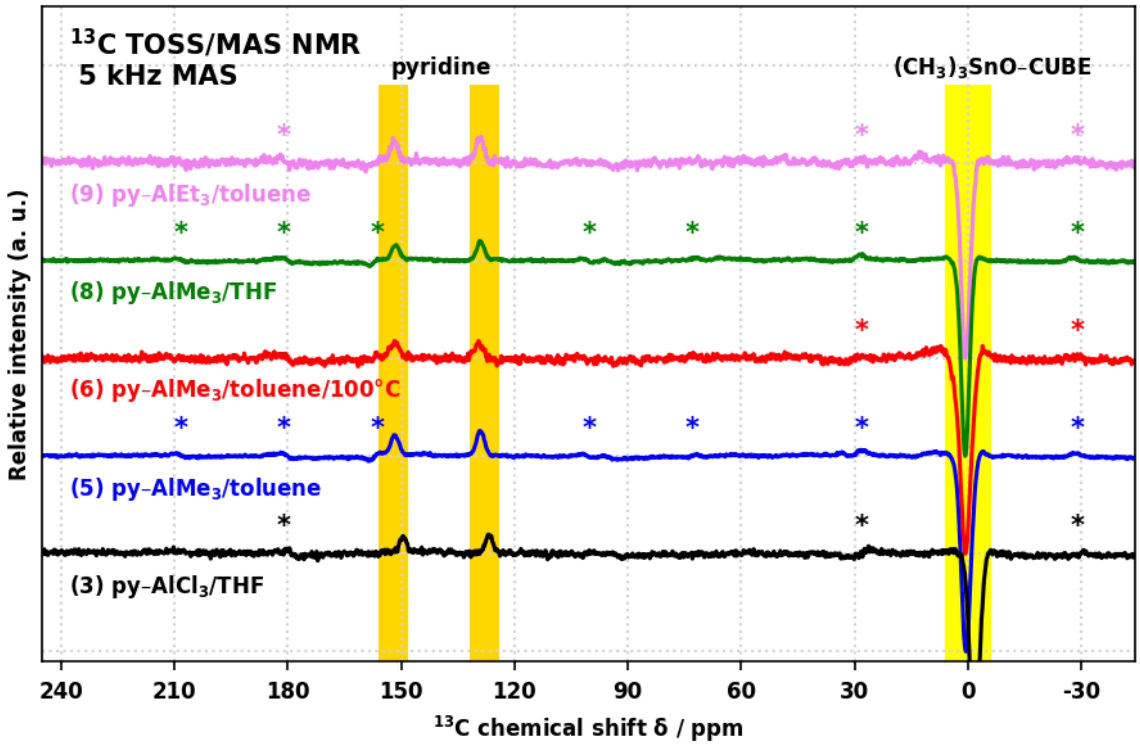

Figure S35. ^{13}C CP/TOSS MAS NMR spectra of products prepared from L –AlX_3 (X = Me, Et, Cl), further illustrating their nearly-identical character. Spinning side-band artifacts are denoted by asterisks.

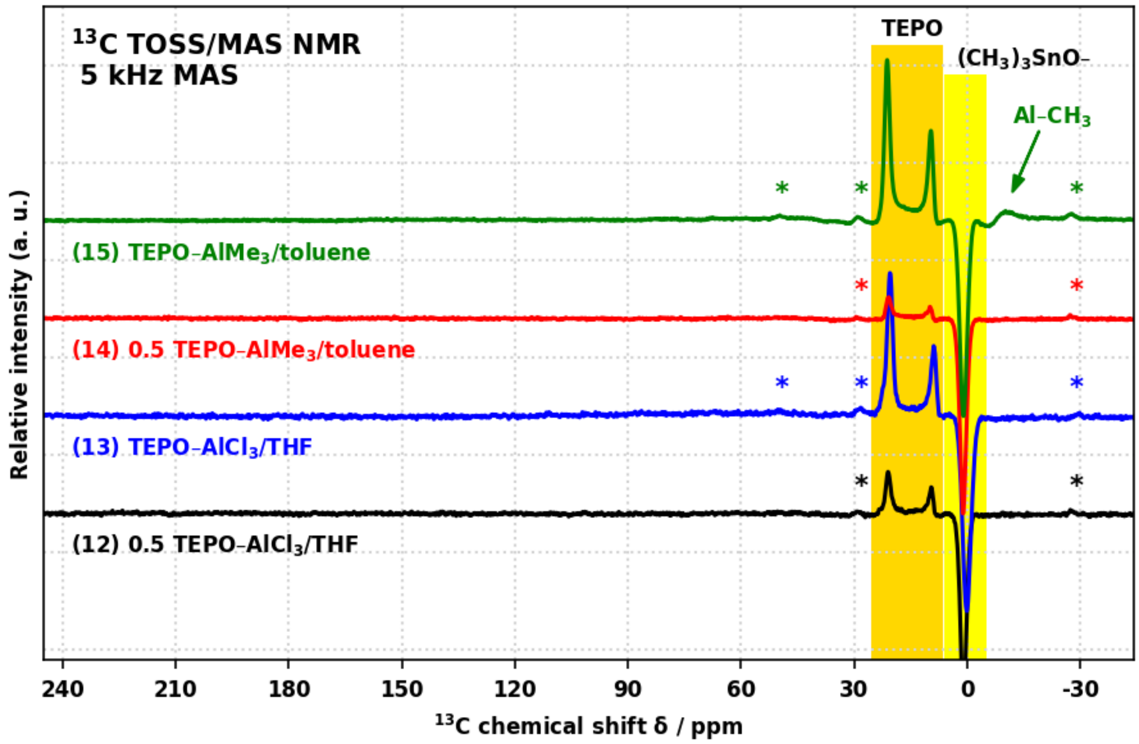

Figure S36. ^{13}C CP/TOSS MAS NMR spectra of products **12–15**. Spinning side-band artifacts are denoted by asterisks.

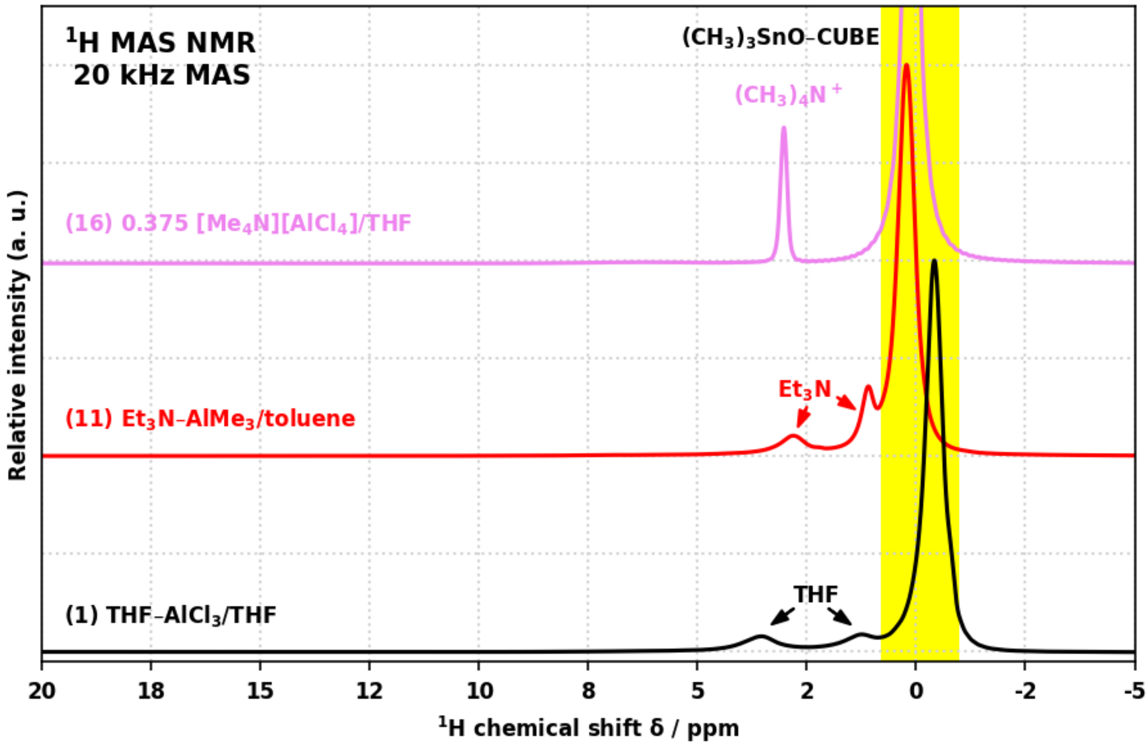

Figure S37. ^{1}H MAS NMR spectra of products **1**, **11**, and **16**.

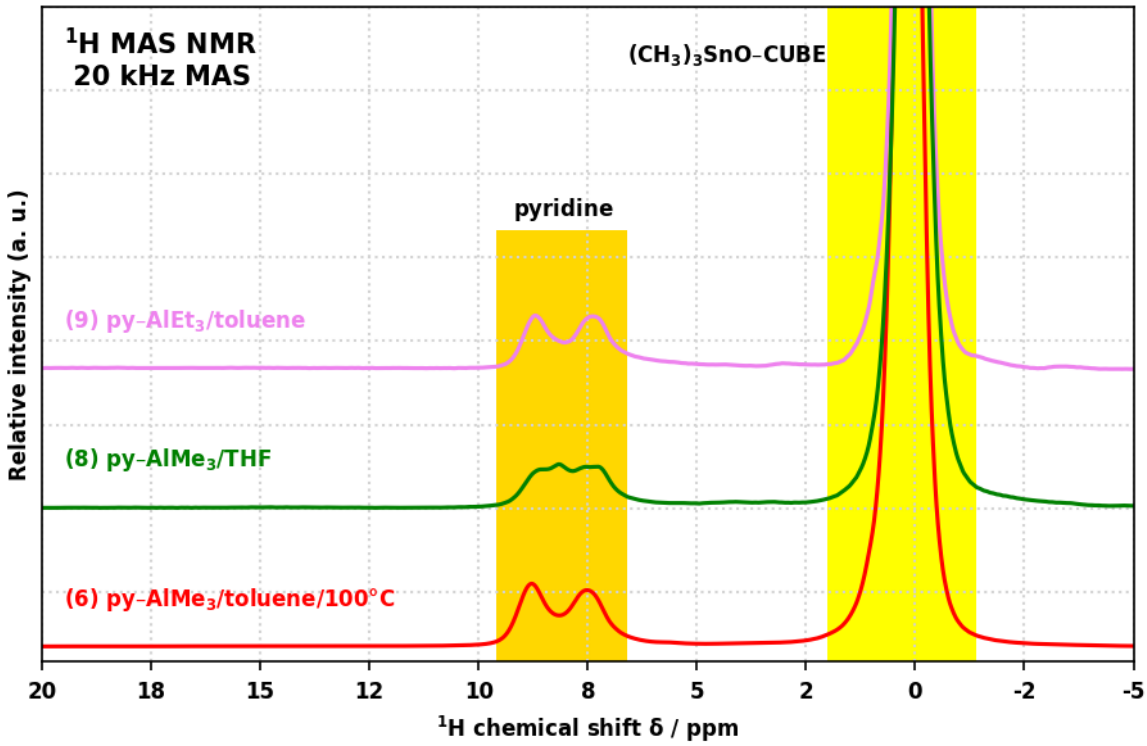

Figure S38. ^{1}H MAS NMR spectra of products **6**, **8**, and **9**.

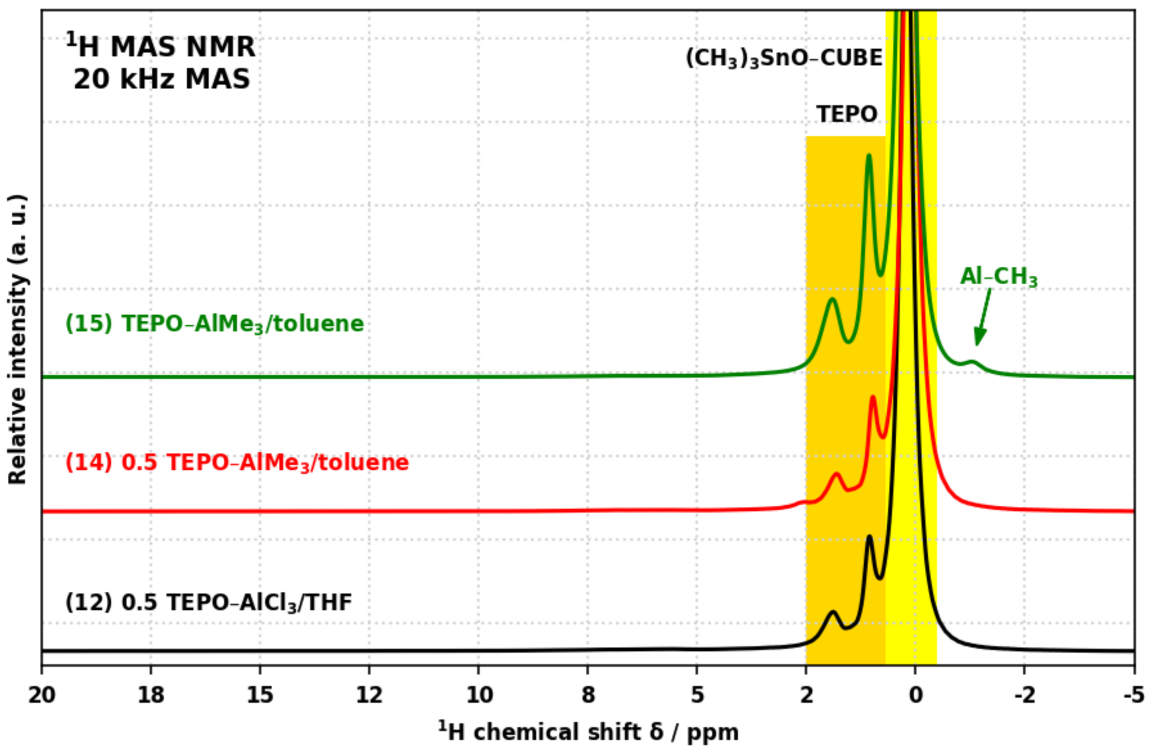

Figure S39. ^{1}H MAS NMR spectra of products **12**, **14**, and **15**.

Table S20. Fitted ^{27}Al NMR parameters and assignments of all observed resonances in products **1–16**.

Site type	Oligomers/Site[a]	δ_{F1} (ppm)	δ_{iso} (ppm)	C_Q (MHz)	η
THF–Al(OCUBE)$_3$	1/A[b]	82.60	38.102	15.144	0.683
THF–Al(O$_2$CUBE)(OCUBE)	1/C[b]	59.60	49.731	12.025	0.100
py–Al(OCUBE)$_3$	2/A	74.90	49.866	11.798	0.745
	3/A	77.22	51.799	13.320	0.670
	4/A	76.50	49.319	12.282	0.630
	5/A	77.60	55.077	14.052	0.652
	6/A	74.50	51.851	12.917	0.772
	7/A[b]	75.12	32.086	9.305	0.500
	9/A	77.90	55.591	13.928	0.624
py–Al(O$_2$CUBE)(OCUBE)	2/C	61.47	57.981	9.002	0.424
	3/C	61.92	61.724	8.918	0.429
	4/C	63.00	56.218	8.395	0.434
	5/C	61.05	63.151	8.580	0.700
	7/C	59.41	61.315	8.862	0.404
	8/A	63.60	64.351	8.015	0.674
	9/C	64.70	57.767	9.427	0.502
Et$_3$N–Al(OCUBE)$_3$	10/A	69.10	44.918	10.736	0.861
	11/A	70.99	51.429	12.051	0.835
TEPO–Al(OCUBE)$_3$	12/A	59.20	49.897	10.120	0.481
	13/A	59.85	50.976	10.525	0.405
	14/A	59.41	54.080	10.805	0.375
	15/A	58.17	48.313	10.259	0.461
TEPO–AlMe(OCUBE)$_2$	15/C[b]	99.94	66.908	12.822	0.439
$[AlO_4]^-$	1/B	49.70	59.047	6.139	0.625
	2/B	51.13	52.180	4.814	0.526
	2/D	57.90	58.605	6.296	0.426
	3/B	53.23	58.552	6.195	0.690
	4/B	55.60	55.418	5.923	0.364
	5/B	56.92	59.205	6.281	0.532
	6/B	49.50	56.519	5.591	0.650
	6/C	52.70	58.585	5.161	0.821
	6/D	56.60	59.466	4.921	0.549
	7/B	52.37	59.408	6.082	1.000
	8/B	56.90	57.282	5.804	0.351
	9/B	56.00	57.030	6.079	0.482
	10/B	55.13	57.615	5.136	0.500
	11/B	54.44	57.088	6.030	0.540
	15/B	52.09	58.613	6.260	0.600
	16/A	49.20	51.683	4.444	0.477
	16/B	53.10	55.349	4.542	0.508
	16/C	57.70	59.231	5.177	0.504

[a] As labelled in TQ/MAS slice fitting.

[b] Poor quality of fit. Data of 7/A excluded from the calculation of the average characteristics.

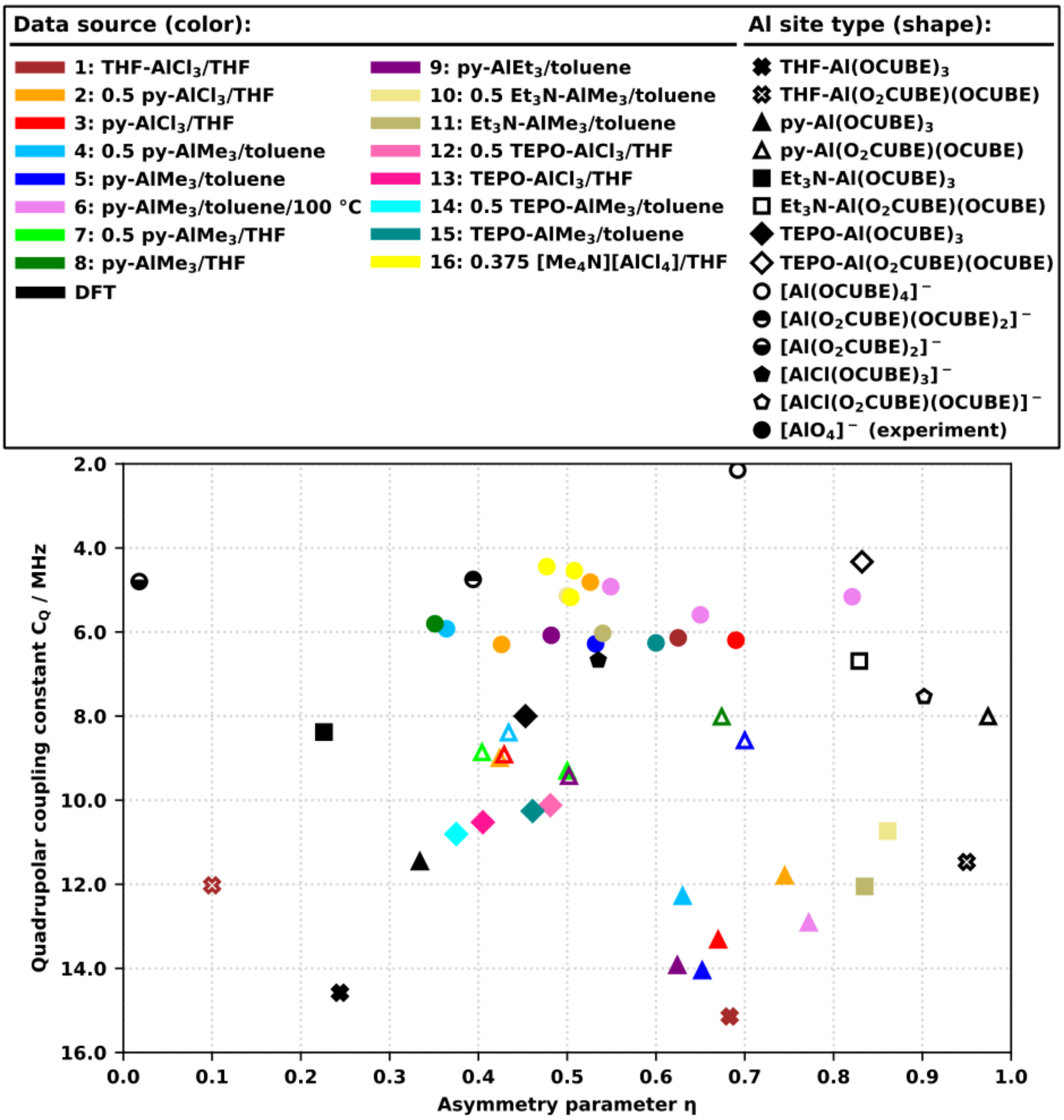

Figure S40. The plot of all observed ^{27}Al TQ/MAS NMR resonances and relevant DFT-calculated models in the η/C_Q parameter space. The shape denotes the assigned type of site while the color points to the source of the data (product spectra or calculation).

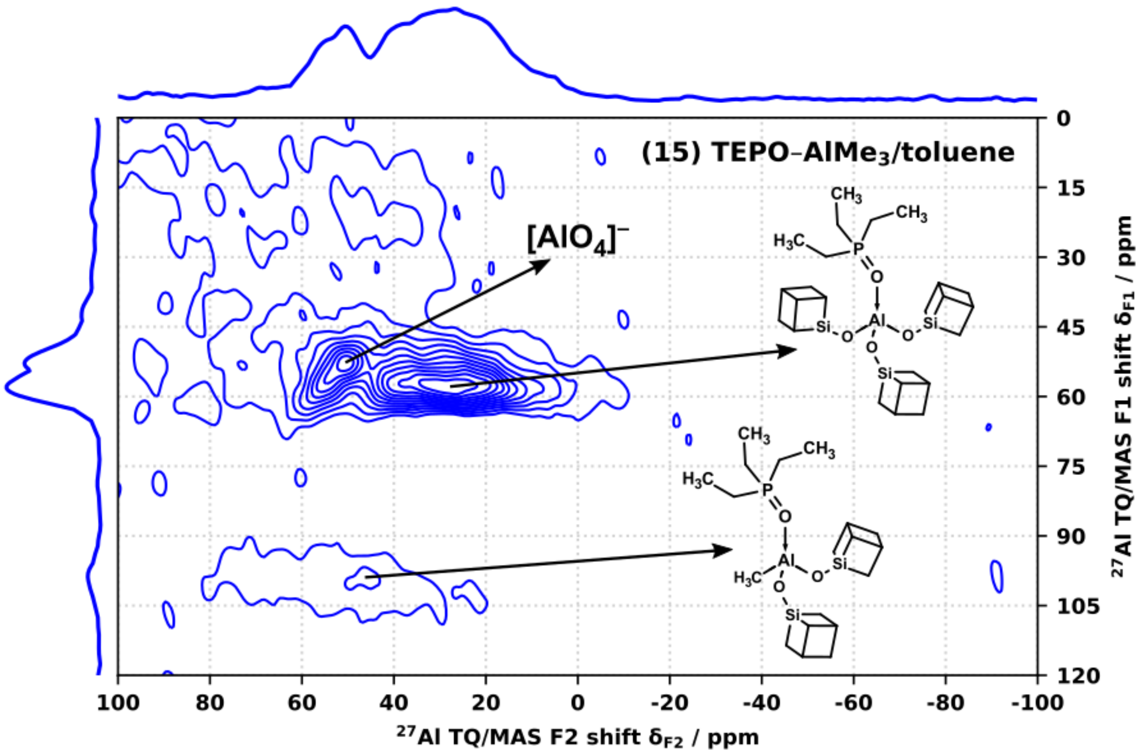

Figure S41. ^{27}Al TQ/MAS NMR spectrum of product **15** (20 kHz MAS).

S4. ^{27}Al NMR parameter calculation data

Table S21. DFT-calculated ^{27}Al ssNMR parameters of model structures with CUBEs terminated by –OMe.

**Structure (CUBEs terminated by –OMe)**	**δ_{iso} (ppm)**	**C_Q (MHz)**	**η**	**Ω (ppm)**	**κ**	**α (°)**	**β (°)**	**γ (°)**
THF–Al(OCUBE)$_3$	47.9	14.58	0.244	73.9	-0.51	354	62	79
THF–Al(O$_2$CUBE)(OCUBE)	55.4	11.47	0.950	78.3	0.18	306	89	66
THF–Al(OCUBE)$_2$Cl	66.2	-12.14	0.980	69.4	-0.37	122	46	336
THF–Al(OCUBE)$_2$Me	89.1	19.82	0.904	136.9	0.39	256	63	68
py–Al(OCUBE)$_3$	52.9	11.46	0.334	75.3	-0.28	248	86	126
py–Al(O$_2$CUBE)(OCUBE)	60.0	-8.01	0.974	62.1	0.14	4	73	307
py–Al(OCUBE)$_2$Cl	71.4	-9.58	0.805	70.5	-0.27	228	34	61
py–Al(OCUBE)$_2$Me	93.8	-17.27	0.886	137.5	0.31	296	88	109
TEPO–Al(OCUBE)$_3$	48.5	8.00	0.453	40.4	-0.56	60	65	225
TEPO–Al(O$_2$CUBE)(OCUBE)	51.8	-4.33	0.832	20.1	0.38	239	65	67
TEPO–Al(OCUBE)$_2$Cl	62.0	8.38	0.779	43.6	-0.84	47	57	228
TEPO–Al(OCUBE)$_2$Me	84.5	-14.93	0.614	101.8	0.59	141	54	147
Et$_3$N–Al(OCUBE)$_3$	53.1	8.38	0.226	43.6	-0.65	349	58	85
Et$_3$N–Al(O$_2$CUBE)(OCUBE)	64.0	-6.69	0.829	38.3	0.10	68	86	254
Et$_3$N–Al(OCUBE)$_2$Cl	73.2	-8.95	0.764	42.5	-0.28	217	110	286
Et$_3$N–Al(OCUBE)$_2$Me	99.5	-13.11	0.940	86.4	0.42	256	12	273
[Al(OCUBE)$_4$]$^–$	51.7	2.15	0.692	16.0	0.23	270	67	264
[Me$_4$N]$^+$ [Al(OCUBE)$_4$]$^–$	50.7	-2.04	0.884	7.0	0.24	50	26	241
[Me$_3$Sn]$^+$ [Al(OCUBE)$_4$]$^–$	57.9	12.69	0.336	51.0	-0.48	232	69	310
[py–SnMe$_3$]$^+$ [Al(OCUBE)$_4$]$^–$	56.7	6.31	0.314	19.8	-0.66	297	109	217
[Al(OCUBE)$_3$Cl]$^–$	63.2	6.67	0.535	21.0	0.05	149	80	297
[Al(O$_2$CUBE)(OCUBE)$_2$]$^–$	52.3	4.75	0.394	27.4	-0.63	335	109	251
[Me$_4$N]$^+$ [Al(O$_2$CUBE)(OCUBE)$_2$]$^–$	53.0	5.03	0.579	26.4	-0.60	343	65	106
[Me$_3$Sn]$^+$ [Al(O$_2$CUBE)(OCUBE)$_2$]$^–$	54.7	13.29	0.160	49.1	-0.84	16	55	188
[py–SnMe$_3$]$^+$ [Al(O$_2$CUBE)(OCUBE)$_2$]$^–$	53.2	7.79	0.104	29.2	-0.52	44	89	13
[Al(O$_2$CUBE)(OCUBE)Cl]$^–$	64.8	-7.54	0.902	29.3	0.70	257	12	109
[Al(O$_2$CUBE)$_2$]$^–$	50.9	-4.80	0.018	13.1	0.98	53	74	253

$[Me_4N]^+$ $[Al(O_2CUBE)_2]^-$	53.5	-5.17	0.434	24.0	0.52	97	136	197
$[Me_3Sn]^+$ $[Al(O_2CUBE)_2]^-$	60.4	8.72	0.231	28.2	-0.54	257	106	247
$[py–SnMe_3]^+$ $[Al(O_2CUBE)_2]^-$	59.6	4.36	0.659	17.9	0.31	54	84	300

Table S22. DFT-calculated ^{27}Al ssNMR parameters of selected structures with CUBEs terminated by –OH, $–OSiMe_3$, and $–OSnMe_3$.

**Structure / CUBE termination**	δ_{iso} **(ppm)**	C_Q **(MHz)**	η	Ω **(ppm)**	κ	α **(°)**	β **(°)**	γ **(°)**
$py–Al(OCUBE)_3$ / –OH	55.1	10.05	0.399	70.4	-0.46	349	38	100
$py–Al(OCUBE)_3$ / $–OSiMe_3$	55.8	12.24	0.247	80.6	-0.57	260	142	236
$py–Al(OCUBE)_3$ / $–OSnMe_3$	50.4	13.46	0.219	75.7	-0.71	351	85	197
$Et_3N–Al(OCUBE)_3$ / –OH	57.1	8.48	0.296	39.9	-0.66	300	77	123
$Et_3N–Al(OCUBE)_3$ / $–OSiMe_3$	54.0	10.33	0.350	52.8	-0.68	287	35	122
$Et_3N–Al(OCUBE)_3$ / $–OSnMe_3$	53.9	11.00	0.220	48.8	-0.90	48	76	249
$[Al(OCUBE)_4]^-$ / –OH	45.7	-2.92	0.325	20.2	0.43	158	79	119
$[Al(OCUBE)_4]^-$ / $–OSiMe_3$	41.6	1.49	0.737	8.0	-0.06	321	39	79
$[Al(OCUBE)_4]^-$ / $–OSnMe_3$	44.4	3.05	0.747	11.1	-0.18	219	19	18

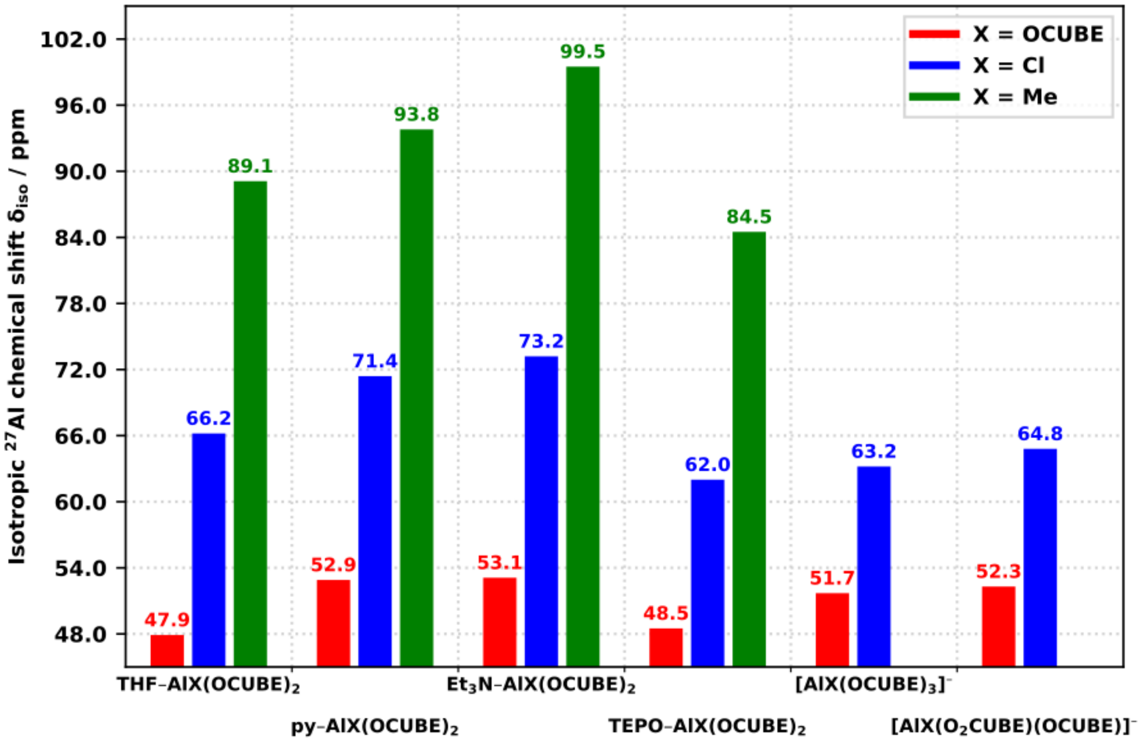

Figure S42. Comparison of predicted δ_{iso} for various sites containing residual Al–Cl and Al–Me groups.

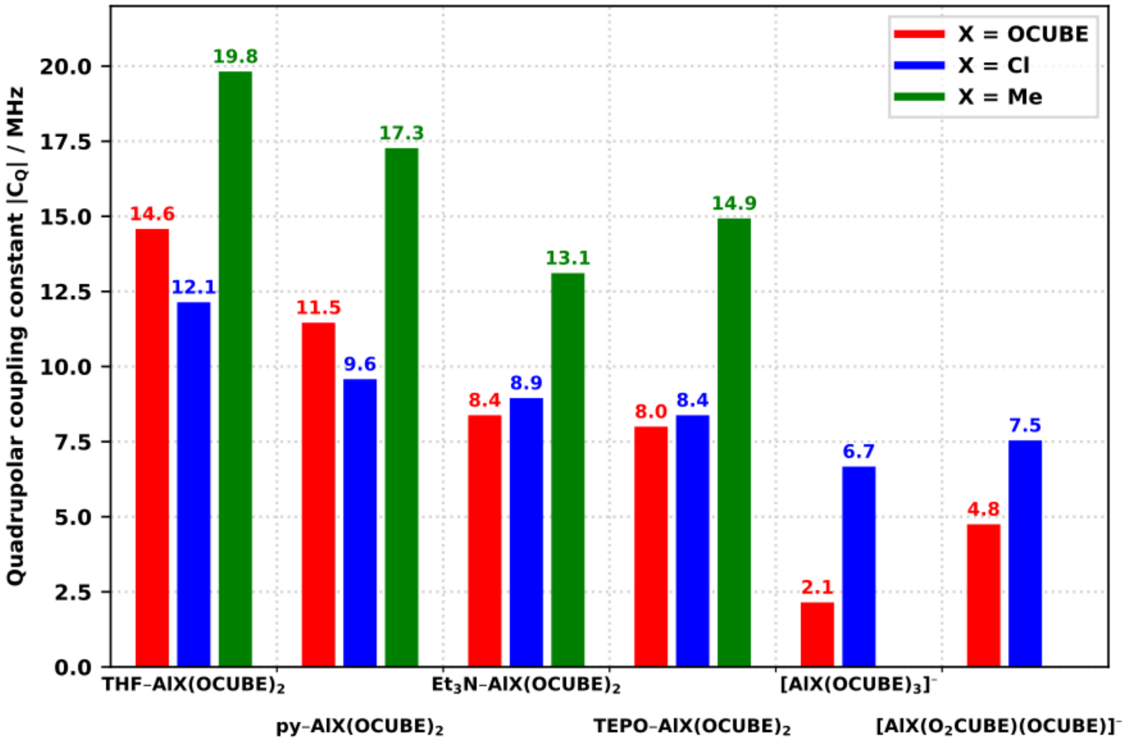

Figure S43. Comparison of predicted $|C_Q|$ for various sites containing residual Al–Cl and Al–Me groups.

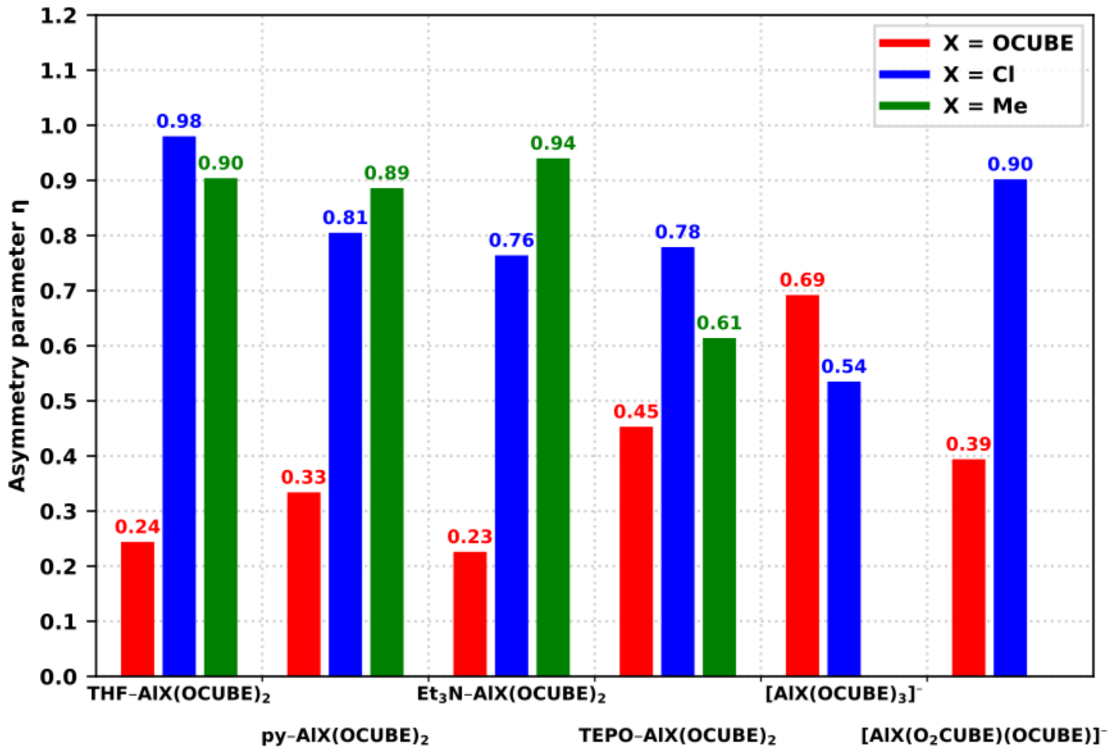

Figure S44. Comparison of predicted η for various sites containing residual Al–Cl and Al–Me groups.

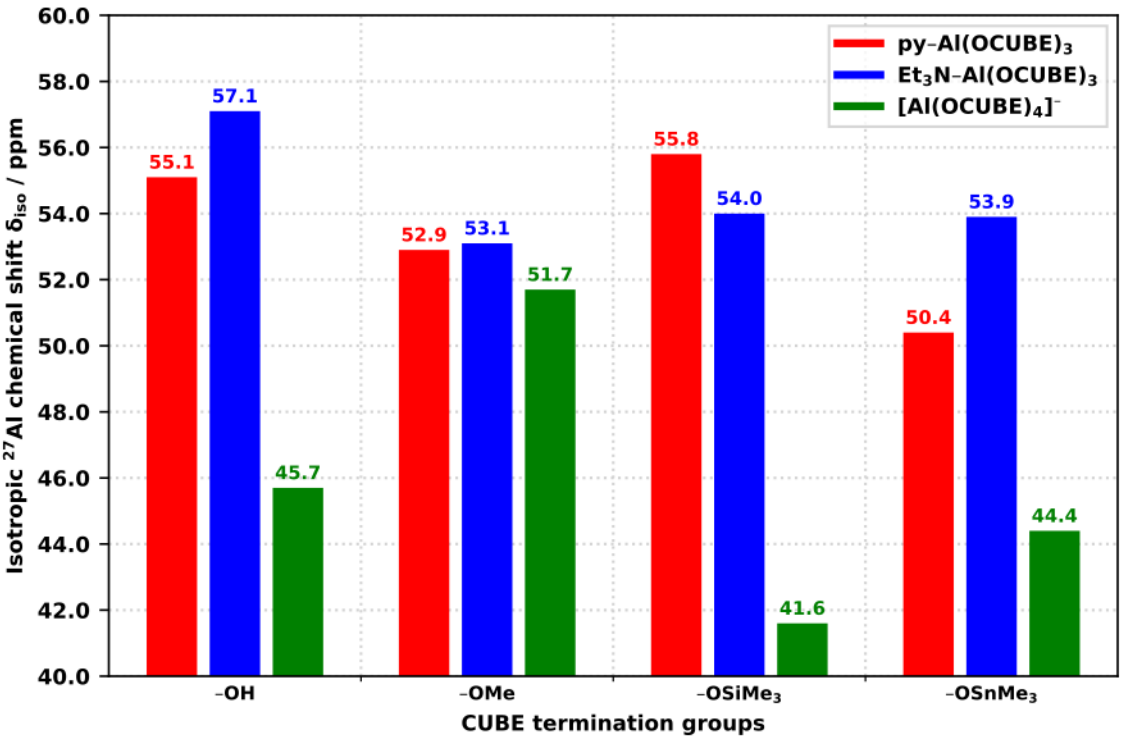

Figure S45. Comparison of predicted δ_{iso} for selected sites with CUBEs terminated by –OH, –OMe, –$OSiMe_3$, and –$OSnMe_3$ groups.

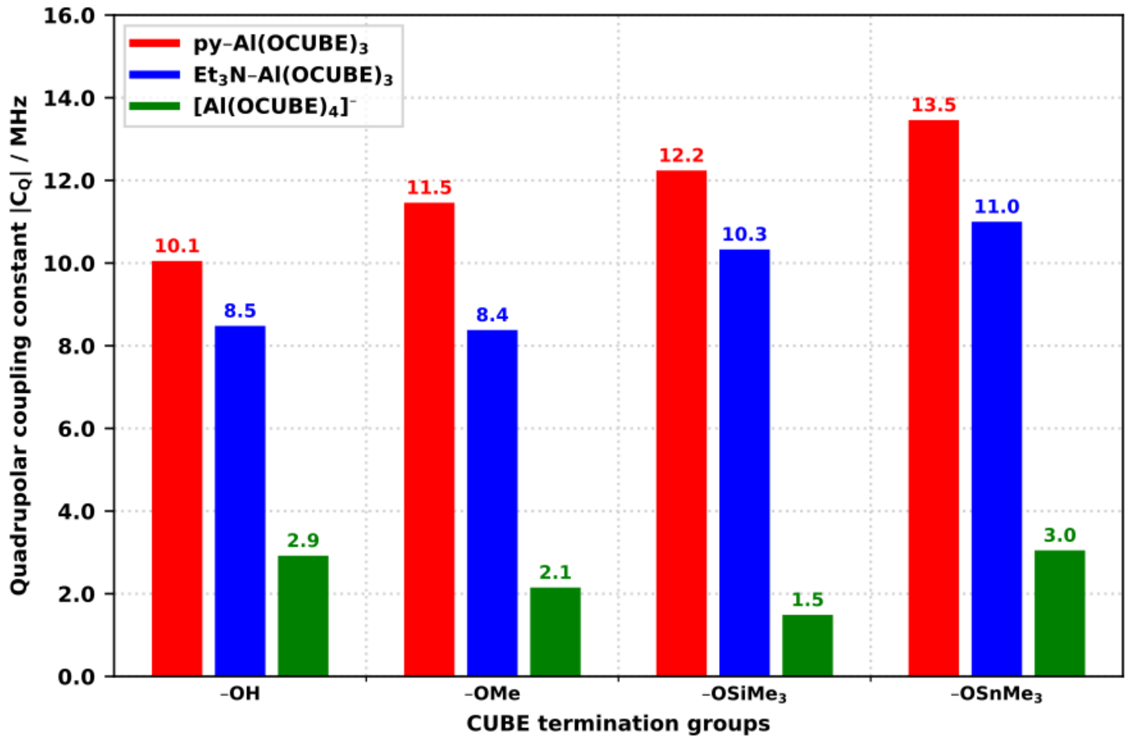

Figure S46. Comparison of predicted $|C_Q|$ for selected sites with CUBEs terminated by –OH, –OMe, –OSiMe$_3$, and –OSnMe$_3$ groups.

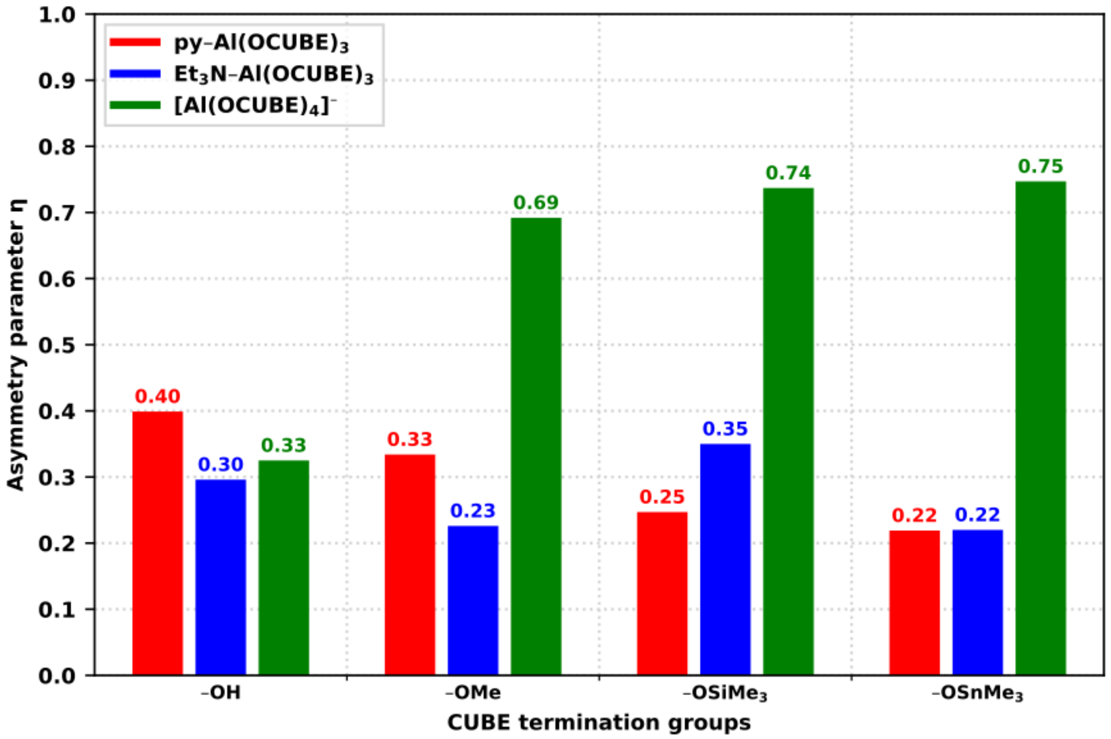

Figure S47. Comparison of predicted η for selected sites with CUBEs terminated by –OH, –OMe, –OSiMe$_3$, and –OSnMe$_3$ groups.

S6. References

(1) Gottlieb, H. E.; Kotlyar, V.; Nudelman, A. NMR Chemical Shifts of Common Laboratory Solvents as Trace Impurities. *J. Org. Chem.* **1997**, *62* (21), 7512–7515. https://doi.org/10.1021/jo971176v.

(2) Brunauer, S.; Emmett, P. H.; Teller, E. Adsorption of Gases in Multimolecular Layers. *J. Am. Chem. Soc.* **1938**, *60* (2), 309–319. https://doi.org/10.1021/ja01269a023.

(3) Lowell, S.; Shields, J. E.; Thomas, M. A.; Thommes, M. *Characterization of Porous Solids and Powders: Surface Area, Pore Size and Density*; Particle Technology Series; Springer Netherlands: Dordrecht, 2004; Vol. 16. https://doi.org/10.1007/978-1-4020-2303-3.

(4) *Adsorption by Powders and Porous Solids*; Elsevier, 2014. https://doi.org/10.1016/C2010-0-66232-8.
